# Subtle Structural Differences Trigger Inhibitory Activity of Propafenone Analogues at the Two Polyspecific ABC Transporters: P‐Glycoprotein (P‐gp) and Breast Cancer Resistance Protein (BCRP)

**DOI:** 10.1002/cmdc.201500592

**Published:** 2016-03-10

**Authors:** Theresa Schwarz, Floriane Montanari, Anna Cseke, Katrin Wlcek, Lene Visvader, Sarah Palme, Peter Chiba, Karl Kuchler, Ernst Urban, Gerhard F. Ecker

**Affiliations:** ^1^ Department of Pharmaceutical ChemistryFaculty of Life SciencesUniversity of ViennaAlthanstraße 141090ViennaAustria; ^2^ Department of Medicinal ChemistryMedical University ViennaWähringerstraße 101090ViennaAustria; ^3^ Department of Medical BiochemistryMax F. Perutz LaboratoriesMedical University ViennaDr. Bohr-Gasse 9/21030ViennaAustria

**Keywords:** breast cancer resistance protein, inhibitor, p-glycoprotein, polypharmacology, propafenone

## Abstract

The transmembrane ABC transporters P‐glycoprotein (P‐gp) and breast cancer resistance protein (BCRP) are widely recognized for their role in cancer multidrug resistance and absorption and distribution of compounds. Furthermore, they are linked to drug–drug interactions and toxicity. Nevertheless, due to their polyspecificity, a molecular understanding of the ligand‐transporter interaction, which allows designing of both selective and dual inhibitors, is still in its infancy. This study comprises a combined approach of synthesis, in silico prediction, and in vitro testing to identify molecular features triggering transporter selectivity. Synthesis and testing of a series of 15 propafenone analogues with varied rigidity and basicity of substituents provide first trends for selective and dual inhibitors. Results indicate that both the flexibility of the substituent at the nitrogen atom, as well as the basicity of the nitrogen atom, trigger transporter selectivity. Furthermore, inhibitory activity of compounds at P‐gp seems to be much more influenced by logP than those at BCRP. Exploiting these differences further should thus allow designing specific inhibitors for these two polyspecific ABC‐transporters.

## Introduction

P‐glycoprotein (P‐gp, ABCB1) and the breast cancer resistance protein (BCRP, ABCG2) belong to the superfamily of ABC proteins. In humans, this superfamily is composed of 49 members, subdivided into 7 subfamilies named ABCA to ABCG.^1^ Based on their structural organization, ABC transporters form a passageway across cellular membranes enabling the transport of substrates. They consist of two transmembrane (TMDs) and two nucleotide binding domains (NBDs), whereby ATP hydrolysis at the NBDs provides the energy necessary for the transport process.^1^

P‐gp and BCRP are efflux transporters, mediating transport of their substrates out of cells in which they are expressed. Thus, they are physiologically expressed in organs with barrier and/or excretion functions including the gastrointestinal (GI) tract, the blood‐brain barrier, the liver, and the kidney.^2^

In the GI tract, both ABC family members are located in the apical membrane of enterocytes, facing towards the intestinal lumen, affecting the absorption of compounds from the intestinal lumen into the blood. Similarly, P‐gp and BCRP are localized at the apical side of proximal tubule cells and hepatocytes in the kidney and the liver, respectively. There, they are involved in the excretion of endogenous and exogenous compounds, as well as of metabolites, into the urine or the bile.^3^ At the blood–brain barrier, P‐gp and BCRP are located at the apical/luminal side in the endothelial cells of brain capillaries, where they protect the brain from xenobiotics.^3^

Based on their physiological expression, P‐gp and BCRP are important determinants in absorption, distribution, and excretion of drugs and metabolites that are P‐gp and/or BCRP substrates. Inhibition of the proper transport function of these ABC proteins has been reported to be responsible for drug–drug interactions.^4^ In addition, P‐gp and BCRP are also known for their involvement in multidrug resistance (MDR).^5^ As efflux transporters for different anticancer agents, including anthracyclines, kinase inhibitors (imatinib), and alkaloids (e.g. topotecan),^6^ P‐gp and/or BCRP overexpression increases the transport of these agents out of the tumor cells resulting in decreased drug response.^5^

With the idea to tackle MDR, several medicinal chemistry efforts have already been taken to create compounds that would specifically inhibit either P‐gp or BCRP individually, or in combination. Brooks and colleagues^7^ designed and tested 18 taxane derivatives for their inhibition of P‐gp, MRP1, and BCRP. They found that taxane derivatives are substrates for P‐gp, and to a lesser extent for MRP1, explaining their MDR reversal effect by competitive binding. With respect to BCRP, however, the inhibition was found to be noncompetitive, and the authors suggested that taxane derivatives only bind to one binding site on BCRP and are not transported. Colabufo and colleagues^8^ were looking for selective P‐gp inhibitors since they expected that the in vivo toxicity of existing MDR modulating agents is related to the inhibition of other ABC‐transporters that are instrumental in protecting noncancerous cells. They synthesized a series of arylmethoxy and arylmethylaminederivatives that showed good P‐gp inhibition. Specificity for P‐gp as compared with BCRP seemed to be conferred by the linker, that is, only arylmethylamine derivatives showed activity for BCRP. Sim and colleagues^9^ focused on a type of flavonoids, aurones, which are known inhibitors of BCRP and tried to probe into the selectivity profile towards P‐gp by generating more than one hundred derivatives. While most compounds preferentially inhibited BCRP, this publication found methoxyaurones, methoxyindanones, and methoxyflavones to be active on both P‐gp and BCRP. Kühnle et al^10^ found that by modifying the P‐gp inhibitor tariquidar, they could greatly increase the selectivity for BCRP. Valdameri et al synthesized a series of chalcone derivatives^11^ and chromone derivatives^[12^] aiming at selective BCRP inhibitors. In the case of the chalcones, they showed the influence of the position of methoxy substitutions on both activity and cytotoxicity. While investigating the chromone derivatives, they found a very selective and active derivative for BCRP with a high therapeutic index. Also in case of propafenones, both compounds showing P‐gp and BCRP specificity could be identified.^13^ Here, we extend these studies and aim to understand the molecular features triggering transporter selectivity. For this, we combined synthesis of novel propafenone derivatives, in silico prediction models to prioritize our compounds, and in vitro activity measurements.

## Results and Discussion

**Figure 1:**
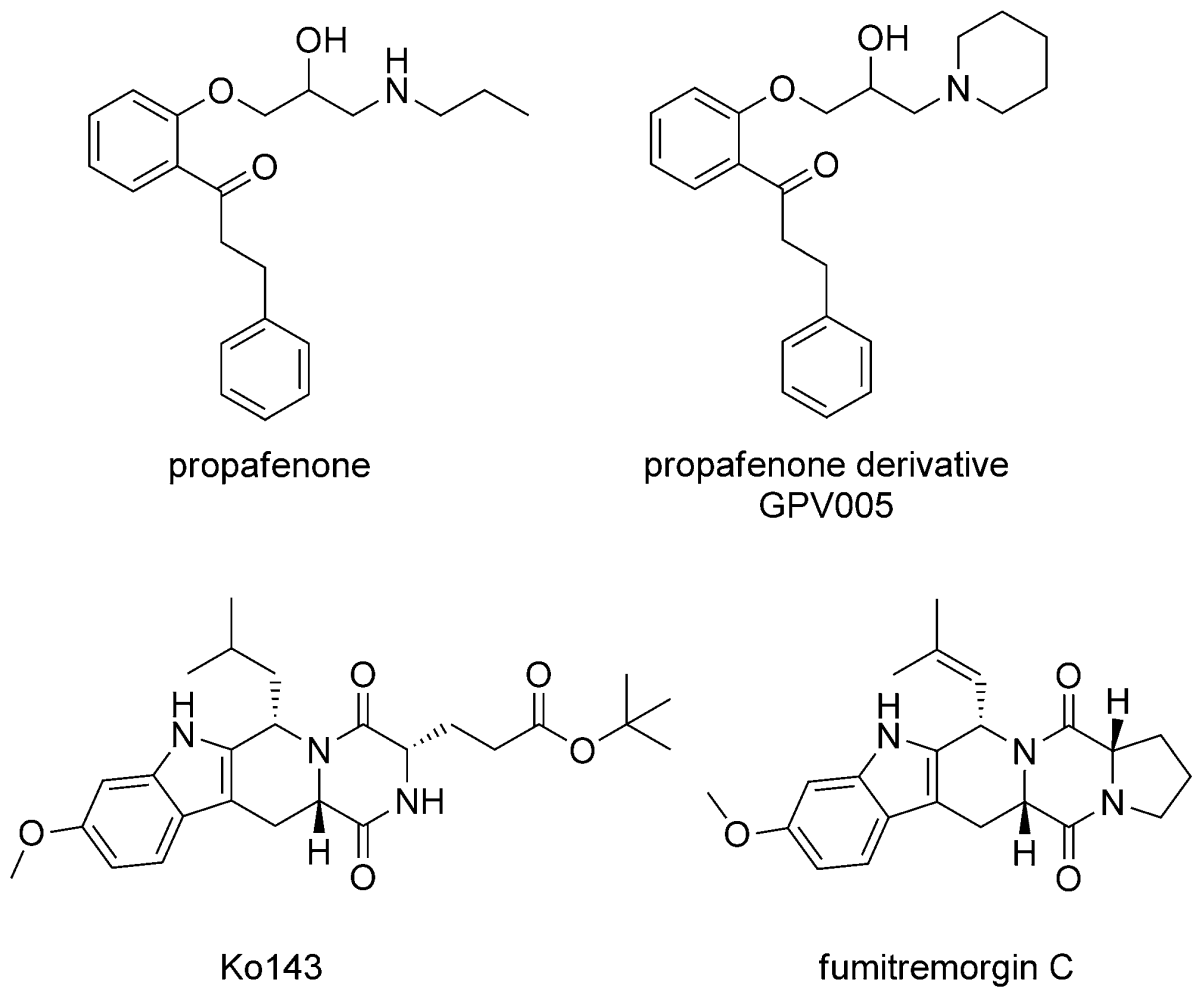
P‐gp and BCRP inhibitors.

Both P‐glycoprotein and BCRP show polyspecific ligand recognition patterns with considerable overlap in their substrate and inhibitor profile. Previous results obtained with propafenone‐type inhibitors pointed towards the vicinity of the nitrogen atom as potential structural moiety for selectivity profiling. In particular, compounds that contain a nonionizable nitrogen atom showed high selectivity indices. Furthermore, also the number of rotatable bonds as well as the number of H‐bond acceptors seemed to influence both activity and selectivity.^13^ Thus, the compound design focused on two main features, the basicity of the nitrogen atom as well as the rigidity of the substituents in this region. This was also inspired by the polycyclic structure of fumitremorgin C and its derivative Ko143, which are selective BCRP inhibitors (Figure 1).

### In silico classification models for P‐gp and BCRP

In order to assess the likelihood of success, in silico classification models for P‐gp and BCRP were established, and the inhibitor profiles of the compounds of interest were predicted. Two binary classification models for BCRP and P‐gp inhibition were built on previously published large datasets.^14^ Both models are based on ECFP‐like (extended connectivity fingerprint) 1024‐bit fingerprints as descriptors and use logistic regression (for BCRP) and support vector machine (for P‐gp) as base classifiers. The models are classification models, thus predicting whether a compound is likely to be an inhibitor of any of the two transporters. As threshold for active/inactive an IC_50_ value of 10 μm was used. The predictive capability of the two models was evaluated by classical 10‐fold cross‐validation, results of which are shown in Table 1.

**Table 1:** Evaluation of the predictive power of the in silico models by 10‐fold cross‐validation.^[a]^

	Accuracy	Sensitivity	Specificity	MCC	AUC
BCRP inhibition	0.83	0.77	0.87	0.65	0.90
P‐gp inhibition	0.87	0.86	0.89	0.75	0.94

[a] Accuracy: the amount of correct predictions among all predictions; sensitivity: the proportion of inhibitors correctly identified; specificity: the proportion of noninhibitors correctly identified; Matthews correlation coefficient (MCC): correlation coefficient between the real and predicted classifications (between −1 and 1); area under the receiver operating characteristic (ROC) curve (AUC): ranking capability of the model.

**Figure 2:**
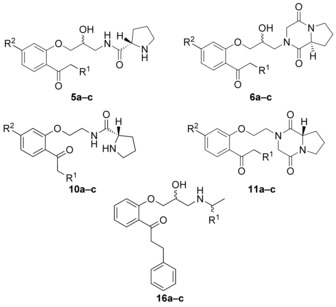
Synthesized target structures.

The two models were used to subsequently predict the inhibition against BCRP and P‐gp for a series of compounds (Figure 2). As outlined above, transporter selectivity for propafenones might be triggered by the substituents in the vicinity of the nitrogen atom. Flexibility of the substituents was triggered by the introduction of a proline (compounds **5** and **10**) and a diketopiperazine moiety (compounds **6** and **11**), whereby the latter contained only nonbasic nitrogen atoms. Furthermore, also lipophilicity of the compounds was varied by different substituents at the phenone moiety (R^1^). Finally, the linker region between the nitrogen atom and the central aromatic ring (compound **5** vs. **10** and **6** vs. **11**), as well as the substitution on the central aromatic ring, was modified. In order to specifically analyze the influence of the basicity of the nitrogen atom on the transporter interaction profile of propafenones, the respective isopropyl, fluoroisopropyl, and difluoroisopropyl analogues **16 a–c** were synthesized. The predictions for the whole set of compounds indicate that both dual inhibitors as well as selective P‐gp inhibitors should be present in this compound series.

### Synthesis

The target compounds **5, 6, 10**, and **11** (Figure 2) were synthesized following two general strategies.

**Scheme 1:**
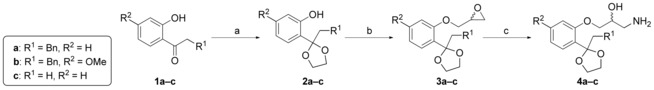
Synthesis of intermediate products **4 a–c**. *Reagents and conditions*: a) ethylene glycol, trifluoromethanesulfonic acid (TfOH), trimethylorthoformate, hexane, 40 °C, 3 d, 63–99 %; b) epichlorohydrine, NaH, DMF, 70 °C, o/n, 74–99 %; c) NH_3_ in MeOH, RT, 2 d 14–54 %.

Respective hydroxyphenones **1 a–c** were prepared from 1‐(2‐hydroxy‐4‐methoxyphenyl)ethanone by condensation with benzaldehyde and reduction of the double bond by hydrogenation.^15^ For the synthesis of the aryloxypropanolamines **4** and **6**, the ketone functionality in differently substituted 2′‐hydroxyacetophenones **1 a–c** was protected as a ketal using ethylene glycol and trimethylorthoformate similar to Ref. 16 as shown in Scheme 1. Subsequently, the phenols **2 a–c** were O‐alkylated with epichlorohydrine, and the epoxides **3 a–c** were reacted with ammonia in methanol to yield the aryloxypropanolamines **4 a–c**. Attempts to avoid the step of the protection of the ketone group failed and led to complex mixtures after the epoxide ring opening with ammonia.

**Scheme 2:**
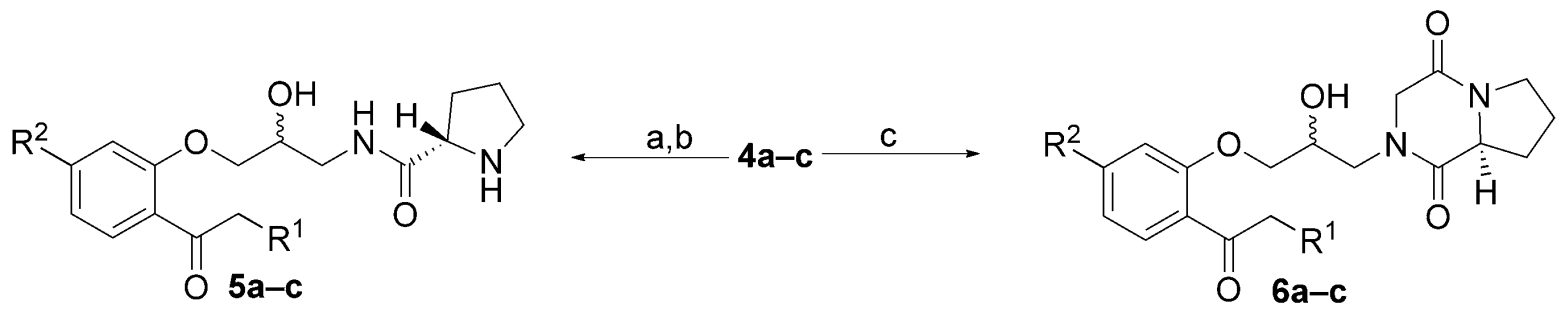
Synthetic route for final compounds **5 a–c** and **6 a–c**. *Reagents and conditions*: a) boc‐proline, DCC, DMAP, CH_2_Cl_2_, 2 h to o/n, 41–71 %; b) 4 n HCl, dioxane, RT, 4 h, 98 %–quant.; c) methyl (2‐chloroacetyl)prolinate, Et_3_N, RT, CH_3_CN, 2.5 d or reflux, 2‐ethoxyethanol, 2–3 d, then 2 n HCl, CH_3_CN, RT, 4 h, 6–61 % (2 steps).

Amines **4 a–c** were converted to the respective amides **5 a–c** with *N*‐(tert‐butoxycarbonyl)‐l‐proline (boc‐proline) using dicyclohexylcarbodiimide (DCC) and 4‐dimethylaminopyridine (DMAP) (see Scheme 2). Unfortunately, the NMR analysis of the proline derivatives **5 a–c** proved to be difficult due to the occurrence of rotamers that lead to broad signals. In addition, a mixture of diastereomers was formed that could not be separated by column chromatography.

For synthesis of the diketopiperazines **6 a–c**, amines **4 a–c** were reacted with methyl(2‐chloroacetyl)prolinate, which was prepared from proline methyl ester and 2‐chloroacetyl chloride.^17^ The diketopiperazine analogues showed NMR spectra that were easier to interpret due to the fixed conformation. The last step in both synthetic routes was the deprotection of the carbonyl group under acidic conditions, and, for the proline derivatives, formation of a hydrochloride. In some cases, the deprotection of the ketone occurred already partially during the column chromatography step.

**Scheme 3:**
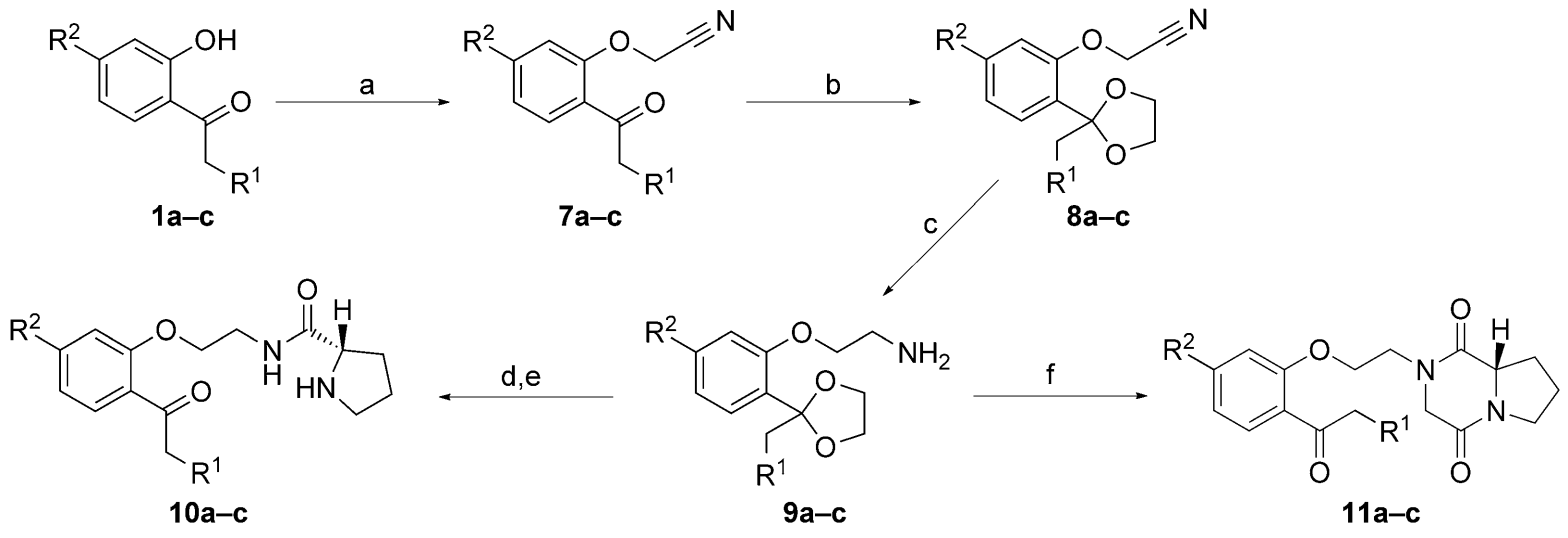
Synthesis of compounds **10 a–c** and **11 a–c**. *Reagents and conditions*: a) bromoacetonitrile, K_2_CO_3_, acetone, 50 °C, 4 d (88–100 %); b) ethylene glycol, *p*‐toluenesulfonic acid (pTosOH), toluene, reflux, o/n, 72–97 %; c) LiAlH_4_ or Red‐Al, Et_2_O, reflux or RT, 3–4 h, 15‐42 %; d) boc‐proline, DCC, DMAP, CH_2_Cl_2_, 2 h to o/n, 57‐78 %; e) 4 n HCl, dioxane, RT, 4 h, 97 %–quant.; f) methyl (2‐chloroacetyl)prolinate, Et_3_N, reflux, 2‐ethoxyethanol, 2 d, then 2 n HCl, CH_3_CN, RT, 4 h, 19–39 % (2 steps).

For the synthesis of the aryloxyethanolamines **10** and **11**, the substituted 2′‐hydroxyphenones **1 a–c** were first reacted with bromoacetonitrile, followed by a protection of the carbonyl group as outlined above (see Scheme 3). Subsequently, the nitriles **8 a–c** were reduced using lithium aluminium hydride^18^ to yield the amines **9 a–c**. Attempts to improve the yield by using other reducing agents, such as Red‐Al (sodium bis(2‐methoxyethoxy)aluminumhydride, used for the synthesis of **9 a**) or borane‐tetrahydrofuran (THF)‐complex failed.

**Scheme 4:**
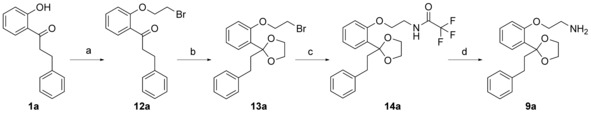
Alternative route to compound **9 a**. *Reagents and conditions*: a) 1,2‐dibromoethane, K_2_CO_3_, Bu_4_NBr, toluene, water, reflux, 6 d, 79 %; b) ethylene glycol, TfOH, trimethylorthoformate, hexane, 40 °C, 3 d, 99 %; c) trifluoroacetamide, K_2_CO_3_, Bu_4_NBr, DMF, 80 °C, 4.5 h, 65 %; d) KOH, MeOH, water, RT, 2 h, 99 %.

An alternative route for the synthesis of intermediate products **9** comprised the O‐alkylation of the phenol **1 a** with 1,2‐dibromoethane^19^ as shown in Scheme 4. Then the carbonyl group of **12 a** is protected with ethylene glycol, and the bromide is substituted with trifluoroacetamide^20^ to give **14 a**. This derivative can easily be cleaved with base, furnishing the amine **9 a** in higher yields and with less tedious steps. The proline amide and diketopiperazine derivatives were synthesized in analogy to the procedures as described above, and after deprotection and, if possible, hydrochloride formation, the target compounds **10 a–c** and **11 a–c** were obtained.

**Scheme 5:**
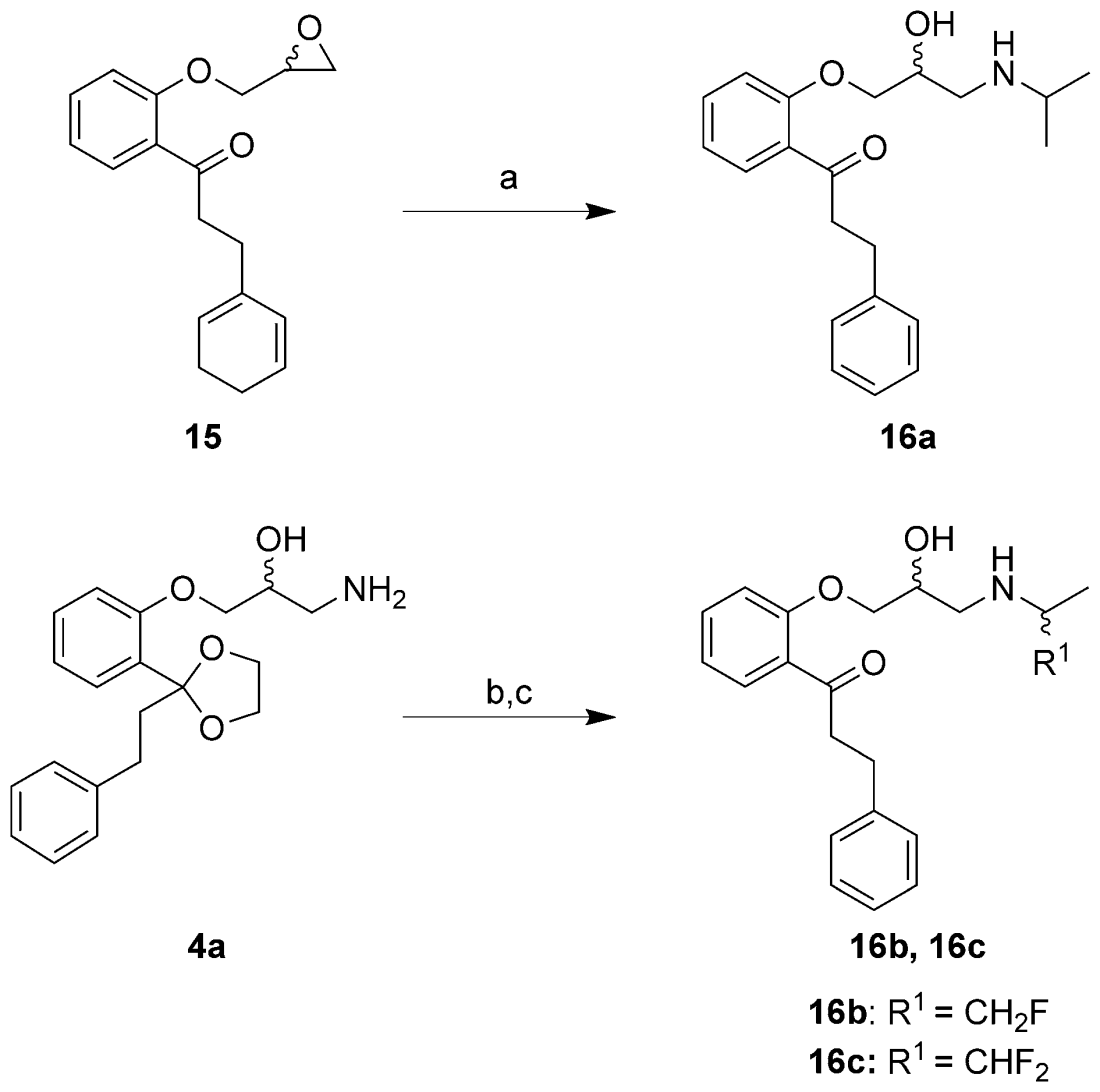
Synthesis of compounds **16 a–c**. *Reagents and conditions*: a) *i*PrNH_2_, reflux, 21 h, 57 %; b) NaBH_3_CN, mono or difluoroacetone, AcOH, MeOH, reflux, 4 h, quant.; c) 2 n HCl, EtOAc, 2 h, 84–86 %.

Following the standard synthetic route for propafenone derivatives, compound **16 a** was prepared by reacting the epoxide **15** with isopropylamine. For the fluorinated derivatives **16 b** and **16 c**, this was not possible due to the decreased basicity and thus reactivity of the fluorinated amines. Therefore the amine **4 a** was reacted with the respective fluorosubstituted acetone derivatives in a reductive amination reaction followed by deprotection of the ketone (Scheme 5).

### In vitro studies

**Figure 3:**
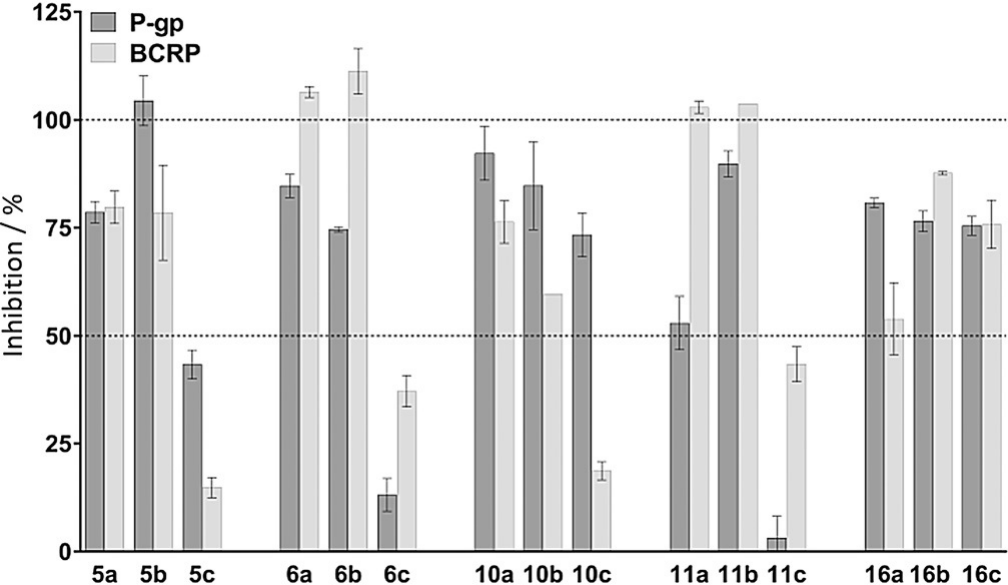
Evaluation of compounds used for IC_50_ measurements. In a first set of experiments, the effect of all compounds at 100 μm final concentration was tested on P‐gp (dark grey bars) and BCRP (light grey bars) using intracellular accumulation assay as described in the Experimental Section. Data are shown as % inhibition compared with the positive control verapamil (P‐gp) or Ko143 (BCRP) and are given as mean±SD of a single experiment with at least duplicate measurements.

P‐gp and BCRP inhibitory activity was assessed by implementing an intracellular accumulation assay of daunorubicin or mitoxantrone in cells overexpressing P‐gp or BCRP, respectively. In a first run, the effect of compounds **5**, **6**, **10**, **11**, and **16** (Figure 2) at 100 μm final concentration was tested (Figure 3). All compounds showing an inhibitory effect of more than 50 % compared with full inhibition by the known inhibitors of P‐gp (verapamil) and BCRP (Ko143) were further processed, and IC_50_ values were determined (IC_50_ curves and Hill coefficients are available in Figure S1 and Table S3 in the Supporting Information). In order to allow comparison with the in silico classification models, compounds with IC_50_ values below 10 μm were considered as active. As shown in Table 2, eight compounds were identified as inhibitors for BCRP (**5 b, 6 a, 6 b, 10 a**, **10 b**, **11 b**, **16 b**, **16 c**), while one compound (**5 a**) showed borderline activity on BCRP with an IC_50_ value of 16.13±3 μm. For P‐gp, nine compounds were classified as inhibitors (**5 a**, **5 b**, **6 a**, **10 a**, **10 b**, **11 b**, **16 a**, **16 b**, **16 c**). In addition, compounds **6 b** and **11 a** showed borderline inhibitory activity on P‐gp with IC_50_ values of 11±1 and 13.9±1.5 μm, respectively.

**Table 2:** Results of in silico prediction and in vitro IC_50_ evaluation of BCRP and P‐gp inhibition for the synthesized compounds (Figure 2).

Compd	log *P* ^[a]^	Substituents		BCRP		P‐gp	
		R^1^	R^2^	in silico^[b]^	IC_50_ [μm]^[c]^	in silico^[b]^	IC_50_ [μm]^[c]^
**5 a**	2.37	Bn	H	0.25	16.1±3.0	**0.97**	2.1±0.4
**5 b**	2.36	Bn	OMe	0.37	3.1±0.5	**0.92**	2.2±0.6
**5 c**	0.53	H	H	0.08	n.d.	0.46	58.7±9.2
**6 a**	1.93	Bn	H	0.39	5.8±1.0	**0.98**	1.6±0.6
**6 b**	1.92	Bn	OMe	**0.64**	3.8±0.6	**0.96**	11.0±1.0
**6 c**	0.09	H	H	0.15	n.d.	**0.78**	n.d.
**10 a**	2.94	Bn	H	0.17	3.3±0.6	**0.88**	1.3±0.1
**10 b**	2.93	Bn	OMe	0.39	2.9±0.5	**0.83**	1.0±0.2
**10 c**	1.10	H	H	0.07	n.d.	0.19	34.8±1.8
**11 a**	2.50	Bn	H	0.40	21.5±2.9	**0.94**	13.9±1.5
**11 b**	2.49	Bn	OMe	**0.75**	2.3±0.0	**0.91**	6.2±0.4
**11 c**	0.66	H	H	0.19	n.d.	0.42	n.d.
**16 a**	2.86	CH_3_	–	0.22	n.d.	**0.97**	0.3±0.0
**16 b**	3.08	CH_2_F	–	0.19	5.5±0.9	**0.97**	0.5±0.1
**16 c**	3.47	CH_3_F	–	0.24	4.9±1.1	**0.97**	0.9±0.2

[a] Calculated partition coefficient log *P*_octanol/water_ determined using Molecular Operating Environment (MOE) version 2014.09. [b] Score between 0 and 1, given by in silico models, roughly corresponds to the probability of being an inhibitor; in silico predictions over 0.5 are marked in bold and represent compounds that are predicted as inhibitors by the models. [c] IC_50_ values represent the mean±SD of at least *n*=3 independent experiments performed at least in duplicate; for inhibition of BCRP, **11 a** was measured in *n*=2 independent experiments; the exact number of experiments is given in the Supporting Information (Figure S1 and Table S3); IC_50_ values were evaluated as described in the Experimental Section; n.d.: not determined.

Comparing the inhibitory activity on P‐gp and BCRP, seven compounds (**5 a**, **6 a**, **10 a**, **10 b**, **16 a**, **16 b**, **16 c**) were more effective on P‐gp than on BCRP (Table 2). The most pronounced difference in the inhibitory activity on P‐gp and BCRP was observed for the fluorinated compounds **16 a**, **16 b**, and **16 c** and the aryloxyethanolamine **5 a** giving more than fivefold higher IC_50_ values for BCRP than for P‐gp. Another three compounds, namely **6 a**, **10 a**, and **10 b** were more than 2.6‐fold better inhibitors for P‐gp than for BCRP. Compounds **5 b** and **11 a** showed comparable inhibitory activity on both ABC proteins (1.4‐ and 1.5‐fold higher IC_50_ values for BCRP). The methoxy‐substituted compounds **6 b** and **11 b** bearing a piperazinedione moiety were the only ones showing a higher inhibitory activity on BCRP than on P‐gp, with **2.9**‐ and **2.7**‐fold lower IC_50_ values for BCRP, respectively. Finally, **5 c, 6 c**, **10 c**, and **11 c** were identified as noninhibitors for both ABC proteins, as their IC_50_ values were either far above 10 µm (**5 c** and **10 c** on P‐gp) or did not show more than 50% inhibition at 100 µm.

In addition, compound **11 a** was shown to be a weak inhibitor for BCRP (IC_50_: 21.5±2.9 μm) and a borderline inhibitor for P‐gp (IC_50_: 13.9±1.5 μm).

### Structure–activity relationships (SAR)

In order to gain insights into molecular features that might trigger P‐gp vs. BCRP selectivity of propafenone‐type inhibitors, we modified the substituent at the nitrogen atom towards more rigid and less basic properties. These modifications were complemented by a set of distinct variations, which should allow assessing if the compounds still show the basic SAR pattern reported for propafenones. Specifically, these include the linker region (propanol vs. ethylene), the phenone moiety (phenylpropiophenone vs. propiophenone), as well as the central aromatic ring (4‐methoxy vs. 4‐H). In addition, we calculated the logP values of all compounds and put them in relation to previously published logP/pIC_50_ correlations for this scaffold.

For P‐glycoprotein, the SAR pattern retrieved is mainly in line with those reported for other propafenone analogues: i) there is a significant correlation of pIC_50_ values with the logP of the compounds (r^2^=0.71),^21^ ii) elimination of the phenyl ring of the phenylpropiophenone moiety strongly decreases biological activity (which is most probably related to the logP decrease), iii) shortening the distance between the central aromatic ring and the nitrogen atom slightly decreases biological activity,^22^ and iv) increasing electron density of the central aromatic ring slightly increases activity.^23^ However, the ethylene compound **6 a** with a diketopiperazine moiety at the nitrogen atom does not follow this pattern. It is tenfold more active than its propanol analogue **11 a**, and is also more active than its methoxy analogue **6 b** by a factor of 7

For BCRP, the picture is more complex, and there are no obvious trends. Also with respect to lipophilicity of the compounds, there is no correlation with pIC_50_ values (r^2^=0.02). Interestingly, the most active compound (**11 b**) belongs to the diketopiperazine series, whereas in case of P‐gp it is a proline analogue (**10 b**).

### Prospective performance of the in silico models

The BCRP inhibition model tends to give low scores in our chemical series, that is, rarely predicts a compound as an inhibitor. In our specific case, 6 compounds out of 15 are mispredicted as inactives (**5 b**, **6 a**, **10 a**, **b**, **16 b**, **c**), which results in an accuracy of 60 %. The P‐gp inhibition model, on the contrary, tends to give high scores, that is, it often predicts a compound as an inhibitor. In our specific case, the model makes one clear mistake (predicting the des‐phenyl analogue **6 c** as active) and 2 misclassifications of borderline compounds (predicting **6 b** (IC_50_=11.0±1 μm) and **11 a** (IC_50_=13.9±1.5 μm) as inhibitors when they show activity slightly above 10 μm, the IC_50_ threshold applied for categorizing the compounds).

To understand this behavior of both our models, for each compound of our series we retrieved the five nearest structural neighbors of the training sets (see Supporting Information for Tables S1 and S2 showing the average similarity values). In the BCRP training set, all neighbors retrieved were propafenone derivatives from the study of Cramer et al.,^13^ and all these compounds were inactives for BCRP. This explains the tendency of the model to predict most of the propafenone derivatives as noninhibitors of BCRP. In contrast, in the P‐gp training set, all retrieved neighbors were propafenone derivatives annotated as inhibitors of P‐gp. This explains the tendency of the P‐gp model to predict most propafenone derivatives as active.

The difference between the prospective results and the predicted cross‐validation results seems striking, but is inherently linked to the composition of the training set. Indeed, the in silico models are applied on structurally very similar compounds. As a result, the fingerprint features encoding these structures are very similar to each other, and the results given by the models cannot show a high variability. The cross‐validation rather evaluates the generalizability of the model than its specific capability to properly predict SAR subtleties within the propafenone family. However, if one directly uses the score to rank the compounds according to their probability to inhibit P‐gp or BCRP, instead of applying a fixed threshold, the calculated prospective AUCs would be 0.78 for BCRP and 0.85 for P‐gp. Thus, these models are still valuable tools for prioritizing propafenone‐type compounds in medicinal chemistry projects.

### Selectivity vs. multitarget inhibition

Although the activity differences for compounds **5**–**11** between P‐gp and BCRP are less than a factor of 10 [selectivity indices (SI) P‐gp/BCRP=0.13–2.82], some trends can be deduced from the data. Highest selectivity for P‐gp was obtained for the two ethylene analogues **5 a** (SI=0.13) and **6 a** (SI=0.27), both being unsubstituted at the central aromatic ring and having either a proline or a diketopiperazine in the vicinity of the nitrogen atom. In case of BCRP selectivity, the two diketopiperazines **6 b** and **11 b** showed the highest SI values (2.82 and 2.70, respectively). Both of them show a methoxy moiety at position 4 of the central aromatic ring and belong either to the ethylene (**6 b**) or the propanolamine (**11 b**) class of compounds. The remaining compounds may be regarded as dual transporter inhibitors, showing SI values in the range of 0.34–0.71 and pIC_50_ values in the low micromolar range. Selectivity towards BCRP thus seems to be triggered mainly by the substituent on the central aromatic ring, as well as the diketopiperazine moiety. This might at least in part be due to the basic feature of the nitrogen atom, as exemplified by the **16 a–c** series of compounds. By introduction of fluorine atoms, the basicity of the nitrogen atom is systematically varied. Although in all cases the compounds are more active with respect to P‐gp than to BCRP, there is a clear tendency in P‐gp that lower basicity/H‐bond donor properties lead to lower activity. This is not seen in BCRP, which is in line with previous observations for propafenones.^13^, ^24^ Furthermore, the switch from proline to diketopiperazine also decreases flexibility in the vicinity of the nitrogen atom. It is tempting to speculate that a further morphing towards a more fumitremorgin C like scaffold would increase selectivity.

## Conclusions

The ABC‐transporters P‐glycoprotein (P‐gp) and the breast cancer resistance protein (BCRP) are characterized by a broad and partly overlapping ligand profile. Due to their importance for absorption, distribution, metabolism, excretion (ADME), and toxicity, design of multitransporter inhibitors as well as transporter‐specific compounds would be a valuable effort for obtaining a set of tool compounds. Building on previous work, we extended our studies on the propafenone scaffold as a versatile chemotype for triggering transporter selectivity and for increasing our understanding of the molecular features driving ligand–transporter interaction. Results indicate that both the flexibility of the substituent at the nitrogen atom, as well as the basicity of the nitrogen atom, triggers transporter selectivity. Furthermore, inhibitory activity of compounds for P‐gp seems to be much more influenced by logP than that for BCRP. Exploiting these differences should thus further allow to dissect the molecular features triggering ligand selectivity for the two polyspecific ABC‐transporters: P‐gp and BCRP.

## Experimental Section

### Chemistry

**General**. All moisture‐sensitive reactions were conducted in anhydrous solvents (Sigma–Aldrich) under dry argon atmosphere. All solvents and reagents were obtained from Sigma–Aldrich, ABCR, TCI, or Acros and used without further purification. Reactions were monitored by analytical thin‐layer chromatography (TLC) using precoated TLC sheets ALUGRAM Xtra SIL G/UV254 (Macherey–Nagel, 0.20 mm silica gel 60 layer with fluorescent indicator UV254). Visualization was carried out by UV light (254 nm), ninhydrin, and/or Seebach stain. Flash chromatography was performed using Merck silica gel 60 m (0.040–0.063 mm). Yields are not optimized.

**NMR spectroscopy**. NMR spectra were registered either on a Bruker Avance 200 or Ultrashield 500 spectrometer (Billerica, MA, USA) at 200 or 500 MHz, using CDCl_3_ or D_6_]DMSO as solvents. Chemical shifts (*δ*) are expressed in parts per million (ppm) relative to tetramethylsilane (TMS), and coupling constants (*J*) are given in Hertz (Hz). Multiplicities are described as s (singlet), brs (broad singlet), d (doublet), t (triplet), q (quadruplet), quin (quintuplet), septet, or m (multiplet). ^1^H and ^13^C spectra of tested compounds are given in the Supporting Information. Purity of target compounds was defined by ^13^C NMR (absence of additional signals).

**High‐resolution electrospray ionization mass spectrometry (HR‐ESI‐MS)**. HR‐ESI‐MS spectra were obtained on a maXis HD ESI‐Qq‐TOF mass spectrometer (Bruker Daltonics, Bremen, Germany) using direct infusion. The ESI ion source was operated as follows: capillary voltage: 1.0 to 4.0 kV (individually optimized), nebulizer: 0.4 bar (N2), dry gas flow: 4 L min^−1^ (N2), and dry temperature: 200 °C. Mass spectra were recorded in the range of *m*/*z* 50–1550 in the positive‐ion mode. The sum formulas were determined using Bruker Compass Data Analysis 4.2 based on the mass accuracy (Δ*m*/*z*≤2 ppm) and isotopic pattern matching (SmartFormula algorithm).

**2‐(2‐Phenethyl‐1,3‐dioxolan‐2‐yl)phenol (1 a)**: 2′‐Hydroxy‐3‐phenylpropiophenone (1 g, 1 eq, 4.4 mmol), ethylene glycol (0.37 mL, 1.5 eq, 6.6 mmol), trimethylorthoformate (0.73 mL, 1.5 eq, 6.6 mmol), and trifluoromethanesulfonic acid (3.9 μL, 0.01 eq, 0.04 mmol) in hexane (7 mL) were heated at 42 °C o/n, then ethylene glycol (0.2 mL) and trimethylorthoformate (0.4 mL) were added, and the reaction was heated for two more days. Then the mixture was cooled to RT, and Et_3_N (3 mL) was added followed by saturated aq NaHCO_3_ (25 mL). This was extracted with Et_2_O (4×50 mL, a few drops Et_3_N added), and the combined organic extracts were washed once with brine (30 mL), dried over Na_2_SO_4_, and concentrated to afford **1 a** as a white solid (99 %, 1.17 g), which was used without further purification. NMR data were in good accordance with the literature.^25^

**5‐Methoxy‐2‐(2‐phenethyl‐1,3‐dioxolan‐2‐yl)phenol (1 b)**: Preparation like **1 a**; used without further purification. Brown oil (4.54 g, 97 %); ^1^H NMR (500 MHz, CDCl_3_): *δ*=2.20–2.28 (m, 2 H), 2.70–2.79 (m, 2 H), 3.79 (s, 3 H), 3.94 (brs, 2 H), 4.13 (brs, 2 H), 6.41–6.51 (m, 2 H), 7.13–7.23 (m, 4 H), 7.23–7.36 (m, 2 H), 8.37 ppm (s, 1 H); ^13^C NMR (125 MHz, CDCl_3_): *δ*=29.5, 41.4, 53.7, 64.5 (2C), 101.9, 106.3, 111.3, 117.5, 125.8, 127.8, 128.3 (2C), 128.3 (2C), 141.5, 155.9, 161.6 ppm.

**2‐(2‐Methyl‐1,3‐dioxolan‐2‐yl)phenol (1 c)**: Preparation like **1 a**. Purification by column chromatography (petrol ether:EtOAc, 20:1→15:1) yielded **1 c** as a yellow oil (9.99 g, 63 %). NMR data were in good accordance with the literature.^16^

**2‐(2‐(Oxiran‐2‐ylmethoxy)phenyl)‐2‐phenethyl‐1,3‐dioxolane (2 a)**: The ketal **1 a** (5 g, 1 eq, 18.5 mmol) was dissolved in anhydrous dimethylformamide (40 mL) and heated to 70 °C, then NaH (60 % dispersion, 1.11 g, 1.5 eq, 27.7 mmol) was added in portions during 30 min. After stirring for 1 h at this temperature, epichlorohydrine (4.4 mL, 3 eq, 55.5 mmol) was added and heated overnight. TLC showed no starting material; therefore, the reaction mixture was concentrated, diluted with Et_2_O (100 mL) and extracted once with sat. NaHCO_3_ solution (100 mL) and water (100 mL). The combined aqueous extracts were extracted once with Et_2_O (100 mL), and the combined organic phases were dried over Na_2_SO_4_, and the solvent was evaporated. The crude product was triturated with petrol ether (20 mL) under cooling, and the petrol ether phase was removed, which yielded the product as a yellowish oil (6.0 g, 99 %) after drying. NMR data were in good accordance with the literature.^25^

**2‐(4‐Methoxy‐2‐(oxiran‐2‐ylmethoxy)phenyl)‐2‐phenethyl‐1,3‐dioxolane (2 b)**: Preparation like **2 a**; purification by column chromatography (petrol ether:EtOAc, 4:1 to 2:1) afforded a colorless oil (3.52 g, 74 %); ^1^H NMR (500 MHz, CDCl_3_): *δ*=2.39–2.49 (m, 2 H), 2.59–2.67 (m, 2 H), 2.87 (d, *J*=3.5 Hz, 2 H), 3.35–3.39 (m, 1 H), 3.80 (s, 3 H), 3.85–3.92 (m, 2 H), 4.02–4.08 (m, 3 H), 4.24 (dd, *J*=11.2, 3.0 Hz, 1 H), 6.47 (dd, *J*=8.4, 2.4 Hz, 1 H), 6.49 (d, *J*=2.2 Hz, 1 H), 7.13 (t, *J*=7.1 Hz, 1 H), 7.17 (d, *J*=7.3 Hz, 2 H), 7.23 (d, *J*=7.6 Hz, 2 H), 7.24–7.29 (m, 1 H), 7.42 ppm (d, *J*=8.5 Hz, 1 H); ^13^C NMR (125 MHz, CDCl_3_): *δ*=30.2, 39.7, 44.7, 50.2, 55.3, 64.70, 64.72, 68.8, 101.1, 104.2, 109.9, 122.5, 125.4, 128.1 (2C), 128.30, 128.34 (2C), 128.36, 142.4, 157.0, 160.7 ppm; HRMS (ESI): calcd for C_21_H_24_NaO_5_ *m*/*z* 357.1697 [*M*+Na]^+^, found *m*/*z* 357.1697 [*M*+Na]^+^.

**2‐Methyl‐2‐(2‐(oxiran‐2‐ylmethoxy)phenyl)‐1,3‐dioxolane (2 c)**: Preparation like **2 a**.The crude product was purified by column chromatography (petrol ether: EtOAc 6:1 to 2:1) to yield **2 c** as a colorless oil (3.26 g, 84 %);^1^H NMR (500 MHz, CDCl_3_): *δ*=1.79 (s, 3 H), 2.87–2.92 (m, 2 H), 3.38–3.43 (m, 1 H), 3.79–3.88 (m, 2 H), 4.01–4.06 (m, 2 H), 4.08 (dd, *J*=11.2, 4.9 Hz, 1 H), 4.31 (dd, *J*=11.2, 3.0 Hz, 1 H), 6.92 (d, *J*=8.2 Hz, 1 H), 6.94 (td, *J*=7.4, 1.1 Hz, 1 H), 7.26 (td, *J*=7.4, 1.1 Hz, 1 H), 7.50 ppm (dd, *J*=7.7, 1.7 Hz, 1 H); ^13^C NMR (125 MHz, CDCl_3_): *δ*=25.4, 44.7, 50.3, 64.5 (2C), 68.9, 108.5, 113.6, 120.8, 126.8, 129.4, 131.1, 156.0 ppm; HRMS (ESI): calcd for C_13_H_16_NaO_4_ *m*/*z* 259.0941 [*M*+Na]^+^, found *m*/*z* 259.0945 [*M*+Na]^+^.

**1‐Amino‐3‐(2‐(2‐phenethyl‐1,3‐dioxolan‐2‐yl)phenoxy)propan‐2‐ol (3 a)**: The epoxide **2 a** (2 g, 1 eq, 6.12 mmol), was dissolved in MeOH (23 mL) and NH_3_ in MeOH (7 m, 23 mL) was added. It was stirred for 2 d at RT and then evaporated to yield a crude yellow oil (2.28 g, 109 %), used without further purification; ^1^H NMR (500 MHz, CDCl_3_): *δ*=2.44 (t, *J*=8.5 Hz, 2 H), 2.55–2.73 (m, 2 H), 2.80–2.99 (m, 2 H), 3.80–3.93 (m, 2 H), 3.93–4.14 (m, 4 H), 4.17–4.25 (m, 1 H), 6.91 (d, *J*=6.8 Hz, 1 H), 6.97 (t, *J*=6.8 Hz, 1 H), 7.09–7.18 (m, 1 H), 7.15 (d, *J*=7.0 Hz, 2 H), 7.23 (d, *J*=6.3 Hz, 2 H), 7.26–7.33 (m, 1 H), 7.48 ppm (d, *J*=7.3 Hz, 1 H); ^13^C NMR (125 MHz, CDCl_3_): *δ*=30.0, 40.0, 44.2, 64.7, 64.8, 70.8, 72.4, 110.3, 114.4, 120.0, 125.6, 127.1, 128.25 (2C), 128.30 (2C), 129.6, 130.0, 142.1, 156.6 ppm; HRMS (ESI): calcd for C_20_H_26_NO_4_ *m*/*z* 344.1856 [*M*+H]^+^, found *m*/*z* 344.1857 [*M*+H]^+^.

**1‐Amino‐3‐(5‐methoxy‐2‐(2‐phenethyl‐1,3‐dioxolan‐2‐yl)phenoxy)‐propan‐2‐ol (3 b)**: Prepared like **3 a**; yellow oil (1.70 g, 54 %); ^1^H NMR (500 MHz, CDCl_3_): *δ*=2.40 (t, *J*=8.7 Hz, 2 H), 2.54–2.70(m, 2 H), 2.83–2.97 (m, 2 H), 3.79 (s, 3 H), 3.82–3.90 (m, 2 H), 3.92 (d, *J*=6.3 Hz, 1 H), 3.91–3.99 (m,1 H), 4.00–4.10 (m, 2 H), 4.17 (d, *J*=6.9 Hz, 1 H), 6.44–6.52 (m, 2 H), 7.09–7.18 (m, 3 H), 7.20–7.26 (m, 2 H), 7.37 ppm (d, *J*=8.5 Hz, 1 H); ^13^C NMR (125 MHz, CDCl_3_): *δ*=30.1, 40.2, 44.2, 55.4, 64.6, 64.7, 70.7, 72.4, 101.7, 105.1, 110.3, 122.5, 125.6, 127.9, 128.2 (2C), 128.3 (2C), 142.0, 157.5, 160.9 ppm; HRMS (ESI): calcd for C_21_H_28_NO_5_ *m*/*z* 374.1962 [*M*+H]^+^, found *m*/*z* 374.1964 [*M*+H]^+^.

**1‐Amino‐3‐(2‐(2‐methyl‐1,3‐dioxolan‐2‐yl)phenoxy)propan‐2‐ol (3 c)**: Preparation like **3 a**; the crude product (mixture with dimer) was purified by column chromatography (CH_2_Cl_2_+1 % Et_3_N to CH_2_Cl_2_:MeOH=20:1+1 % Et_3_N) to yield the pure product as a yellowish solid (378 mg, 14 %) and the rest (1.61 g) as a mixture with the dimer; ^1^H NMR (500 MHz, CDCl_3_): *δ*=1.77 (s, 3 H), 2.82–2.92 (m, 2 H), 3.77–3.88 (m, 2 H), 3.92–3.99 (m, 2 H), 4.01–4.08 (m, 2 H), 4–17–4.26 (m, 1 H), 6.91 (d, *J*=8.2 Hz, 1 H), 6.94 (td, *J*=7.6, 0.9 Hz, 1 H), 7.23–7.29 (m, 1 H), 7.46 ppm (dd, *J*=7.7, 1.7 Hz, 1 H); ^13^C NMR (125 MHz, CDCl_3_): *δ*=25.8, 44.3, 64.4, 64.5, 71.1, 72.3, 108.8, 114.4, 120.9, 126.3, 129.5, 130.9, 156.5 ppm; HRMS (ESI): calcd for C_13_H_20_NO_4_ *m*/*z* 254.1387 [*M*+H]^+^, found *m*/*z* 254.1390 [*M*+H]^+^.

**2‐(2‐(3‐Phenylpropanoyl)phenoxy)acetonitrile (7 a)**: 2′‐Hydroxy‐3‐phenylpropiophenone (4 g, 1 eq, 17.7 mmol) was dissolved in acetone (200 mL), and K_2_CO_3_ (9.77 g, 4 eq, 70.7 mmol) was added. Bromoacetonitrile (2.54 g, 1.2 eq, 21.2 mmol) dissolved in acetone (100 mL) was added dropwise throughout 30 min. The reaction was stirred at 50 °C for 4 d, then it was filtered, washed once with acetone, and concentrated. The residue was dissolved in CH_2_Cl_2_ (100 mL) and water (100 mL), and the aqueous phase was extracted a CH_2_Cl_2_/CHCl_3_ mixture (10 % CHCl_3_, 3×100 mL). The combined organic extracts were washed once with brine (70 mL), dried over Na_2_SO_4_, and the solvent was evaporated.to yield a brown solid (4.15 g, 88 %). NMR data were in good accordance with the literature.^18^

**2‐(5‐Methoxy‐2‐(3‐phenylpropanoyl)phenoxy)acetonitrile (7 b)**: Preparation like **7 a**; brown solid (4.86 g, 93 %);^1^H NMR (500 MHz, CDCl_3_): *δ*=3.02 (t, *J*=7.6 Hz, 2 H), 3.24 (t, *J*=7.6 Hz, 2 H), 3.87 (s, 3 H), 4.80 (s, 2 H), 6.52 (d, *J*=2.2 Hz, 1 H), 6.66 (dd, *J*=8.7, 2.0 Hz, 1 H), 7.17–7.22 (m, 1 H), 7.22–7.25 (m, 2 H), 7.27–7.32 (m, 2 H), 7.81 ppm (d, *J*=8.8 Hz, 1 H); ^13^C NMR (125 MHz, CDCl_3_): *δ*=30.4, 44.9, 54.0, 55.7, 100.4, 107.5, 114.5, 121.8, 126.0, 128.4 (4C), 133.1, 141.4, 157.0, 164.1, 198.5 ppm; HRMS (ESI): calcd for C_18_H_17_NNaO_3_ *m*/*z* 318.1101 [*M*+Na]^+^, found *m*/*z* 318.1100 [*M*+Na]^+^.

**2‐(2‐Acetylphenoxy)acetonitrile (7 c)**: Preparation like **7 a**. The product was a white solid (3.54 g, 100 %), which was used without further purification. NMR data were in good accordance with the literature.^26^

**2‐(2‐(2‐Phenethyl‐1,3‐dioxolan‐2‐yl)phenoxy)acetonitrile (8 a)**: The nitrile **7 a** (4 g, 1 eq, 15.1 mmol), ethylene glycol (1.7 mL, 2 eq, 30.9 mmol), and *p*‐toluenesulfonic acid monohydrate (0.29 g, 0.1 eq, 1.5 mmol) were dissolved/suspended in toluene (50 mL) and heated o/n in a Dean–Stark apparatus. Then the reaction mixture was poured into ice‐cold sat. NaHCO_3_ solution (50 mL) and extracted with EtOAc (4×70 mL). The combined organic extracts were dried over Na_2_SO_4,_ filtered, and evaporated to yield a brown oil (4.52 g, 97 %); ^1^H NMR (500 MHz, CDCl_3_): *δ*=2.35–2.44 (m, 2 H), 2.60–2.69 (m, 2 H), 3.84–3.93 (m, 2 H), 4.04–4.13 (m, 2 H), 4.81 (s, 2 H), 7.07 (d, *J*=8.2 Hz, 1 H), 7.09–7.18 (m, 4 H), 7.20–7.26 (m, 2 H), 7.35 (td, *J*=7.7, 1.7 Hz, 1 H), 7.58 ppm (dd, *J*=7.7, 1.7 Hz, 1 H); ^13^C NMR (125 MHz, CDCl_3_): *δ*=29.9, 40.0, 55.2, 64.9 (2C), 109.5, 115.5, 115.7, 123.3,125.6, 128.1, 128.2 (2C), 128.3 (2C), 129.8, 132.0, 142.0, 154.4 ppm; HRMS (ESI): calcd for C_19_H_19_NNaO_3_ *m*/*z* 332.1257 [*M*+Na]^+^, found *m*/*z* 332.1258 [*M*+Na]^+^.

**2‐(5‐Methoxy‐2‐(2‐phenethyl‐1,3‐dioxolan‐2‐yl)phenoxy)acetonitrile (8 b)**: Preparation like **8 a**. The crude product was purified by column chromatography (petrol ether:EtOAc 4:1 to 2:1) to yield **8 b** as a yellow oil (3.83 g, 72 %); ^1^H NMR (500 MHz, CDCl_3_): *δ*=2.34–2.41 (m, 2 H), 2.59–2.66 (m, 2 H), 3.82 (s, 3 H), 3.85–3.91 (m, 2 H), 4.05–4.09 (m, 2 H), 4.79 (s, 2 H), 6.61 (s, 1 H), 6.62 (dd, *J*=6.5, 2.4 Hz, 1 H), 7.12–7.17 (m, 3 H), 7.21–7.26 (m, 2 H), 7.48 ppm (dd, *J*=6.3, 3.2 Hz, 1 H); ^13^C NMR (125 MHz, CDCl_3_): *δ*=30.1, 40.2, 55.0, 55.5, 64.7 (2C), 102.9, 107.1, 125.6, 128.2 (2C), 128.3 (2C), 128.9, 109.5, 115.3, 124.0, 142.1, 155.1, 160.7 ppm; HRMS (ESI): calcd for C_20_H_21_NNaO_4_ *m*/*z* 362.1363 [*M*+Na]^+^, found *m*/*z* 362.1366 [*M*+Na]^+^.

**2‐(2‐(2‐Methyl‐1,3‐dioxolan‐2‐yl)phenoxy)acetonitrile (8 c)**: Preparation like **8 a**. The crude product was purified by column chromatography (petrol ether:EtOAc 4:1 to 1:1) to yield **8 c** as a white solid (3.01 g, 81 %);^1^H NMR (500 MHz, CDCl_3_): *δ*=1.75 (s, 3 H), 3.82–3.86 (m, 2 H), 4.04–4.08 (m, 2 H), 4.85 (s, 2 H), 7.08 (d, *J*=8.2 Hz, 1 H), 7.10 (t, *J*=7.4 Hz, 1 H), 7.34 (td, *J*=7.8, 1.7 Hz, 1 H), 7.56 ppm (dd, *J*=7.6, 1.6 Hz, 1 H); ^13^C NMR (125 MHz, CDCl_3_): *δ*=25.8, 64.6 (2C), 55.1, 108.0, 114.5, 114.6, 123.4, 127.3, 129.7, 132.8, 154.3 ppm; HRMS (ESI): calcd for C_12_H_13_NNaO_3_ *m*/*z* 242.0788 [*M*+Na]^+^, found *m*/*z* 242.0789 [*M*+Na]^+^.

**2‐(2‐(2‐Phenethyl‐1,3‐dioxolan‐2‐yl)phenoxy)ethan‐1‐amine (9 a)**: Red‐Al (∼3.5 m in toluene, 4.5 mL, 9.7 eq, 15.8 mmol) was placed in a flask, and the nitrile **8 a** (0.5 g, 1 eq, 1.62 mmol) dissolved in toluene (20 mL) was added dropwise during 30 min. The reaction mixture was stirred at RT for 3 h, and then water (20 mL) and Na‐K‐tartrate (13 g) was added and stirred for 30 min at RT. The mixture was filtered over celite and washed with EtOAc. Then the phases were separated, and the aqueous phase was extracted with EtOAc (3×80 mL). The combined organic extracts were washed once with brine (100 mL), dried over Na_2_SO_4_, and evaporated. The crude product was purified by column chromatography (CH_2_Cl_2_+1 % Et_3_N to CH_2_Cl_2_:MeOH=20:1+1 % Et_3_N) to yield **9 a** as a colorless oil (76 mg, 15 %);^1^H NMR (500 MHz, CDCl_3_): *δ*=2.43–2.50 (m, 2 H), 2.61–2.69 (m, 2 H), 3.08 (brs, 2 H), 3.82–3.92 (m, 2 H), 3.99–4.10 (m, 4 H), 6.89 (d, *J*=8.2 Hz, 1 H), 6.94 (t, *J*=7.4 Hz, 1 H), 7.11–7.18 (m, 3 H), 7.20–7.33 (m, 3 H), 7.51 ppm (dd, *J*=7.6, 1.6 Hz, 1 H); ^13^C NMR (125 MHz, CDCl_3_): *δ*=30.1, 39.7, 41.7, 64.7 (2C), 70.7, 110.0, 113.0, 120.2, 125.5, 127.4, 128.2 (2C), 128.3 (2C), 129.4, 129.7, 142.2, 156.5 ppm; HRMS (ESI): calcd for C_19_H_24_NO_3_ *m*/*z* 314.1751 [*M*+H]^+^, found *m*/*z* 314.1752 [*M*+H]^+^.

**2‐(5‐Methoxy‐2‐(2‐phenethyl‐1,3‐dioxolan‐2‐yl)phenoxy)ethan‐1‐amine (9 b)**: LiAlH_4_ (671 mg, 2 eq, 17.7 mmol) was suspended in absolute Et_2_O (200 mL), and the nitrile **8 b** (3 g, 1 eq, 8.8 mmol, dissolved in 50 mL Et_2_O) was added carefully throughout 30 min. After completion of the addition, the reaction mixture was heated at reflux for 4 h and afterwards stirred at RT o/n. First EtOAc, and then saturated Na‐K‐tartrate solution, was added carefully with cooling, and the mixture was stirred for 3 h. Then the aqueous phase was extracted with EtOAc (3×150 mL). The organic phases were dried over Na_2_SO_4_, filtered, and evaporated. The crude product was purified by column chromatography (CH_2_Cl_2_ to CH_2_Cl_2_:MeOH 20:1, always 1 % conc. aq NH_4_OH added) to yield a yellow oil (1.27 g, 42 %); ^1^H NMR (500 MHz, CDCl_3_): *δ*=2.40–2.46 (m, 2 H), 2.59–2.66 (m, 2 H), 3.07 (t, *J*=4.9 Hz, 2 H), 3.80 (s, 3 H), 3.83–3.88 (m, 2 H), 4.01 (t, *J*=4.9 Hz, 2 H), 4.02–4.06 (m, 2 H), 6.43–6.47 (m, 2 H), 7.11–7.17 (m, 3 H), 7.20–7.26 (m, 2 H), 7.40 ppm (d, *J*=8.8 Hz, 1 H); ^13^C NMR (125 MHz, CDCl_3_): *δ*=30.2, 39.9, 41.6, 55.3, 64.6 (2C), 70.5, 100.7, 103.6, 110.0, 122.2, 125.5, 128.2 (3C), 128.3 (2C), 142.5, 157.4, 160.7 ppm; HRMS (ESI): calcd for C_20_H_26_NO_4_ *m*/*z* 344.1856 [*M*+H]^+^, found *m*/*z* 344.1860 [*M*+H]^+^.

**2‐(2‐(2‐Methyl‐1,3‐dioxolan‐2‐yl)phenoxy)ethan‐1‐amine (9 c)**: Preparation like **9 b**. The crude product was purified by column chromatography (CH_2_Cl_2_ with 1 % Et_3_N to CH_2_Cl_2_:MeOH=12:1+1 % Et_3_N) to yield a brown oil (744 mg, 39 %); ^1^H NMR (500 MHz, CDCl_3_): *δ*=1.78 (s, 3 H), 3.10 (t, *J*=5.0 Hz, 2 H), 3.77–3.85 (m, 2 H), 3.99–4.04 (m, 2 H), 4.05 (t, *J*=5.0 Hz, 2 H), 6.90 (d, *J*=8.2 Hz, 1 H), 6.91 (td, *J*=7.5, 1.2 Hz, 1 H), 7.25 (ddd, *J*=8.0, 7.4, 1.9 Hz, 1 H), 7.49 ppm (dd, *J*=7.6, 1.6 Hz, 1 H); ^13^C NMR (125 MHz, CDCl_3_): *δ*=25.6, 41.7, 64.4 (2C), 70.7, 108.5, 113.0, 120.2, 126.7, 129.3, 130.6, 156.4 ppm; HRMS (ESI): calcd for C_12_H_18_NO_3_ *m*/*z* 224.1281 [*M*+H]^+^, found *m*/*z* 224.1284 [*M*+H]^+^.

***N*** **‐(2‐hydroxy‐3‐(2‐(3‐phenylpropanoyl)phenoxy)propyl)pyrrolidine‐2‐carboxamide (5 a)**: *Coupling reaction*: The amine **4 a** (1000 mg, 1 eq, 2.91 mmol) and boc‐proline (627 mg, 1 eq, 2.91 mmol) were dissolved in anhydrous CH_2_Cl_2_ (15 mL), and dimethylaminopyridine (18 mg, 0.05 eq, 0.15 mmol) and dicyclohexylcarbodiimide (601 mg, 1 eq, 2.91 mmol) were added and stirred until TLC indicated completion (2 h to o/n). The reaction mixture was filtered, washed with sat. NaHCO_3_ solution (20 mL) and water (20 mL), and the aqueous extracts were extracted with CH_2_Cl_2_ (2×50 mL). The combined organic extracts were washed once with brine (50 mL), dried over Na_2_SO_4_, and the solvent was evaporated. The product was purified by column chromatography (CH_2_Cl_2_ to CH_2_Cl_2_:MeOH=30:1 to 15:1) to yield tert‐butyl 2‐((2‐hydroxy‐3‐(2‐(2‐phenethyl‐1,3‐dioxolan‐2‐yl)phenoxy)propyl)carbamoyl)pyrrolidine‐1‐carboxylate as a white solid (mixture of diastereomers, 986 mg, 63 %); ^1^H NMR (500 MHz, CDCl_3_): *δ*=1.41 (s, 9 H), 1.86 (brs, 2 H), 2.07–2.31 (m, 2 H), 2.42 (t, *J*=8.4 Hz, 2 H), 2.55–2.70 (m, 2 H), 3.34 (brs, 1 H), 3.39–3.51 (m, 2 H), 3.55 (brs, 1 H), 3.80–3.94 (m, 2 H), 4.00–4.12 (m, 2 H), 4.16–4.31 (m, 2 H), 6.90 (d, *J*=7.9 Hz, 1 H), 6.96 (t, *J*=7.9 Hz, 1 H), 7.11–7.17 (m, 3 H), 7.20–7.29 (m, 3 H), 7.47 ppm (d, *J*=7.9 Hz, 1 H); ^13^C NMR (125 MHz, CDCl_3_): *δ*=22.1/23.7, 28.2(3C), 30.0, 31.1, 40.0, 41.5, 47.0/47.1, 61.3, 64.7, 64.8, 69.0/69.3, 71.6/71.9, 79.9, 110.4, 114.6, 121.0/121.2, 125.6, 127.0, 128.3 (4C), 129.7, 130.0, 142.0, 156.5, 172.1 ppm; HRMS (ESI): calcd for C_30_H_40_N_2_NaO_7_ *m*/*z* 563.2728 [*M*+Na]^+^, found *m*/*z* 563.2733 [*M*+Na]^+^.

*Deprotection*: The protected derivative (500 mg, 1 eq, 0.92 mmol) was dissolved in dioxane (3 mL) and aq. HCl (4 m, 5 mL) was added and stirred at RT for 4 h. Then the reaction mixture was basified with aq NaOH solution, diluted with water, and extracted with EtOAc (4×50 mL), dried over Na_2_SO_4_; and the solvent was evaporated to yield a brown oil (380 mg, 98 %); ^1^H NMR (500 MHz, CDCl_3_): *δ*=1.63–1.73 (m, 2 H), 1.83–1.93 (m, 2 H), 2.04–2.19 (m, 1 H), 2.74–2.94 (m 2 H), 3.03 (t, *J*=7.6 Hz, 2 H), 3.28 (t, *J*=7.7 Hz, 2 H), 3.30–3.39 (m, 1 H), 3.46–3.55 (m, 1 H), 3.70–3.78 (m, 1 H), 3.94–4.08 (m, 3 H), 6.95 (d, *J*=8.2 Hz, 1 H), 7.02 (t, *J*=7.6 Hz, 1 H), 7.18 (t, *J*=7.3 Hz, 1 H), 7.22 (d, *J*=7.0 Hz, 2 H), 7.28 (d, *J*=8.2 Hz, 2 H), 7.43 (t, *J*=7.9 Hz, 1 H), 7.60 (d, *J*=7.6 Hz, 1 H), 8.02 ppm (brs, 1 H); ^13^C NMR (125 MHz, CDCl_3_): *δ*=26.1, 30.1, 30.7/30.8, 42.7/42.8, 44.7, 47.2, 60.3, 69.8, 70.5/70.6, 113.3/113.4, 121.1, 126.0, 128.3 (2C), 128.4 (2C), 129.9/130.0, 133.4, 141.3, 157.3, 177.3, 201.6 ppm; HRMS (ESI): calcd for C_23_H_29_N_2_NaO_4_ *m*/*z* 419.1941 [*M*+Na]^+^, found *m*/*z* 419.1939 [*M*+Na]^+^.

*Hydrochloride formation*: The amine (320 mg, 0.8 mmol) was dissolved in 3 mL methyl *tert*‐butyl ether (MTBE) and CH_3_CN (1 mL) and treated with 6 n HCl (1 mL), stirred for 1 h, and then the solvent was evaporated to yield the hydrochloride as a yellow semisolid (347 mg, 87 %)’; HRMS (ESI): calcd for C_23_H_29_N_2_NaO_4_ *m*/*z* 419.1941 [*M*+Na]^+^, found *m*/*z* 419.1939 [*M*+Na]^+^.

**N‐(2‐hydroxy‐3‐(5‐methoxy‐2‐(3‐phenylpropanoyl)phenoxy)propyl)‐pyrrolidine‐2‐carboxamide (5 b)**: Preparation of the protected derivative like **5 a**; purification by column chromatography (petrol ether:EtOAc=1:1 to pure EtOAc to yield tert‐butyl 2‐((2‐hydroxy‐3‐(5‐methoxy‐2‐(2‐phenethyl‐1,3‐dioxolan‐2‐yl)phenoxy)propyl)carbamoyl)pyrrolidine‐1‐carboxylate as a colorless honey (220 mg, 71 %); ^1^H NMR (500 MHz, CDCl_3_): *δ*=1.80–1.91 (m, 2 H), 1.32–1.47 (m, 9 H), 2.04–2.24 (m, 2 H), 2.38 (t, *J*=8.5 Hz, 2 H)„ 2.54–2.66 (m, 2 H), 3.21–3.58 (m, 4 H), 3.79 (s, 3 H), 3.81–3.91 (m, 3 H), 3.99–4.10 (m, 3 H), 4.11–4.19 (m, 1 H), 4.20–4.32 (m, 1 H), 6.40–6.52 (m, 2 H), 7.08–7.17 (m, 3 H), 7.19–7.25 (m, 2 H), 7.36 ppm (d, *J*=8.5 Hz, 1 H); ^13^C NMR (125 MHz, CDCl_3_): *δ*=24.6, 28.2/28.3 (3C), 30.1/30.2, 31.2, 40.2, 41.4/41.5, 47.1, 55.4, 61.3, 64.6, 64.7, 68.9, 71.9, 80.1/80.7, 101.7/101.8, 105.3, 110.6, 122.4, 125.6, 127.9, 128.2 (2C), 128.3 (2C), 142.1, 157.4, 160.9, 173.3 ppm; HRMS (ESI): calcd for C_31_H_42_N_2_NaO_8_ *m*/*z* 593.2833 [*M*+Na]^+^, found *m*/*z* 593.2838 [*M*+Na]^+^.

Deprotection like for **5 a**; orange honey (134 mg, 99 %); ^1^H NMR (500 MHz, CDCl_3_): *δ*=1.73 (quin, *J*=6.9 Hz, 2 H), 1.86–1.96 (m, 1 H), 2.12–2.22 (m, 1 H), 2.87–2.98 (m, 1 H), 2.98–3.06 (m, 1 H), 3.02 (t, *J*=7.6 Hz, 2 H), 3.25 (t, *J*=7.7 Hz, 2 H), 3.30–3.39 (m, 1 H), 3.49–3.61 (m, 1 H), 3.81–3.87 (m, 1 H), 3.84 (s, 3 H), 3.97–4.04 (m, 2 H), 4.04–4.11 (m, 1 H), 6.46 (d, *J*=1.9 Hz, 1 H), 6.53 (d, *J*=8.8 Hz, 1 H), 7.19 (t, *J*=7.3 Hz, 1 H), 7.23 (d, *J*=7.3 Hz, 2 H), 7.26–7.34 (m, 2 H), 7.74 (d, *J*=8.8 Hz, 1 H), 8.05 ppm (vrs, 1 H); ^13^C NMR (125 MHz, CDCl_3_): *δ*=26.1/26.2, 30.4, 30.8, 42.9, 44.5, 47.2/47.3, 55.6, 60.3, 69.8, 70.6/70.7, 99.9/100.0, 105.9/106.0, 121.0, 126.0, 128.3/128.4 (2C), 128.4/128.5 (2C), 132.5, 141.6, 159.8, 164.3, 177.4, 199.2 ppm; HRMS (ESI): calcd for C_24_H_30_N_2_NaO_5_ *m*/*z* 449.2047 [*M*+Na]^+^, found *m*/*z* 449.2050 [*M*+Na]^+^.

Hydrochloride formation like for **5 a** yielded a brown honey (108 mg, 98 %); HRMS (ESI): calcd for C_24_H_30_N_2_NaO_5_ *m*/*z* 449.2047 [M+Na]^+^, found *m*/*z* 449.2047 [M+Na]^+^.

***N*** **‐(3‐(2‐acetylphenoxy)‐2‐hydroxypropyl)pyrrolidine‐2‐carboxamide (5 c)**: Preparation of the protected derivative like for **5 a**. The product was purified by column chromatography (CH_2_Cl_2_ to CH_2_Cl_2_:MeOH=30:1) to yield tert‐butyl2‐((2‐hydroxy‐3‐(2‐(2‐methyl‐1,3‐dioxolan‐2‐yl)phenoxy)propyl)carbamoyl)pyrrolidine‐1‐carboxylate as a colorless semisolid (109 mg, 41 %); ^1^H NMR (500 MHz, CDCl_3_): *δ*=1.38 (brs, 6 H), 1.42 (brs, 3 H), 1.76 (s, 3 H), 1.79–1.90 (m, 2 H), 1.95–2.27 (m, 2 H), 3.22–3.41 (m, 1 H), 3.41–3.51 (m, 2 H), 3.52–3.73 (m, 1 H), 3.76–3.95 (m, 3 H), 4.00–4.12 (m, 3 H), 4.15–4.32 (m, 2 H), 6.84 (brs, 0.5 H), 6.90 (d, *J*=7.9 Hz, 1 H), 6.94 (t, *J*=7.7 Hz, 1 H), 7.14 (brs, 0.5 H), 7.26 (t, *J*=7.7 Hz, 1 H), 7.45 ppm (d, *J*=7.6 Hz, 1 H); ^13^C NMR (125 MHz, CDCl_3_): *δ*=23.7/24.5, 25.8, 28.2/28.3 (3C), 31.1, 41.5/41.6, 47.0/47.2, 60.3/61.2, 64.4/64.5, 69.0/69.4, 71.5/71.8, 108.9, 114.5, 121.1, 126.2, 126.3, 129.6, 130.9, 156.4, 175.7 ppm; HRMS (ESI): calcd for C_23_H_34_N_2_NaO_7_ *m*/*z* 473.2258 [*M*+Na]^+^, found *m*/*z* 473.2255 [*M*+Na]^+^.

Deprotection like for **5 a** yielded a yellowish semi‐solid (50 mg, 96 %); ^1^H NMR (500 MHz, CDCl_3_): *δ*=1.62–1.78 (m,2 H), 1.81–1.94 (m, 1 H), 2.08–2.21 (m, 1 H), 2.61 (s, 3 H), 2.81–2.95 (m, 1 H), 2.95–3.06 (m, 1 H), 3.38–3.49 (m, 1 H), 3.56–3.66 (m, 1 H), 3.72–3.87 (m, 1 H), 3.97–4.09 (m, 2 H), 4.09–4.19 (m, 1 H), 6.96 (d, *J*=8.5 Hz, 1 H), 7.01 (t, *J*=7.6 Hz, 1 H), 7.44 (t, *J*=7.7 Hz, 1 H), 7.68 (d, *J*=7.6 Hz, 1 H), 8.13 ppm (brs, 1 H); ^13^C NMR (125 MHz, CDCl_3_): *δ*=26.1, 30.8, 31.2, 42.8, 47.2, 60.3, 69.8, 70.6/70.7, 113.3, 121.0, 128.3, 130.4, 133.7, 157.7, 177.3, 199.9 ppm; HRMS (ESI): calcd for C_16_H_23_N_2_O_4_ *m*/*z* 307.1652 [*M*+H]^+^, found *m*/*z* 307.1657 [*M*+H]^+^.

Hydrochloride formation like for **5 a** yielded a brown honey (60 mg, 94 %); HRMS (ESI): calcd for C_16_H_23_N_2_O_4_ *m*/*z* 307.1652 [*M*+H]^+^, found *m*/*z* 307.1657 [*M*+H]^+^.

***N*** **‐(2‐(2‐(3‐phenylpropanoyl)phenoxy)ethyl)pyrrolidine‐2‐carboxamide (10 a)**: Preparation like **5 a**; purification by column chromatography (petrol ether:EtOAc=2:1 to 1:2) led to tert‐butyl 2‐((2‐(2‐(2‐phenethyl‐1,3‐dioxolan‐2‐yl)phenoxy)ethyl)carbamoyl)pyrrolidine‐1‐carboxylate as a yellowish honey (85 mg, 62 %); ^1^H NMR (500 MHz, CDCl_3_): *δ*=1.33 (brs, 6 H), 1.42 (brs, 3 H), 1.73–1.92 (m, 2 H), 1.95–2.02 (m, 1 H), 2.11–2.22 (m, 1 H), 2.38–2.52 (m, 2 H), 2.55–2.67 (m, 2 H), 3.42–3.55 (m, 2 H), 3.61–3.72 (m, 2 H), 3.81–3.95 (m, 2 H), 4.02–4.26 (m, 5 H), 6.90 (d, *J*=8.2 Hz, 1 H), 6.97 (t, *J*=7.4 Hz, 1 H), 7.08–7.17 (m, 3 H), 7.19–7.25 (m, 2 H), 7.28 (t, *J*=7.6 Hz, 1 H), 7.50 ppm (d, *J*=7.6 Hz, 1 H); ^13^C NMR (125 MHz, CDCl_3_): *δ*=23.8, 28.3 (3C), 30.2, 31.6, 39.0, 39.7, 46.8, 61.2, 64.7, 64.8, 67.9, 80.0, 110.3, 113.7, 120.9, 125.6, 127.6, 128.2 (4C), 129.6, 129.8, 142.1, 156.0, 173.3 ppm; HRMS (ESI): calcd for C_29_H_39_N_2_O_6_ *m*/*z* 511.2803 [*M*+H]^+^, found *m*/*z* 511.2807 [*M*+H]^+^.

Deprotection like for **5 a**; yellow honey (48 mg, quant.); ^1^H NMR (500 MHz, CDCl_3_): *δ*=1.62 (quin, *J*=6.9 Hz, 2 H), 1.78–1.86 (m, 1 H), 2.05–2.14 (m, 1 H), 2.69–2.78 (m, 1 H), 2.84–2.91 (m, 1 H), 3.05 (t, *J*=7.3 Hz, 2 H), 3.27–3.40 (m, 2 H), 3.56–3.74 (m, 3 H), 4.08–4.19 (m, 2 H), 6.93 (d, *J*=8.5 Hz, 1 H), 7.02 (t, *J*=7.6 Hz, 1 H), 7.19 (t, *J*=7.3 Hz, 1 H), 7.23 (d, *J*=7.0 Hz, 1 H), 7.26–7.32 (m, 2 H), 7.44 (ddd, *J*=8.7, 7.1, 1.6 Hz, 1 H), 7.66 (dd, *J*=7.9, 1.6 Hz, 1 H), 7.98 ppm (brs, 1 H); ^13^C NMR (125 MHz, CDCl_3_): *δ*=26.2, 30.3, 30.6, 38.4, 45.3, 47.2, 60.4, 67.3, 112.4, 121.1, 126.0, 128.4 (4C), 130.3, 132.3, 133.4, 141.7, 157.5, 174.2, 201.8 ppm; HRMS (ESI): calcd for C_22_H_27_N_2_O_3_ *m*/*z* 367.2016 [*M*+H]^+^, found *m*/*z* 367.2019 [*M*+H]^+^.

Hydrochloride formation like for **5 a** yielded a brown semisolid (47 mg, 98 %); HRMS (ESI): calcd for C_22_H_27_N_2_O_3_ *m*/*z* 367.2016 [*M*+H]^+^, found *m*/*z* 37.2020 [*M*+H]^+^.

***N*** **‐(2‐(4‐methoxy‐2‐(3‐phenylpropanoyl)phenoxy)ethyl)pyrrolidine‐2‐carboxamide (10 b)**: Preparation like **5 a**; purification by column chromatography (petrol ether:EtOAc=1:3 to 1:5) led to tert‐butyl 2‐((2‐(5‐methoxy‐2‐(2‐phenethyl‐1,3‐dioxolan‐2‐yl)phenoxy)ethyl)carbamoyl)pyrrolidine‐1‐carboxylate as a colorless oil (989 mg, 78 %); ^1^H NMR (500 MHz, CDCl_3_): *δ*=1.33 (brs, 6 H), 1.42 (brs, 3 H), 1.75–1.84 (m, 1 H), 1.84–1.92 (m, 1 H), 1.92–2.03 (m, 1 H), 2.11–2.22 (m, 1 H), 2.34–2.47 (m, 2 H), 2.52–2.66 (m, 2 H), 3.41–3.55 (m, 2 H), 3.62–3.70 (m, 2 H), 3.80 (s, 3 H), 3.82–3.94 (m, 2 H), 3.99–4.23 (m, 5 H), 6.46 (s, 1 H), 6.48 (d, *J*=8.8 Hz, 1 H), 7.09–7.17 (m, 3 H), 7.20–7.25 (m, 2 H), 7.40 ppm (d, *J*=8.2 Hz, 1 H); ^13^C NMR (125 MHz, CDCl_3_): *δ*=23.8, 28.3 (3C), 30.3, 31.6, 38.8, 39.9, 46.8, 55.4, 61.2, 64.6, 64.7, 67.9, 80.1, 101.2, 104.6, 110.2, 122.3, 125.6, 128.2 (4C), 128.4, 142.3, 156.7, 160.8, 171.3 ppm; HRMS (ESI): calcd for C_30_H_41_N_2_O_7_ *m*/*z* 541.2908 [*M*+H]^+^, found *m*/*z* 541.2911 [*M*+H]^+^.

Deprotection like for **5 a** yielded a yellow semisolid (356 mg, 98 %); ^1^H NMR (500 MHz, CDCl_3_): *δ*=1.56–1.64 (m, 2 H), 1.76–1.86 (m, 1 H), 2.01–2.12 (m, 1 H), 2.67–2.75 (m, 1 H), 2.81–2.89 (m, 1 H), 3.03 (t, *J*=7.7 Hz, 2 H), 3.21–3.38 (m, 2 H), 3.53–3.59 (m, 1 H), 3.59–3.68 (m, 2 H), 3.84 (s, 3 H), 4.05–4.18 (m, 2 H), 6.42 (s, 1 H), 6.54 (d, *J*=8.8 Hz, 1 H), 7.18 (t, *J*=7.1 Hz, 1 H), 7.23 (d, *J*=7.3 Hz, 2 H), 7.28 (d, *J*=7.6 Hz, 2 H), 7.81 (d, *J*=8.5 Hz, 1 H), 7.99 ppm (brs, 1 H); ^13^C NMR (125 MHz, CDCl_3_): *δ*=26.1, 30.4, 30.6, 38.3, 45.2, 47.2, 55.5, 60.4, 67.3, 98.9, 105.7, 121.0, 125.9, 128.3 (2C), 128.4 (2C), 132.7, 141.9, 159.6, 164.3, 175.6, 199.1 ppm; HRMS (ESI): calcd for C_23_H_29_N_2_O_4_ *m*/*z* 397.2122 [*M*+H]^+^, found *m*/*z* 397.2126 [*M*+H]^+^.

Hydrochloride formation like for **5 a** yielded a brown honey (286 mg, 99 %); HRMS (ESI): calcd for C_23_H_29_N_2_O_4_ *m*/*z* 397.2122 [*M*+H]^+^, found *m*/*z* 397.2124 [*M*+H]^+^.

***N*** **‐(2‐(2‐acetylphenoxy)ethyl)pyrrolidine‐2‐carboxamide (10 c)**: Preparation like **5 a**, purification by column chromatography (CH_2_Cl_2_:MeOH=40:1 to 25:1) yielded tert‐butyl 2‐((2‐(2‐(2‐methyl‐1,3‐dioxolan‐2‐yl)phenoxy)ethyl)carbamoyl)pyrrolidine‐1‐carboxylate as a colorless oil (215 mg, 57 %); ^1^H NMR (500 MHz, CDCl_3_): *δ*=1.36 (brs, 6 H), 1.43 (brs, 3 H), 1.77 (s, 3 H), 1.81–1.96 (m, 2 H), 1.97–2.07 (m, 1 H), 2.16–2.25 (m, 1 H), 3.44–3.57 (m, 2 H), 3.64–3.74 (m, 2 H), 3.77–3.89 (m, 2 H), 4.03–4.18 (m, 4.5 H), 4.22–4.29 (m, 0.5 H), 6.91 (d, *J*=8.2 Hz, 1 H), 6.95 (t, *J*=7.1 Hz, 1 H), 7.27 (t, *J*=7.1 Hz, 1 H), 7.48 ppm (d, *J*=7.9 Hz, 1 H); ^13^C NMR (125 MHz, CDCl_3_): *δ*=23.8, 25.6, 28.3(3C), 31.5, 39.0, 46.9, 61.3, 64.4 (2C), 68.1, 80.2, 108.7, 114.1, 121.1, 126.8, 129.5, 131.1, 155.9, 165.7 ppm; HRMS (ESI): calcd for C_22_H_33_N_2_O_6_ *m*/*z* 421.2333 [*M*+H]^+^, found *m*/*z* 421.2338 [*M*+H]^+^.

Deprotection like **5 a** yielded a yellowish solid (75 mg, 97 %); ^1^H NMR (500 MHz, CDCl_3_): *δ*=1.66–1.77 (m, 2 H), 1.85–1.97 (m, 1 H), 2.11–2.23 (m, 1 H), 2.64 (s, 3 H), 2.84–2.94 (m, 1 H), 2.98–3.07 (m, 1 H), 3.62–3.77 (m, 2 H), 3.78–3.85 (m, 1 H), 4.16 (t, *J*=4.7 Hz, 2 H), 6.94 (d, *J*=8.2 Hz, 1 H), 7.02 (t, *J*=7.6 Hz, 1 H), 7.45 (t, *J*=7.9 Hz, 1 H), 7.73 (d, *J*=7.9 Hz, 1 H), 8.13 ppm (brs, 1 H); ^13^C NMR (125 MHz, CDCl_3_): *δ*=26.1, 30.6, 31.8, 38.5, 47.1, 60.4, 67.3, 112.6, 121.0, 128.5, 130.5, 133.7, 157.8, 170.7, 199.7 ppm; HRMS (ESI): calcd for C_15_H_21_N_2_NaO_3_ *m*/*z* 299.1366 [*M*+Na]^+^, found *m*/*z* 299.1368 [*M*+Na]^+^.

Hydrochloride formation like for **5 a** yielded a brown semisolid (86 mg, 98 %); HRMS (ESI): calcd for C_15_H_21_N_2_NaO_3_ *m*/*z* 299.1366 [*M*+Na]^+^, found *m*/*z* 299.1368 [*M*+Na]^+^.

**2‐(2‐Hydroxy‐3‐(2‐(3‐phenylpropanoyl)phenoxy)propyl)‐hexahydropyrrolo[1,2‐a]pyrazine‐1,4‐dione (6 a)**: The amine **4 a** (371 mg, 1 eq, 1.08 mmol), methyl (2‐chloroacetyl)prolinate (223 mg, 1 eq, 1.08 mmol), and Et_3_N (0.16 mL, 1.2 eq, 1.13 mmol) were dissolved in CH_3_CN (40 mL) and stirred at RT for 2.5 d. The solvent was evaporated, and the residue was redissolved in EtOAc and extracted with water. The aqueous phase was extracted with EtOAc (3×50 mL), the combined organic phases were dried over Na_2_SO_4_, and the solvent was evaporated. The product was purified by column chromatography (CH_2_Cl_2_ to CH_2_Cl_2_:MeOH=20:1) twice to yield a yellow semisolid (30 mg, 6 %).

2‐(2‐Hydroxy‐3‐(2‐(2‐phenethyl‐1,3‐dioxolan‐2‐yl)phenoxy)propyl)hexahydropyrrolo[1,2‐a]pyrazine‐1,4‐dione: *Diastereomer 1*: ^1^H NMR (500 MHz, CDCl_3_): *δ*=1.85–1.96 (m, 1 H), 2.00–2.14 (m, 2 H), 2.35–2.43 (m, 1 H), 2.41 (t, *J*=8.7 Hz, 2 H), 2.60–2.68 (m, 2 H), 3.26 (dd, *J*=14.1, 7.4 Hz, 1 H), 3.51–3.59 (m, 1 H), 3.59–3.67 (m, 1 H), 3.82–3.92 (m, 4 H), 4.05–4.13 (m, 3 H), 4.04 (d, *J*=17.0 Hz, 1 H), 4.16–4.19 (m, 1 H), 4.23 (dd, *J*=9.5, 3.2 Hz, 1 H), 4.42 (d, *J*=16.7 Hz, 1 H), 6.90 (dd, *J*=8.2, 2.8 Hz, 1 H), 6.98 (t, *J*=7.6 Hz, 1 H), 7.10–7.17 (m, 3 H), 7.20–7.26 (m, 2 H), 7.28 (t, *J*=7.7 Hz, 1 H), 7.48 ppm (d, *J*=7.6 Hz, 1 H); ^13^C NMR (125 MHz, CDCl_3_): *δ*=22.7, 28.9, 30.0, 40.1, 45.2, 49.7, 54.5, 59.1, 64.8 (2C), 69.7, 72.1, 110.4, 114.7, 121.3, 125.6, 127.1, 128.3 (4C), 129.8, 130.0, 142.0, 156.5, 163.6, 168.0 ppm; *Diastereomer 2*: ^1^H NMR (500 MHz, CDCl_3_): *δ*=1.85–1.96 (m, 1 H), 2.00–2.14 (m, 2 H), 2.35–2.43 (m, 1 H), 2.41 (t, *J*=8.7 Hz, 2 H), 2.60–2.68 (m, 2 H), 3.51–3.59 (m, 1 H), 3.59–3.67 (m, 3 H), 3.82–3.92 (m, 3 H), 4.05–4.13 (m, 3 H), 4.14 (d, *J*=17.0 Hz, 1 H), 4.18–4.22 (m, 2 H), 4.29 (d, *J*=16.4 Hz, 1 H), 6.90 (dd, *J*=8.2, 2.8 Hz, 1 H), 6.98 (t, *J*=7.6 Hz, 1 H), 7.10–7.17 (m, 3 H), 7.20–7.26 (m, 2 H), 7.28 (t, *J*=7.7 Hz, 1 H), 7.48 ppm (d, *J*=7.6 Hz, 1 H); ^13^C NMR (125 MHz, CDCl_3_): *δ*=22.6, 28.9, 30.0, 40.0, 45.2, 48.9, 54.0, 58.9, 64.8 (2C), 69.5, 72.0, 110.3, 114.7, 121.2, 125.6, 127.1, 128.3 (4C), 129.7, 130.0, 142.0, 156.5, 163.4, 168.0 ppm; HRMS (ESI): calcd for C_27_H_32_N_2_NaO_6_ *m*/*z* 503.2153 [*M*+Na]^+^, found *m*/*z* 503.2151 [*M*+Na]^+^.

The ketal (24 mg, 1 eq, 0.05 mmol) was dissolved in CH_3_CN (2 mL), and aq. HCl solution (2 N, 2 mL) was added, and it was stirred for 4 h at RT. Then it was diluted with water and extracted with EtOAc (4×50 mL), dried over Na_2_SO_4_, and evaporated to yield a colorless solid (21 mg, 96 %). *Diastereomer 1*: ^1^H NMR (500 MHz, CDCl_3_): *δ*=1.86–1.97 (m, 1 H), 2.00–2.12 (m, 2 H), 2.35–2.43 (m, 1 H), 3.04 (t, *J*=7.9 Hz, 2 H), 3.23–3.29 (m, 2 H), 3.31 (dd, *J*=14.5, 7.9 Hz, 1 H), 3.54–3.65 (m, 2 H), 3.79 (dd, *J*=14.1, 3.0 Hz, 1 H), 3.93–4.01 (m, 1 H), 3.96 (d, *J*=16.7 Hz, 1 H), 4.07–4.15 (m, 2 H), 4.17–4.22 (m, 1 H), 4.33 (d, *J*=17.0 Hz, 1 H), 6.96 (d, *J*=8.2 Hz, 1 H), 7.02 (t, *J*=7.6 Hz, 1 H), 7.16–7.22 (m, 1 H), 7.23 (d, *J*=7.3 Hz, 2 H), 7.28 (td, *J*=7.5, 1.6 Hz, 2 H), 7.44 (t, *J*=7.9 Hz, 1 H), 7.60 ppm (ddd, *J*=7.7, 4.6, 1.7 Hz, 1 H); ^13^C NMR (125 MHz, CDCl_3_): *δ*=22.6, 28.8, 30.1, 44.4, 45.2, 50.2, 54.4, 59.0, 69.5, 71.5, 114.3, 121.4, 126.1, 128.4 (2C), 128.5, 128.5 (2C), 130.0, 133.5, 141.3, 157.3, 163.4, 168.7, 201.5 ppm; *Diastereomer 2*: ^1^H NMR (500 MHz, CDCl_3_): *δ*=1.86–1.97 (m, 1 H), 2.00–2.12 (m, 2 H), 2.35–2.43 (m, 1 H), 3.04 (t, *J*=7.9 Hz, 2 H), 3.23–3.29 (m, 2 H), 3.52 (dd, *J*=14.4, 3.3 Hz, 1 H), 3.54–3.65 (m, 2 H), 3.68 (dd, *J*=14.2, 6.6 Hz, 1 H), 3.93–4.01 (m, 1 H), 4.01 (d, *J*=16.7 Hz, 1 H), 4.07–4.15 (m, 2 H), 4.16–4.20 (m, 1 H), 4.23 (d, *J*=16.7 Hz, 1 H), 6.96 (d, *J*=8.2 Hz, 1 H), 7.02 (t, *J*=7.6 Hz, 1 H), 7.16–7.22 (m, 1 H), 7.23 (d, *J*=7.3 Hz, 2 H), 7.28 (td, *J*=7.5, 1.6 Hz, 2 H), 7.44 (t, *J*=7.9 Hz, 1 H), 7.60 ppm (ddd, *J*=7.7, 4.6, 1.7 Hz, 1 H); ^13^C NMR (125 MHz, CDCl_3_): *δ*=22.6, 28.8, 30.1, 44.3, 45.2, 49.6, 53.9, 58.9, 69.3, 71.4, 114.2, 121.4, 126.1, 127.0, 128.4 (2C), 128.5, 128.5 (2C), 133.5, 141.2, 157.3, 163.3, 168.5, 201.4 ppm; HRMS (ESI): calcd for C_25_H_28_N_2_NaO_5_ *m*/*z* 459.1890 [*M*+Na]^+^, found *m*/*z* 459.1893 [*M*+Na]^+^.

**2‐(2‐Hydroxy‐3‐(5‐methoxy‐2‐(3‐phenylpropanoyl)phenoxy)propyl)‐hexahydropyrrolo[1,2‐a]pyrazine‐1,4‐dione (6 b)**: The amine **4 b** (300 mg, 1 eq, 0.8 mmol), methyl (2‐chloroacetyl)prolinate (165 mg, 1 eq, 0.8 mmol), and Et_3_N (0.14 mL, 1.3 eq, 1.0 mmol) were dissolved in 2‐ethoxyethanol (6 mL) and heated at reflux for 3 d. The solvent was evaporated and the residue was redissolved in a mixture of CH_2_Cl_2_ and toluene and extracted with water. The aqueous phase was extracted with CH_2_Cl_2_ (3×60 mL), the combined organic phases were washed once with brine (70 mL), dried over Na_2_SO_4_ and the solvent was evaporated. The product was purified by column chromatography (CH_2_Cl_2_ to CH_2_Cl_2_:MeOH=30:1) to yield 251 mg of a yellow solid that was already partially deprotected.

2‐(2‐hydroxy‐3‐(5‐methoxy‐2‐(2‐phenethyl‐1,3‐dioxolan‐2‐yl)phenoxy)propyl)hexahydropyrrolo[1,2‐a]pyrazine‐1,4‐dione: HRMS (ESI): calcd for C_28_H_34_N_2_NaO_7_ *m*/*z* 533.2258 [*M*+Na]^+^, found *m*/*z* 533.2252 [*M*+Na]^+^.

The mixture from the previous step (120 mg, 1 eq, 0.07 mmol) was dissolved in CH_3_CN (9 mL); aq. HCl solution (2 N, 9 mL) was added and it was stirred for 4 h at RT. Then it was diluted with water (5 mL) and aq. NaHCO_3_, (10 mL), extracted with EtOAc (4×30 mL), dried over Na_2_SO_4_, and evaporated to yield a yellowish solid (109 mg, 61 %, 2 steps). *Diastereomer 1*: ^1^H NMR (500 MHz, CDCl_3_): *δ*=1.86–1.97 (m, 1 H), 2.00–2.12 (m, 2 H), 2.34–2.43 (m, 1 H), 3.02 (t, *J*=7.6 Hz, 1 H), 3.19–3.25 (m, 2 H), 3.30 (dd, *J*=14.2, 7.6 Hz, 1 H), 3.54–3.65 (m, 2 H), 3.81 (dd, *J*=14.3, 3.0 Hz, 1 H), 3.84 (s, 3 H), 3.91–4.03 (m, 1 H), 3.93 (d, *J*=17.1 Hz, 1 H), 4.07–4.15 (m, 2 H), 4.21–4.27 (m, 1 H), 4.32 (d, *J*=17.0 Hz, 1 H), 6.45 (d, *J*=2.2 Hz, 1 H), 6.53 (dd, *J*=8.7, 2.0 Hz, 1 H), 7.18 (dd, *J*=8.1, 5.9 Hz, 1 H), 7.23 (d, *J*=7.3 Hz, 2 H), 7.28 (td, *J*=7.6, 1.7 Hz, 2 H), 7.57 ppm (t, *J*=8.4 Hz, 1 H); ^13^C NMR (125 MHz, CDCl_3_): *δ*=22.7, 28.8, 30.4, 44.1, 45.2, 50.3, 54.4, 55.6, 59.0, 69.4, 71.6, 101.0, 106.1, 126.0, 128.4 (2C), 128.5 (2C), 132.5, 141.5, 159.8, 163.3, 164.2, 168.7, 199.2 ppm; *Diastereomer 2*: ^1^H NMR (500 MHz, CDCl_3_): *δ*=1.86–1.97 (m, 1 H), 2.00–2.12 (m, 2 H), 2.34–2.43 (m, 1 H), 3.02 (t, *J*=7.6 Hz, 1 H), 3.19–3.25 (m, 2 H), 3.52 (dd, *J*=14.2, 7.6 Hz, 1 H), 3.54–3.65 (m, 2 H), 3.68 (dd, *J*=14.3, 3.0 Hz, 1 H), 3.84 (s, 3 H), 3.91–4.03 (m, 1 H), 3.98 (d, *J*=17.1 Hz, 1 H), 4.07–4.15 (m, 2 H), 4.15–4.21 (m, 1 H), 4.21 (d, *J*=17.0 Hz, 1 H), 6.45 (d, *J*=2.2 Hz, 1 H), 6.53 (dd, *J*=8.7, 2.0 Hz, 1 H), 7.18 (dd, *J*=8.1, 5.9 Hz, 1 H), 7.23 (d, *J*=7.3 Hz, 2 H), 7.28 (td, *J*=7.6, 1.7 Hz, 2 H), 7.57 ppm (t, *J*=8.4 Hz, 1 H); ^13^C NMR (125 MHz, CDCl_3_): *δ*=22.6, 28.8, 30.4, 44.0, 45.2, 49.7, 53.9, 55.6, 58.9, 69.2, 71.5, 100.9, 106.0, 126.0, 128.4 (2C), 128.5 (2C), 132.4, 141.5, 159.8, 163.2, 164.2, 168.5, 199.1 ppm; HRMS (ESI): calcd for C_26_H_30_N_2_NaO_6_ *m*/*z* 489.1996 [*M*+Na]^+^, found *m*/*z* 489.2004 [*M*+Na]^+^.

**2‐(3‐(2‐Acetylphenoxy)‐2‐hydroxypropyl)hexahydropyrrolo[1,2‐a]‐pyrazine‐1,4‐dione (6 c)**: Preparation like **6 b**. The product was purified by column chromatography (CH_2_Cl_2_ to CH_2_Cl_2_:MeOH=30:1) to yield a colorless oil (81 mg, yield given over two steps below) that was already partially deprotected: 2‐(2‐hydroxy‐3‐(2‐(2‐methyl‐1,3‐dioxolan‐2‐yl)phenoxy)propyl) hexahydropyrrolo[1,2‐a]pyrazine‐1,4‐dione; HRMS (ESI): calcd for C_20_H_26_N_2_NaO_6_ *m*/*z* 413.1683 [*M*+Na]^+^, found *m*/*z* 413.1679 [*M*+Na]^+^.

Deprotection like **6 b** yielded a yellowish solid (23 mg, 35 %, 2 steps). *Diastereomer 1*: ^1^H NMR (500 MHz, CDCl_3_): *δ*=1.87–1.97 (m, 1 H), 2.00–2.14 (m, 2 H), 2.36–2.44 (m, 1 H), 2.62 (s, 3 H), 3.57 (dd, *J*=13.9, 3.5 Hz, 1 H), 3.53–3.59 (m, 1 H), 3.59–3.66 (m, 1 H), 3.80 (dd, *J*=14.3, 6.8 Hz, 1 H), 3.97–4.04 (m, 1 H), 4.07 (d, *J*=16.7 Hz, 1 H), 4.10–4.19 (m, 2 H), 4.22–4.31 (m, 1 H), 4.34 (d, *J*=16.1 Hz, 1 H), 6.96 (d, *J*=8.2 Hz, 1 H), 7.05 (t, *J*=7.4 Hz, 1 H), 7.46 (t, *J*=7.9 Hz, 1 H), 7.70 ppm (ddd, *J*=7.7, 2.0, 1.7 Hz, 1 H); ^13^C NMR (125 MHz, CDCl_3_): *δ*=22.7, 28.8, 30.8, 45.2, 49.6, 54.0, 59.0, 69.5, 71.4, 114.2, 121.4, 130.6, 133.8, 128.5, 157.4, 163.2, 168.6, 198.2 ppm; *Diastereomer 2*: ^1^H NMR (500 MHz, CDCl_3_): *δ*=1.87–1.97 (m, 1 H), 2.00–2.14 (m, 2 H), 2.36–2.44 (m, 1 H), 2.61 (s, 3 H), 3.42 (dd, *J*=14.2, 7.9 Hz, 1 H), 3.53–3.59 (m, 1 H), 3.59–3.66 (m, 1 H), 3.85 (dd, *J*=14.2, 3.2 Hz, 1 H), 3.97–4.04 (m, 1 H), 4.03 (d, *J*=17.0 Hz, 1 H), 4.10–4.19 (m, 2 H), 4.22–4.31 (m, 1 H), 4.42 (d, *J*=16.4 Hz, 1 H), 6.96 (d, *J*=8.2 Hz, 1 H), 7.05 (t, *J*=7.4 Hz, 1 H), 7.46 (t, *J*=7.9 Hz, 1 H), 7.70 ppm (ddd, *J*=7.7, 2.0, 1.7 Hz, 1 H); ^13^C NMR (125 MHz, CDCl_3_): *δ*=22.7, 28.8, 30.7, 45.2, 50.4, 54.5, 58.9, 69.3, 71.3, 114.1, 121.4, 130.6, 133.8, 128.5, 157.4, 163.2, 168.6, 198.2 ppm; HRMS (ESI): calcd for C_18_H_22_N_2_NaO_5_ *m*/*z* 369.1421 [*M*+Na]^+^, found *m*/*z* 369.1427 [*M*+Na]^+^.

**2‐(2‐(2‐(3‐Phenylpropanoyl)phenoxy)ethyl)hexahydropyrrolo[1,2‐a]‐pyrazine‐1,4‐dione (11 a)**: Preparation like **6 b** yielded a mixture of partially deprotected 2‐(2‐(2‐(2‐phenethyl‐1,3‐dioxolan‐2‐yl)phenoxy)ethyl)hexahydropyrrolo[1,2‐a]pyrazine‐1,4‐dione (114 mg, partially deprotected, therefore yield given for two steps below); HRMS (ESI): calcd for C_26_H_30_N_2_NaO_5_ *m*/*z* 473.2047 [*M*+Na]^+^, found *m*/*z* 473.2050 [*M*+Na]^+^.

Deprotection like **6 b** yielded a yellowish semisolid (80 mg, 39 %, 2 steps); ^1^H NMR (500 MHz, CDCl_3_): *δ*=1.82–1.94 (m, 1 H), 1.97–2.08 (m, 2 H), 2.29–2.37 (m, 1 H), 3.02 (t, *J*=7.7 Hz, 2 H), 3.23–3.29 (m, 2 H), 3.49–3.61 (m, 3 H), 3.86 (d, *J*=16.4 Hz, 1 H), 3.94 (t, *J*=7.7 Hz, 1 H), 4.02 (ddd, *J*=14.3, 5.8, 4.3 Hz, 1 H), 4.15 (ddd, *J*=10.1, 5.7, 4.4 Hz, 1 H), 4.23 (d, *J*=16.4 Hz, 1 H) 4.23–4.29 (m, 1 H), 6.92 (d, *J*=8.2 Hz, 1 H), 7.01 (t, *J*=7.6 Hz, 1 H), 7.18 (t, *J*=7.1 Hz, 1 H), 7.23 (d, *J*=7.0 Hz, 2 H), 7.26–7.30 (m, 2 H), 7.42 (ddd, *J*=8.4, 7.4, 1.7 Hz, 1 H), 7.57 ppm (dd, *J*=7.7, 1.7 Hz, 1 H); ^13^C NMR (125 MHz, CDCl_3_): *δ*=22.6, 28.6, 30.2, 45.0, 45.2, 46.1, 53.4, 58.9, 65.8, 112.5, 121.3, 126.0, 128.4 (4C), 129.0, 130.0, 133.1, 141.4, 156.6, 163.0, 167.8, 201.5 ppm; HRMS (ESI): calcd for C_24_H_26_N_2_NaO_4_ *m*/*z* 429.1785 [*M*+Na]^+^, found *m*/*z* 429.1788 [*M*+Na]^+^.

**2‐(2‐(5‐Methoxy‐2‐(3‐phenylpropanoyl)phenoxy)ethyl)‐hexahydropyrrolo[1,2‐a]pyrazine‐1,4‐dione (11 b)**: Preparation like **6 b** yielded a brown solid—mixture of partially deprotected tert‐butyl 2‐((2‐(5‐methoxy‐2‐(2‐phenethyl‐1,3‐dioxolan‐2‐yl)phenoxy)‐ethyl)carbamoyl)pyrrolidine‐1‐carboxylate (96 mg, yield given for two steps below); HRMS (ESI): calcd for C_27_H_32_N_2_NaO_6_ *m*/*z* 503.2153 [*M*+Na]^+^, found *m*/*z* 503.2155 [*M*+Na]^+^.

Deprotection like **6 b** yielded a yellowish semisolid (66 mg, 25 %, 2 steps); ^1^H NMR (500 MHz, CDCl_3_): *δ*=1.82–1.94 (m, 1 H), 1.96–2.09 (m, 2 H), 2.29–2.38 (m, 1 H), 3.01 (t, *J*=8.1 Hz, 2 H), 3.19–3.27 (m, 2 H), 3.47–3.61 (m, 3 H), 3.83 (s, 3 H), 3.86 (d, *J*=16.4 Hz, 1 H), 3.92 (t, *J*=7.9 Hz, 1 H), 3.96–4.03 (m, 1 H), 4.08–4.17 (m, 1 H), 4.23 (d, *J*=17.0 Hz, 1 H), 4.22–4.28 (m, 1 H), 6.43 (d, *J*=2.3 Hz, 1 H), 6.53 (dd, *J*=8.9, 2.2 Hz, 1 H), 7.17 (t, *J*=7.1 Hz, 1 H), 7.23 (d, *J*=7.3 Hz, 2 H), 7.28 (t, *J*=7.6 Hz, 2 H), 7.72 ppm (d, *J*=8.8 Hz, 1 H); ^13^C NMR (125 MHz, CDCl_3_): *δ*=22.6, 28.6, 30.4, 44.9, 45.2, 46.1, 53.4, 55.6, 58.9, 65.8, 99.3, 105.8, 121.4, 125.9, 128.4 (2C), 128.5 (2C), 132.6, 159.0, 162.9, 164.1, 167.8, 198.9 ppm; HRMS (ESI): calcd for C_25_H_28_N_2_NaO_5_ *m*/*z* 459.1890 [*M*+Na]^+^, found *m*/*z* 459.1894 [*M*+Na]^+^.

**2‐(2‐(2‐Acetylphenoxy)ethyl)hexahydropyrrolo[1,2‐a]pyrazine‐1,4‐dione (11 c)**: The amine **9 c** (200 mg, 1 eq, 0.9 mmol), methyl (2‐chloroacetyl)prolinate (184 mg, 1 eq, 0.9 mmol), and Et_3_N (0.16 mL, 1.3 eq, 1.2 mmol) were dissolved in 2‐ethoxyethanol (6 mL) and heated at reflux for 24 h. The solvent was evaporated, and the residue was redissolved in a mixture of CH_2_Cl_2_ and toluene (2:1, 100 mL) and extracted once with water (50 mL). The aqueous phase was extracted CH_2_Cl_2_ (3×50 mL). The combined organic phases were washed once with brine (70 mL), dried over Na_2_SO_4_, and the solvent was evaporated. The product was purified by column chromatography (CH_2_Cl_2_ to CH_2_Cl_2_:MeOH=20:1) twice to yield a yellow semisolid (65 mg, 20 %).

2‐(2‐(2‐(2‐methyl‐1,3‐dioxolan‐2‐yl)phenoxy)ethyl)hexahydropyrrolo[1,2‐a]pyrazine‐1,4‐dione: ^1^H NMR (500 MHz, CDCl_3_): *δ*=1.73 (s, 3 H), 1.85–1.97 (m, 1 H), 1.99–2.12 (m, 2 H), 2.36–2.44 (m, 1 H), 3.51–3.58 (m, 1 H), 3.59–3.67 (m, 2 H), 3.77–3.85 (m, 2 H), 4.00–4.04 (m, 2 H), 4.04–4.08 (m, 1 H), 4.09–4.13 (m, 1 H), 4.13–4.17 (m, 1 H), 4.20 (d, *J*=17.0 Hz, 1 H), 4.22 −4.28 (m, 1 H), 4.55 (dd, *J*=17.0, 1.9 Hz, 1 H), 6.87 (dd, *J*=8.2, 0.6 Hz, 1 H), 6.94 (td, *J*=7.5, 1.1 Hz, 1 H), 7.26 (ddd, *J*=8.1, 7.4, 1.8 Hz, 1 H), 7.51 ppm (dd, *J*=7.6, 1.8 Hz); ^13^C NMR (125 MHz, CDCl_3_): *δ*=22.6, 25.6, 28.8, 45.2, 46.6, 53.8, 59.0, 64.5 (2C), 66.4, 108.3, 112.8, 120.8, 126.8, 129.4, 130.8, 156.7, 163.7, 167.6 ppm; HRMS (ESI): calcd for C_19_H_24_N_2_NaO_5_ *m*/*z* 383.1577 [*M*+Na]^+^, found *m*/*z* 383.1583 [*M*+Na]^+^.

The ketal (35 mg, 1 eq, 0.09 mmol) was dissolved in CH_3_CN (2 mL); aq. HCl solution (2 n, 2 mL) was added, and it was stirred for 4 h at RT. Then it was diluted with water and extracted with EtOAc (4×40 mL), dried over Na_2_SO_4_, and evaporated to yield a colorless solid (30 mg, 97 %); ^1^H NMR (500 MHz, CDCl_3_): *δ*=1.85–1.97 (m, 1 H), 1.99–2.12 (m, 2 H), 2.35–2.43 (m, 1 H), 2.59 (s, 3 H), 3.51–3.64 (m, 2 H), 3.68–3.76 (m, 1 H), 3.99 (d, *J*=16.7 Hz, 1 H), 3.98–4.05 (m, 1 H), 4.10 (t, *J*=7.6 Hz, 1 H), 4.16–4.23 (m, 1 H), 4.25–4.30 (m, 1 H), 4.33 (d, *J*=16.7 Hz, 1 H), 6.94 (d, *J*=8.2 Hz, 1 H), 7.02 (t, *J*=7.4 Hz, 1 H), 7.44 (ddd, *J*=7.7, 7.4, 1.8 Hz, 1 H), 7.67 ppm (dd, *J*=7.7, 1.7 Hz, 1 H); ^13^C NMR (125 MHz, CDCl_3_): *δ*=22.6, 28.7, 31.4, 45.2, 46.1, 53.3, 59.0, 65.8, 112.5, 121.2, 130.3, 133.4, 128.8, 157.0, 163.1, 167.8, 199.6 ppm; HRMS (ESI): calcd for C_17_H_20_N_2_NaO_4_ *m*/*z* 339.1315 [*M*+Na]^+^, found *m*/*z* 339.1321 [*M*+Na]^+^.

**2‐(2‐(2‐Bromoethoxy)phenyl)‐2‐phenethyl‐1,3‐dioxolane (13 a)**: Preparation of the ketal from 1‐(2‐(2‐bromoethoxy)phenyl)‐3‐phenylpropan‐1‐one like for **1 a** yielded a yellow oil (2.79 g, 99 %); ^1^H NMR (500 MHz, CDCl_3_): *δ*=2.48–2.55 (m, 2 H), 2.61–2.68 (m, 2 H), 3.68 (t, *J*=6.3 Hz, 2 H), 3.83–3.92 (m, 2 H), 4.02–4.11 (m, 2 H), 4.33 (t, *J*=6.5 Hz, 2 H), 6.89 (d, *J*=8.2 Hz, 1 H), 6.97 (t, *J*=7.6 Hz, 1 H), 7.13 (t, *J*=7.4 Hz, 1 H), 7.16 (d, *J*=7.6 Hz, 2 H), 7.23 (t, *J*=7.4 Hz, 2 H), 7.27 (t, *J*=7.3 Hz, 1 H), 7.54 ppm (d, *J*=7.6 Hz, 1 H); ^13^C NMR (125 MHz, CDCl_3_): *δ*=29.1, 30.1, 39.6, 64.8 (2C), 68.8, 109.9, 113.6, 121.0, 125.5, 127.7, 128.2 (2C), 128.4 (2C), 129.5, 130.3, 142.4, 155.7 ppm; HRMS (ESI): calcd for C_19_H_22_BrO_3_ *m*/*z* 377.0747 [*M*+H]^+^, found *m*/*z* 377.0747 [*M*+H]^+^.

**2,2,2‐Trifluoro‐*N*‐(2‐(2‐(2‐phenethyl‐1,3‐dioxolan‐2‐yl)phenoxy)ethyl)‐acetamide (14 a)**: The bromide **13 a** (1 g, 1 eq, 2.65 mmol), trifluoroacetamide (599 mg, 2 eq, 5.30 mmol), and tetrabutylammonium bromide (85 mg, 0.1 eq, 0.27 mmol) were dissolved in dimethylformamide (DMF, 5 mL), and K_2_CO_3_ (733 mg, 2 eq, 5.3 mmol) was added. The mixture was heated at 80 °C for 4.5 h, then TLC indicated completion, and it was filtrated and evaporated. The crude product was purified by column chromatography (petrol ether:EtOAc=30:1 to 4:1) to yield a colorless oil (708 mg, 65 %); ^1^H NMR (500 MHz, CDCl_3_): *δ*=2.42–2.50 (m, 2 H), 2.62–2.69 (m, 2 H), 3.76 (dd, *J*=9.8, 5.0 Hz, 2 H), 3.83–3.94 (m, 2 H), 4.07–4.13 (m, 2 H), 4.16 (t, *J*=5.0 Hz, 2 H), 6.93 (d, *J*=8.2 Hz, 1 H), 7.02 (t, *J*=7.6 Hz, 1 H), 7.12–7.19 (m, 3 H), 7.25 (t, *J*=7.4 Hz, 2 H), 7.31 (t, *J*=7.7 Hz, 1 H), 7.52 (d, *J*=7.9 Hz, 1 H), 8.31 ppm (brs, 1 H); ^13^C NMR (125 MHz, CDCl_3_): *δ*=29.9, 39.6, 39.9, 64.8 (2C), 67.1, 110.3, 114.3, 116.0 (q, *J*=287.7 Hz), 121.6, 125.6, 127.1, 128.2 (2C), 128.3 (2C), 129.7, 130.4, 142.0, 155.7, 157.4 ppm (q, *J*=36.9 Hz); HRMS (ESI): calcd for C_21_H_22_F_3_NNaO_4_ *m*/*z* 432.1393 [*M*+Na]^+^, found *m*/*z* 432.1397 [*M*+Na]^+^.

**2‐(2‐(2‐Phenethyl‐1,3‐dioxolan‐2‐yl)phenoxy)ethan‐1‐amine (9 a), Method B**: The trifluoroacetamide derivative **14 a** (400 mg, 1 eq, 0.98 mmol) was dissolved in MeOH (2 mL) and water (2 mL), and KOH (110 mg, 2 eq, 1.95 mmol) was added and stirred at RT for 2 h. Then the reaction mixture was concentrated and extracted with EtOAc (3×50 mL). The combined organic extracts were dried over Na_2_SO_4_ and evaporated to yield **9 a** as a colorless oil (305 mg, 99 %).

**1‐(2‐(2‐Hydroxy‐3‐(isopropylamino)propoxy)phenyl)‐3‐phenyl‐propan‐1‐one (16 a)**: 1‐(2‐(Oxiran‐2‐ylmethoxy)phenyl)‐3‐phenylpropan‐1‐one (514 mg, 1.8 mmol) was dissolved in *i*PrNH_2_ (10 mL) and heated for 21 h at reflux. The mixture was first concentrated and then redissolved in EtOAc (10 mL) and acidified with 2 n HCl (20 mL), which lead to the formation of a precipitate that was separated. The phases of the mother liquor were separated, and the aqueous phase was basified with 2 n ammonia (20 mL) and extracted with EtOAc (3×50 mL). The combined extracts were dried over sodium sulfate and evaporated giving a first fraction of product. The formed precipitate was separated, dissolved in CH_2_Cl_2_ (100 mL) and extracted with 2 n ammonia (50 mL). The organic phase was dried over Na_2_SO_4_ and evaporated, giving a second fraction of product as a yellow oil (combined 353 mg, 57 %); ^1^H NMR (500 MHz, CDCl_3_): *δ*=1.05 (d, *J*=6.3 Hz, 6 H), 2.67 (dd, *J*=12.1, 7.7 Hz, 1 H), 2.74 (septet, *J*=6.3 Hz, 1 H) 2.83 (dd, *J*=12.0, 3.8 Hz, 1 H), 3.03 (t, *J*=7.7 Hz, 2 H), 3.33 (td, *J*=7.9, 1.8 Hz, 2 H), 3.95–4.01 (m, 1 H), 4.07 (d, *J*=5.0 Hz, 2 H), 6.96 (d, *J*=8.5 Hz, 1 H), 7.01 (t, *J*=7.6 Hz, 1 H), 7.18 (t, *J*=7.3 Hz, 1 H), 7.23 (d, *J*=7.6 Hz, 2 H), 7.28 (t, *J*=7.6 Hz, 2 H), 7.44 (t, *J*=7.9 Hz, 1 H), 7.66 ppm (d, *J*=7.6 Hz, 1 H); ^13^C NMR (125 MHz, CDCl_3_): *δ*=22.8/23.0, 30.2, 45.1, 48.9, 49.3, 68.1, 71.4, 113.0, 121.0, 125.9, 128.3 (2C), 128.4 (2C), 130.2, 133.4, 141.5, 157.6, 201.5 ppm; HRMS (ESI): calcd for C_21_H_28_NO_3_ *m*/*z* 342.2064 [*M*+H]^+^, found *m*/*z* 342.2070 [*M*+H]^+^.

The hydrochloride was prepared like for **5 a** and collected by filtration to yield a white solid (181 mg, 83 %); HRMS (ESI): calcd for C_21_H_28_NO_3_ *m*/*z* 342.2064 [*M*+H]^+^, found *m*/*z* 342.2068 [*M*+H]^+^.

**1‐(2‐(3‐((1‐Fluoropropan‐2‐yl)amino)‐2‐hydroxypropoxy)phenyl)‐3‐phenylpropan‐1‐one (16 b)**: The ketal **4 a** (501 mg, 1 eq, 1.46 mmol) and NaBH_3_CN (368 mg, 4 eq, 5.86 mmol) were dissolved in MeOH (10 mL), and fluoroacetone (357 mg, 3 eq, 4.69 mmol) and acetic acid (0.2 mL) were added. The mixture was heated for 4 h at reflux, then it was diluted with CH_2_Cl_2_ (40 mL) and with 2 n NaHCO_3_ solution (40 mL). The organic phase was dried over Na_2_SO_4_, filtered, and concentrated to yield a yellow oil (596 mg, quant., mixture of diasteromers) that was used without further purification. 1‐((1‐fluoropropan‐2‐yl)amino)‐3‐(2‐(2‐phenethyl‐1,3‐dioxolan‐2‐yl)phenoxy)‐propan‐2‐ol ^1^H NMR (500 MHz, CDCl_3_): *δ*=1.09 (t, *J*=7.4 Hz, 3 H), 2.44 (t, *J*=8.8 Hz, 2 H), 2.57–2.72 (m, 2 H), 2.79–2.91 (m, 2 H), 2.94–3.05 (m, 1 H), 3.81–3.93 (m, 2 H), 3.94–4.01 (m, 1 H), 4.01–4.15 (m, 3 H), 4.15–4.46 (m, 3 H), 6.90 (d, *J*=8.2 Hz, 1 H), 6.96 (t, *J*=7.6 Hz, 1 H), 7.09–7.16 (m, 1 H), 7.15 (d, *J*=7.6 Hz, 2 H), 7.23 (t, *J*=7.3 Hz, 2 H), 7.28 (t, *J*=7.5 Hz, 1 H), 7.48 ppm (d, *J*=7.6 Hz, 1 H); ^13^C NMR (125 MHz, CDCl_3_): *δ*=16.2 (d, ^3^J^13^C‐^19^F=7.7 Hz), 29.9, 39.9, 49.4, 53.1(d, ^2^J^13^C‐^19^F=18.4 Hz), 64.7, 64.8, 69.2, 72.7, 87.0/87.1 (d, ^1^J^13^C‐^19^F=168.5 Hz), 110.3, 114.4, 120.9, 125.6, 127.1, 128.2 (2C), 128.3 (2C), 129.6, 130.1, 142.1, 156.7 ppm; ^19^F NMR (470 MHz, CDCl_3_): *δ*=−224.5 (dt, *J*=47.3, 47.3, 16.9 Hz, 1F), −224.7 (dt, *J*=47.5, 47.5, 17.2 Hz, 1F).

The product from the last step (596 mg, 1 eq, 1.48 mmol) was dissolved in EtOAc (50 mL), and aq. HCl (2 n, 50 mL) was added and stirred for 1 h. Then the phases were separated, and the aqueous phase was extracted with EtOAc (2×60 mL). The combined organic phases were washed once with 2 n NaHCO_3_ solution (50 mL), dried over Na_2_SO_4_, and concentrated to a yellow oil (451 mg, 86 %, mixture of diasteromers); ^1^H NMR (500 MHz, CDCl_3_): *δ*=1.10 (t, *J*=7.1 Hz, 3 H), 2.79 (dd, *J*=12.0, 7.6 Hz, 1 H), 2.88–2.99 (m, 2 H), 3.03 (t, *J*=7.7 Hz, 2 H), 3.24–3.38 (m, 2 H), 3.99–4.14 (m, 3 H), 4.20–4.26 (m, 0.5 H), 4.29–4.35 (m, 1 H), 4.38–4.44 (m, 0.5 H), 6.97 (d, *J*=8.5 Hz, 1 H), 7.02 (t, *J*=7.3 Hz, 1 H), 7.19 (t, *J*=7.3 Hz, 1 H), 7.23 (d, *J*=7.3 Hz, 2 H), 7.28 (t, *J*=7.6 Hz, 2 H), 7.45 (t, *J*=7.7 Hz, 1 H), 7.67 ppm (d, *J*=7.9 Hz, 1 H); ^13^C NMR (125 MHz, CDCl_3_): *δ*=15.9/16.1 (d, ^3^J(^13^C‐^19^F)=6.1/7.7 Hz), 30.2, 44.9, 49.2, 53.1/53.2 (d, ^2^J(^13^C‐^19^F)=18.4 Hz), 68.0, 71.2/71.4, 86.4/86.5 (d, ^1^J(^13^C‐^19^F)=170.1/170.2 Hz), 113.1/113.2, 121.1, 126.0, 128.2/128.3, 128.4 (4C), 130.3, 133.5, 141.4, 157.6, 201.5 ppm; ^19^F NMR (470 MHz, CDCl_3_): *δ*=−225.2 (dt, *J*=47.5, 47.5, 17.7 Hz, 1F), −225.3 (dt, *J*=47.0, 47.0, 17.7 Hz, 1F); HRMS (ESI): calcd for C_21_H_27_FNO_3_ *m*/*z* 360.1969 [*M*+H]^+^, found *m*/*z* 360.1974 [*M*+H]^+^.

Hydrochloride formation like for **13 a** yielded a white solid (84 mg, 70 %); HRMS (ESI): calcd for C_21_H_27_FNO_3_ *m*/*z* 360.1969 [*M*+H]^+^, found *m*/*z* 360.1969 [*M*+H]^+^.

**1‐(2‐(3‐((1,1‐Difluoropropan‐2‐yl)amino)‐2‐hydroxypropoxy)phenyl)‐3‐phenylpropan‐1‐one(16 c)**: Reductive amination using 1,1‐difluoroacetone was performed like for **16 b** yielding a yellow oil (313 mg, quant., mixture of diastereomers), 1‐((1,1‐Difluoropropan‐2‐yl)amino)‐3‐(2‐(2‐phenethyl‐1,3‐dioxolan‐2‐yl)phenoxy)propan‐2‐ol ^1^H NMR (500 MHz, CDCl_3_): *δ*=1.16 (dd, *J*=6.3, 4.4 Hz, 3 H), 2.38–2.48 (m, 2 H), 2.56–2.72 (m, 2 H), 2.80–3.01 (m, 3 H), 3.82–3.92 (m, 2 H), 3.92–4.02 (m, 1 H), 4.02–4.14 (m, 3 H), 4.16–4.24 (m, 1 H), 5.63 (td, *J*=56.7, 4.0 Hz, 1 H), 6.90 (d, *J*=8.5 Hz, 1 H), 6.97 (t, *J*=7.6 Hz, 1 H), 7.09–7.18 (m, 3 H), 7.19–7.25 (m, 2 H), 7.28 (t, *J*=7.2 Hz, 1 H), 7.48 ppm (d, *J*=7.9 Hz, 1 H); ^13^C NMR (125 MHz, CDCl_3_): *δ*=13.6/14.0 (t, *J*=4.6 Hz/t, *J*=4.6 Hz), 29.9/30.0, 40.0, 49.5/49.7, 55.2/55.4 (t, *J*=21.6 Hz/t, *J*=23.1 Hz), 64.7/64.8, 69.1/69.3, 72.6, 110.3, 114.4, 118.0 (t, *J*=243.8 Hz), 121.0, 125.6, 127.1, 128.3 (4C), 129.6, 130.1, 142.1, 156.6 ppm; ^19^F NMR (470 MHz, CDCl_3_): *δ*=(*Diastereomer 1*) −124,4 (ddd, *J*=280.7, 56.8, 10.0 Hz, 1F), −127.9 (ddd, *J*=281.0, 56.7, 14.0 Hz, 1F), (*Diastereomer 2*) −125.0 (ddd, *J*=281.2, 56.5, 10.3 Hz, 1F), −127.3 (ddd, *J*=281.0, 56.5, 12.8 Hz, 1F).

Deprotection like for **13 b** yielded a yellow oil (232 mg, 84 %); ^1^H NMR (500 MHz, CDCl_3_): *δ*=1.13 (d, *J*=6.5 Hz, 3 H), 2.71–2.98 (m, 3 H), 3.03 (t, *J*=7.7 Hz, 2 H), 3.23–3.36 (m, 2 H), 3.92–4.00 (m, 1 H), 4.01–4.15 (m, 2 H), 5.59 (td, *J*=56.5, 3.8 Hz, 1 H)/5.60 (td, *J*=56.5, 3.8 Hz, 1 H), 6.96 (d, *J*=8.2 Hz, 1 H), 7.02 (t, *J*=7.6 Hz, 1 H), 7.20 (t, *J*=6.9 Hz, 1 H), 7.23 (d, *J*=7.6 Hz, 2 H), 7.28 (t, *J*=7.6 Hz, 2 H), 7.44 ppm (t, *J*=7.9 Hz, 1 H); ^13^C NMR (125 MHz, CDCl_3_): *δ*=13.8/14.0 (t, *J*=3.9 Hz/t, *J*=3.9 Hz), 30.2, 45.0, 49.6, 55.1 (t, *J*=22.4 Hz), 68.4/68.6, 71.3/71.4, 113.3, 117.6 (t, *J*=244.3 Hz), 121.1, 126.0, 128.3 (2C), 128.4 (2C), 130.0, 130.1, 133.4, 141.5, 157.6, 201.4 ppm; ^19^F NMR (470 MHz, CDCl_3_): *δ*=(*Diastereomer 1*) −125.6 (ddd, *J*=281.3, 56.7, 10.6 Hz, 1F), −127.6 (ddd, *J*=281.4, 56.4, 13.4 Hz, 1F), (*Diastereomer 2*) −125,6 (ddd, *J*=281.3, 56.7, 10.6 Hz, 1F), −127.5 (ddd, *J*=281.4, 56.4, 13.1 Hz, 1F); HRMS (ESI): calcd for C_21_H_26_F_2_NO_3_ *m*/*z* 378.1875 [*M*+H]^+^, found *m*/*z* 378.1878 [*M*+H]^+^.

Hydrochloride formation like for **13 a** yielded a white solid (123 mg, 53 %). HRMS (ESI): calcd for C_21_H_26_F_2_NO_3_ *m*/*z* 378.1875 [*M*+H]^+^, found *m*/*z* 378.1882 [*M*+H]^+^.

### In silico modeling

**BCRP model**: Data for building the BCRP inhibition prediction model was taken as‐is from Ref. 14a. The dataset contains 433 inhibitors and 545 noninhibitors. Model building is described in Ref. 27. Briefly, extended connectivity fingerprints (ECFP) were computed for each molecule of the training set using RDKit,^28^ with a bit vector length of 1024 and a radius of 4. Logistic regression was built in Python with the scikit‐learn library.^29^ The penalty and C parameters were optimized with GridSearch.

**P‐gp model**: Data for building the P‐gp inhibition prediction model was taken from Ref. 14b. It was curated using the procedure described in,^30^ and which can be summarized as follows: inorganic compounds were removed, salts were removed from mixtures, compounds containing rare atoms were removed, chemotypes were normalized using the clean 2 D, aromatize, mesomerize, neutralize, tautomerize, and all transform options, duplicated compounds were removed, and permanently charged compounds were removed. MOE 2011.10 and ChemAxon 5.11.3 software were used. The cleaned dataset contains 627 inhibitors and 553 noninhibitors of P‐gp. ECFP fingerprints were computed for each molecule of the training set using RDKit,^28^ with a bit vector length of 1024 and a radius of 4. Support Vector Machine (SVM) with RBF kernel was built in Python with the scikit‐learn library.^16^ The gamma and C parameters were optimized with GridSearch.

**Model validation**: Both models were validated by 10‐fold cross‐validation. Traditional confusion matrix‐related statistics are reported, as well as the area under the receiver operating characteristic (ROC) curve (AUC) that specifically evaluate the ranking capability of the models.

### In vitro inhibition studies

*Materials*: RPMI 1640 media for cell culture was purchased from LifeTechnologies (Rockville, MD, USA). Supplements for cell culture, including fetal bovine serum (FBS), the antibiotics penicillin and streptomycin, as well as vincristine for selection of CCRF VCR1000 cells were purchased from Sigma (St. Louis, MO, USA). The fluorescent substrates mitoxantrone and daunorubicin as well as verapamil, Ko143, DMSO, PBS (phosphate buffered saline), and all compounds used for HPMI buffer preparation were also purchased from Sigma.

*Cell culture*: The human myeloid leukemia PLB985 parental and stably expressing BCRP cell lines^31^ were kindly provided by B. Sarkadi (Institute of Enzymology, Research Centre for Natural Sciences, Budapest, Hungary) and K. Nemet (Creative Cell Ltd., Budapest, Hungary). Both cell lines were cultured in RPMI 1640 supplemented with 10 % FBS and 100 IU mL^−1^ of penicillin and streptomycin. The human T‐lymphoblast cell line CCRF VCR1000, overexpressing P‐gp, was obtained by stepwise selection of CCRF‐CEM in vincristine‐containing medium^32^ and was kindly provided by V. Gekeler (Altana‐Pharma formerly Byk‐Gulden, Konstanz, Germany). CCRF VCR1000 cells were cultured in RPMI 1640 medium containing 20 % FBS and were treated regularly with vincristine (1 μg mL^−1^) for about 72 h, followed by centrifugation (300 g, 5 min, RT) and resuspension in normal culture medium. All cell lines were maintained at 37 °C in an atmosphere containing 5 % CO_2_ with 95 % relative humidity.

*Steady‐state accumulation experiments*: For BCRP and P‐gp inhibition studies, the steady‐state accumulation of mitoxantrone (7 μm) and daunorubicin (3 μm), respectively, was performed as previously described.^33^ Briefly, cells were harvested, pelleted (300 g, 5 min, 4 °C), diluted to a concentration of 12×10^6^ cells mL^−1^ in HPMI (10 mm Hepes, 120 mm NaCl, 5 mm KCl, 0.4 mm MgCl_2_, 0.04 mm CaCl_2_, 10 mm glucose, 10 mm NaHCO_3_, 5 mm Na_2_HPO_4,_ pH 7.4 with NaOH) and kept on ice for further processing. For each data point, the cell suspension (25 μL) was preincubated for 5 min at 37 °C with 2 % DMSO (25 μL) in HPMI alone (DMSO control) or containing different concentrations of test compound solutions. Thereafter, cells were preloaded with daunorubicin (50 μL) or mitoxantrone (50 μL) for 30 or 20 min at 37 °C, respectively, to reach a final concentration of 3 or 7 μm, respectively. The final DMSO concentration did not exceed 1.5 %. Preloading was stopped by chilling cells on ice for 5 min followed by the addition of 400 μL ice‐cold PBS. Cells were pelleted (300 g, 5 min, 4 °C), the supernatant was discarded, and the cell pellet was resuspended in ice‐cold PBS (150 μL). Until measurement, cells were kept on ice and in the dark. Immediately before cell fluorescence measurement by flow cytometry (BD Accuri C6 and BD FACSCalibur, Becton Dickinson, San Jose, CA, USA; MACSQuant, MiltenyiBiotec GmbH, BergischGladbach, Germany), 50 μL of 4′,6‐diamidin‐2‐phenylindole solution (DAPI, 4 μg mL^−1^ in PBS) was added to gate out dead cells. 100 μm verapamil and 1 μm Ko143 were used as positive controls for P‐gp and BCRP inhibition, respectively.

*IC_50_ value measurements and calculations*: For compounds showing an inhibitory effect of 50 % inhibition compared with the positive control (100 μm verapamil for P‐gp and 1 μm Ko143 for BCRP) at a concentration of 100 μm, IC_50apparent_ values were estimated measuring at least eight different compound concentrations and performing nonlinear regression analyses (GraphPad Prism 6, “*log(Agonist)* vs. *response—Variable slope* ”), for which Equation (1) was used, where *X* is the log of compound concentration, *Y* is the response in fluorescent intensity units, “*Bottom*” and “*Top*” are the lower and the higher plateaus of the nonlinear fit curve, respectively, IC_50apparent_ refers to the IC_50apparent_ value, and HillSlope is a factor that describes the steepness of the curve.

(1) $$Y=\mathrm{𝐵𝑜𝑡𝑡𝑜𝑚}+\frac{\mathrm{𝑇𝑜𝑝}-\mathrm{𝐵𝑜𝑡𝑡𝑜𝑚}}{1+10^{\left(\log\left({\mathrm{IC}}_{50\mathrm{apparent}}\right)-X\right)\cdot \mathrm{Hillslope}}}$$

To correct for the expression‐level dependency of IC_50apparent_ values and the pump‐leak kinetics as reviewed in W. Stein,^34^ IC_50apparent_ values were calculated using Equation (2), where the fluorescence intensity at zero inhibitor concentration [*I*]_0_ is according the “*Bottom*” level and the fluorescence intensity at infinite inhibitor concentration [*I*]_∞_ is according to the “*Top*” level given by nonlinear regression analysis.^35^ At least three independent experiments were performed for each compound showing an IC_50_ value less than or equal to 10 μm.

(2) $${\mathrm{IC}}_{50}={\mathrm{IC}}_{50\mathrm{apparent}}\cdot \frac{\mathrm{fluorescence}\;\mathrm{intensity}\;\mathrm{at}\;{\left[I\right]}_{0}}{\mathrm{fluorescence}\;\mathrm{intensity}\;\mathrm{at}\;{\left[I\right]}_{\infty }}$$

## Supporting information

As a service to our authors and readers, this journal provides supporting information supplied by the authors. Such materials are peer reviewed and may be re‐organized for online delivery, but are not copy‐edited or typeset. Technical support issues arising from supporting information (other than missing files) should be addressed to the authors.

Supplementary
