# Supplementary material for: Subtle Structural Differences Trigger Inhibitory Activity of Propafenone Analogues at the Two Polyspecific ABC Transporters: P‐Glycoprotein (P‐gp) and Breast Cancer Resistance Protein (BCRP)
Source: ChemMedChem. 2016 Mar 10;11(12):1380–94. doi: 10.1002/cmdc.201500592 (PMC4949556; doi:10.1002/cmdc.201500592)
Supplement: Supplementary file 1 — Supplementary [file CMDC-11-1380-s001.pdf]

CHEMMEDCHEM
Chemistry & Drug Discovery

Supporting Information

Subtle Structural Differences Trigger Inhibitory Activity of Propafenone Analogues at the Two Polyspecific ABC Transporters: P-Glycoprotein (P-gp) and Breast Cancer Resistance Protein (BCRP)

Theresa Schwarz,[a] Floriane Montanari,[a] Anna Cseke,[a] Katrin Wlcek,[a] Lene Visvader,[a] Sarah Palme,[a] Peter Chiba,[b] Karl Kuchler,[c] Ernst Urban,[a] and Gerhard F. Ecker*[a]

cmdc_201500592_sm_miscellaneous_information.pdf

Supporting Information

Distances to the nearest neighbors in the training sets

For each newly synthesized compound, we retrieved the 5 nearest neighbors (NN) in the training set for the BCRP model and for the P-gp model. For this, similarity was measured by Tanimoto on RDKit fingerprints to each and every compound of the two training sets. The 5 compounds with highest Tanimoto similarity are the 5 nearest neighbors. We then averaged the 5 similarities and looked at the structure and activity of the neighbors. The results are summarized in Table S1 and S2 below.

Table S1: Similarity to the BCRP training set

Compound	*In vitro* activity	Prediction	Number of propafenones among the 5 NN	Average similarity	Number of inhibitors among the 5 NN
5c	20% [a]	inactive	5	0.69	0
5a	16.1 µM	inactive	5	0.81	0
10c	24%[a]	inactive	5	0.59	0
6a	5.8 µM	inactive	5	0.70	0
11c	37%[a]	inactive	5	0.56	0
6c	33%[a]	inactive	5	0.64	0
5b	3.1 µM	inactive	5	0.74	0
10a	3.3 µM	inactive	5	0.71	0
10b	2.9 µM	inactive	5	0.67	0
6b	3.8 µM	inhibitor	5	0.66	0
11b	2.3 µM	inhibitor	5	0.60	0
11a	21.5 µM	inactive	5	0.63	0
13a	53%[a]	inactive	5	0.95	0
13b	5.5 µM	inactive	5	0.92	0
13c	4.9 µM	inactive	5	0.89	0

[a] % inhibition rates are given for these compounds at a concentration of 100µM compared to the positive control Ko143 (1µM), which was set as 100% inhibition rate. Data are given as mean from 3 independent experiments with duplicate measurements

Table S2: Similarity to the P-gp training set

Compound	*In vitro* activity	Prediction	Number of propafenones among the 5 NN	Average similarity	Number of inhibitors among the 5 NN
5c	58.7 µM	inactive	5	0.71	5
5a	2.1 µM	inhibitor	5	0.81	5
10c	34.8 µM	inactive	5	0.62	5
6a	1.6 µM	inhibitor	5	0.70	5
11c	8%[a]	inactive	5	0.58	5
6c	4%[a]	inhibitor	5	0.65	5
5b	2.2 µM	inhibitor	5	0.75	5
10a	1.3 µM	inhibitor	5	0.74	5
10b	1.0 µM	inhibitor	5	0.68	5
6b	11 µM	inhibitor	5	0.67	5
11b	6.2 µM	inhibitor	5	0.62	5
11a	13.9 µM	inhibitor	5	0.65	5
13a	0.3 µM	inhibitor	5	0.96	5
13b	0.5 µM	inhibitor	5	0.93	5
13c	0.9 µM	inhibitor	5	0.90	5

[a] % inhibition rates are given for these compounds at a concentration of 100µM compared to the positive control verapamil (100µM), which was set as 100% inhibition rate. Data are given as mean from 3 independent experiments with triplicate measurements.

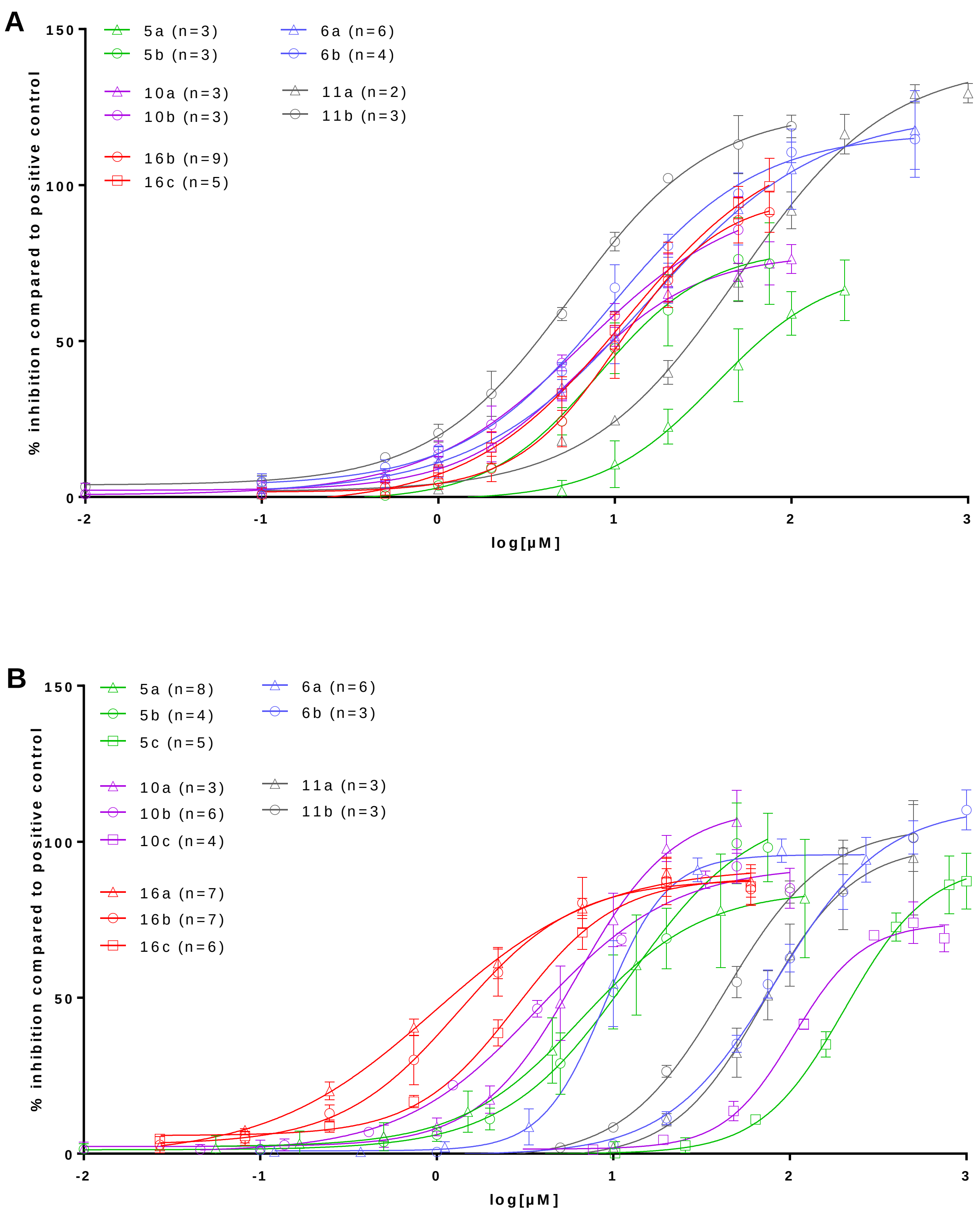

Figure S1: IC50 measurements for the compounds given in Table 2 in (A) BCRP expressing PLB cells and (B) P-gp overexpressing CCRF-VCR1000 cells. IC50 measurements were accomplished as described in the materials and methods section. Data given here show the mean percentage fluorescence intensity after subtracting the background fluorescence of unstained cells and the fluorescence of the DMSO control and subsequent normalization to the positive control Ko143 for BCRP and verapamil for P-gp, which were set as 100%, ± SD of at least 3 independent experiments. Compound 11a was measured in 2 independent

experiments for inhibition of BCRP. Non-linear regression analyses were performed as described in the materials and methods section.

Table S3: Hill coefficients of IC50 measurements for the compounds given in Table 2. Data are given as mean ± SD of n experiments

	Hill coefficient	
**Compound**	**P-gp (n)**	**BCRP (n)**
5a	1.3±0.2 (8)	1.4±0.3 (3)
5b	1.3±0.2 (4)	1.4±0.1 (3)
5c	1.9±0.2 (5)	-
6a	2.6±0.4 (6)	0.9±0.1 (6)
6b	1.5±0.2 (3)	1.1±0.2 (4)
6c	-	-
10a	1.6±0.2 (3)	1.3±0.2 (3)
10b	1.1±0.1 (6)	1.0±0.1 (3)
10c	2.2±0.4 (4)	-
11a	1.8±0.4 (3)	1.0±0.0 (2)
11b	1.5±0.1 (3)	1.1±0.2 (3)
11c	-	-
16a	0.9±0.1 (7)	-
16b	1.3±0.2 (7)	1.6±0.2 (9)
16c	1.5±0.1 (6)	1.0±0.2 (5)

-, no IC50 measurements accomplished

Purity of target compounds was defined by 13C-NMR (absence of additional signals).

5a

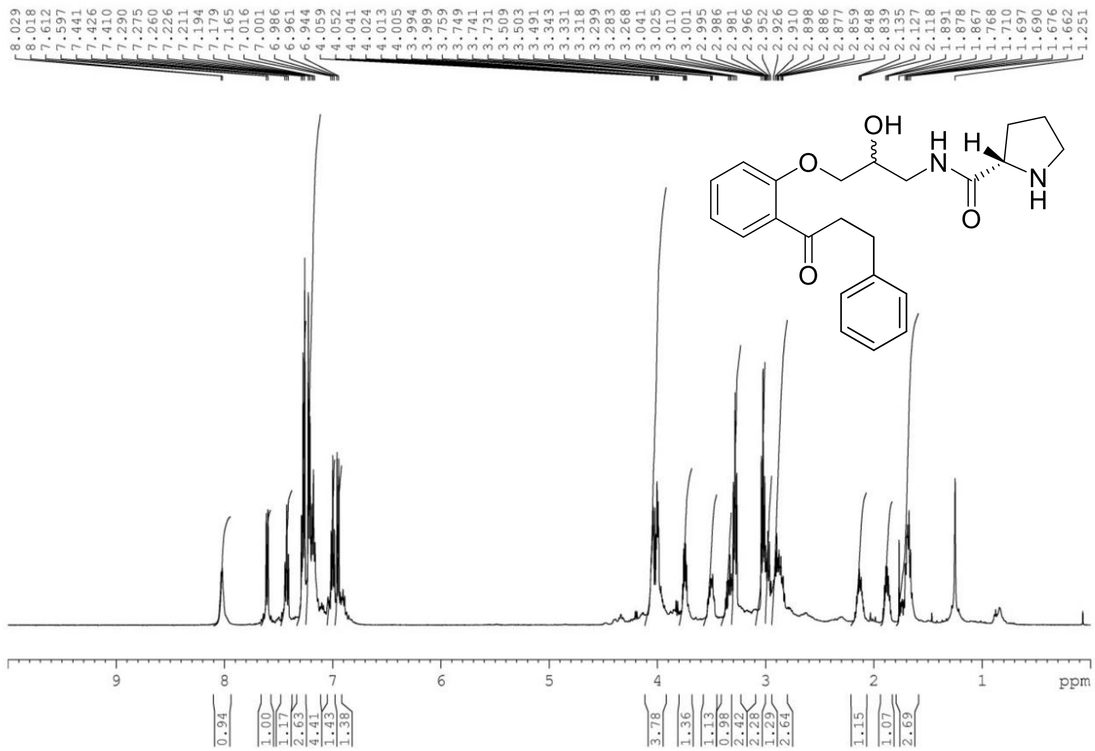

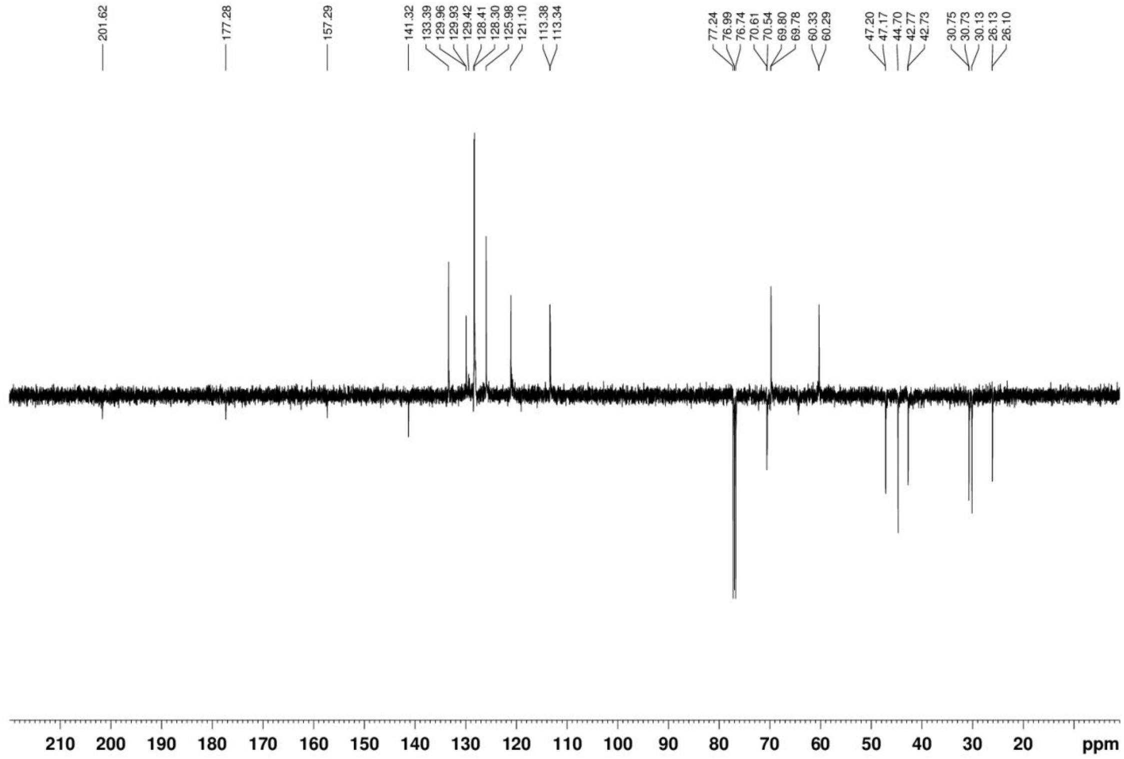

5b

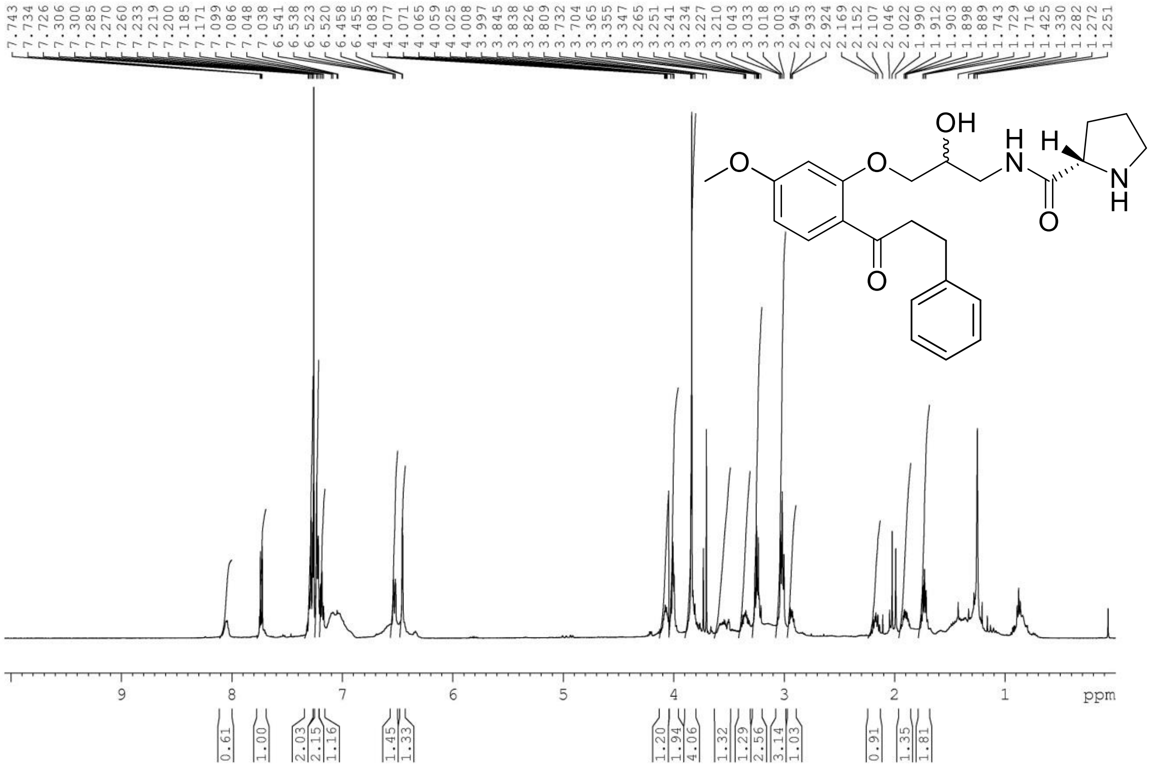
7.743 7.734 7.726 7.306 7.300 7.285 7.270 7.260 7.233 7.219 7.200 7.185 7.171 7.099 7.086 7.048 7.038 6.541 6.538 6.523 6.520 6.458 6.455 4.083 4.077 4.071 4.065 4.059 4.025 4.008 3.997 3.845 3.838 3.826 3.809 3.732 3.704 3.365 3.355 3.347 3.265 3.251 3.241 3.234 3.227 3.210 3.043 3.033 3.018 3.003 2.945 2.933 2.924 2.169 2.152 2.107 2.046 2.022 1.990 1.912 1.903 1.898 1.889 1.743 1.729 1.716 1.425 1.330 1.282 1.272 1.251
OH
H
N
H
O
O
NH
H
O
9 8 7 6 5 4 3 2 1 ppm
0.61 1.00 2.03 2.15 1.16 1.45 1.33 1.20 1.94 4.06 1.32 1.29 2.56 3.14 1.03 0.91 1.35 1.81

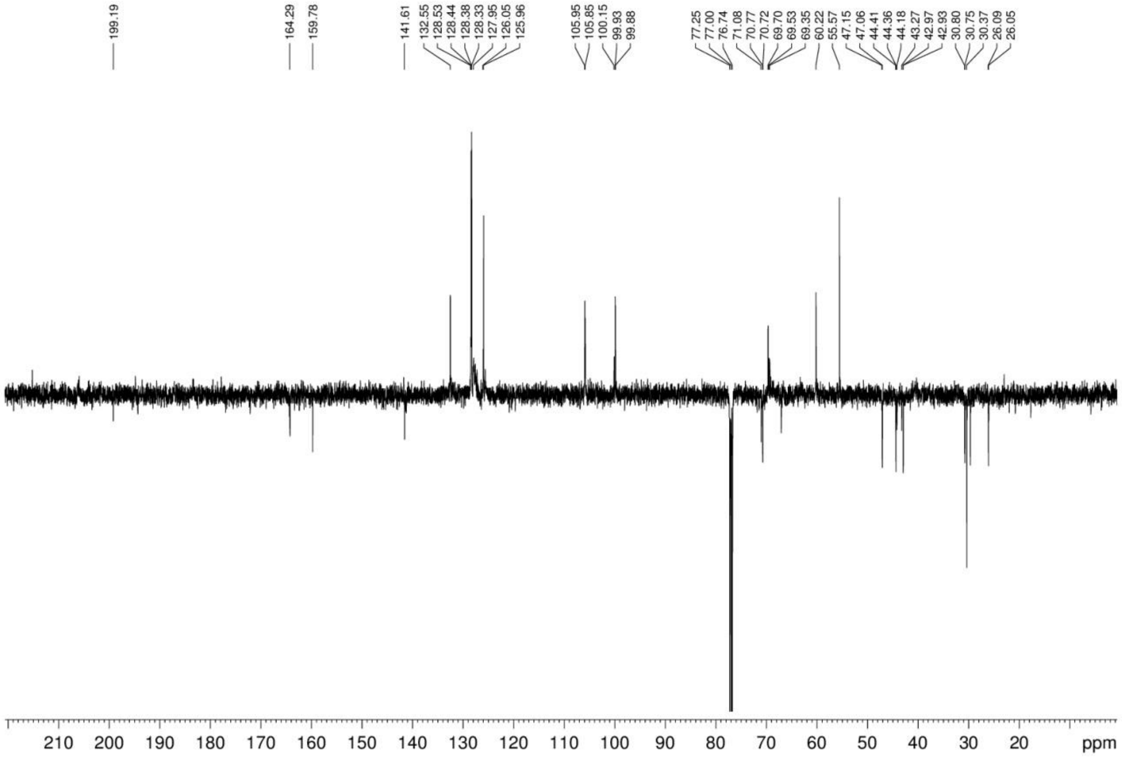
199.19
164.29 159.78
141.61 132.55 128.53 128.44 128.38 128.33 127.95 126.05 125.96
105.95 105.85 100.15 99.93 99.88
77.25 77.00 76.74 71.08 70.77 70.72 69.70 69.53 69.35 60.22 55.57 47.15 47.06 44.41 44.36 44.18 43.27 42.97 42.93 30.80 30.75 30.37 26.09 26.05
210 200 190 180 170 160 150 140 130 120 110 100 90 80 70 60 50 40 30 20 ppm

5c

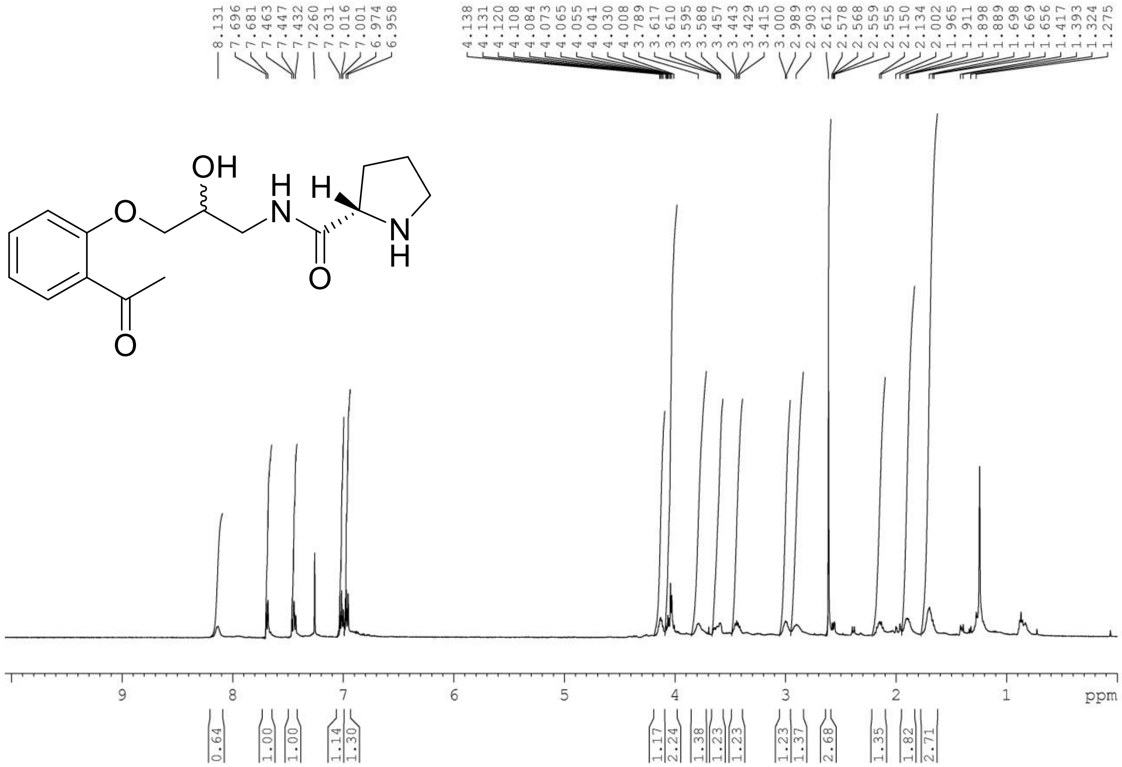
8.131
7.696
7.681
7.463
7.447
7.432
7.260
7.031
7.016
7.001
6.974
6.958
4.138
4.131
4.120
4.108
4.084
4.073
4.065
4.055
4.041
4.030
4.008
3.789
3.617
3.610
3.595
3.588
3.457
3.443
3.429
3.415
3.000
2.989
2.903
2.612
2.578
2.568
2.559
2.555
2.150
2.134
2.002
1.965
1.911
1.898
1.889
1.698
1.669
1.656
1.417
1.393
1.324
1.275
OH
H
N
O
H
N
H
O
9
8
7
6
5
4
3
2
1
ppm
0.64
1.00
1.00
1.14
1.30
1.17
2.24
1.38
1.23
1.23
1.23
1.37
2.68
1.35
1.82
2.71

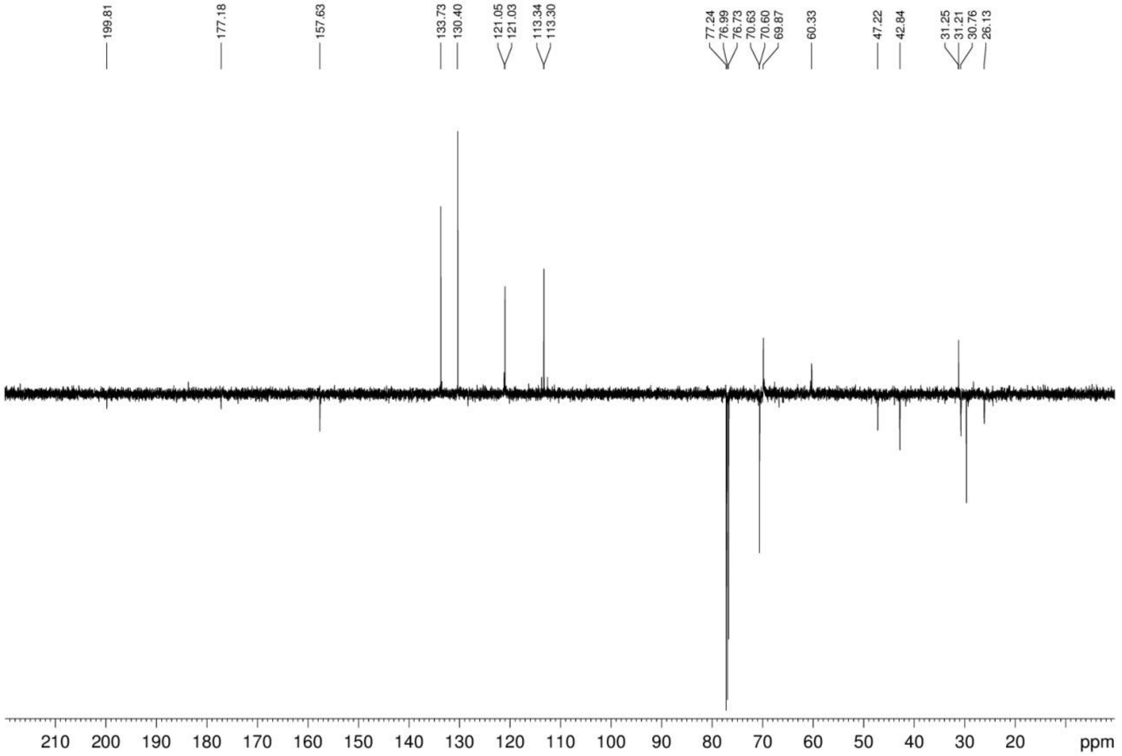
199.81
177.18
157.63
133.73
130.40
121.05
121.03
113.34
113.30
77.24
76.99
76.73
70.63
70.60
69.87
60.33
47.22
42.84
31.25
31.21
30.76
26.13
210 200 190 180 170 160 150 140 130 120 110 100 90 80 70 60 50 40 30 20 ppm

6a

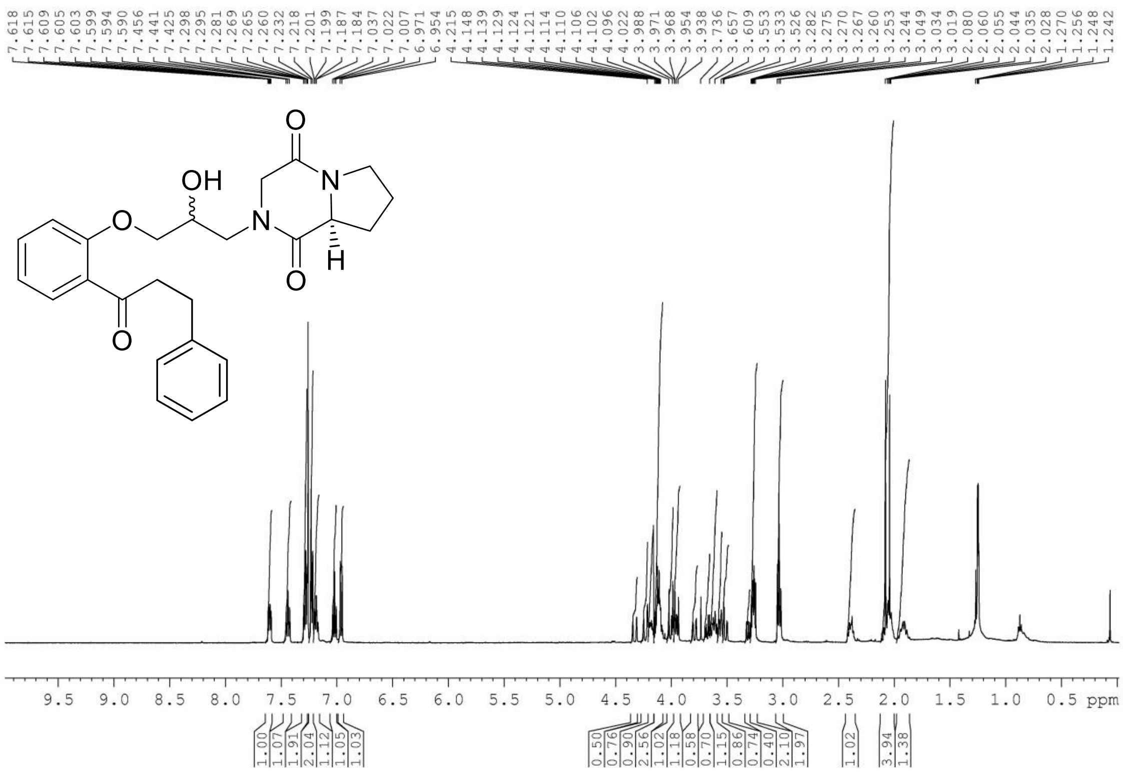
7.618 7.615 7.609 7.605 7.603 7.599 7.594 7.590 7.456 7.441 7.425 7.298 7.295 7.281 7.269 7.265 7.260 7.232 7.218 7.201 7.199 7.187 7.184 7.037 7.022 7.007 6.971 6.954 4.215 4.148 4.139 4.129 4.124 4.121 4.114 4.110 4.106 4.102 4.096 4.022 3.988 3.971 3.968 3.954 3.938 3.736 3.657 3.609 3.553 3.533 3.526 3.282 3.275 3.270 3.267 3.260 3.253 3.244 3.049 3.034 3.019 2.080 2.060 2.055 2.044 2.035 2.028 1.270 1.256 1.248 1.242
O
OH
N
O
N
H
O
O
9.5 9.0 8.5 8.0 7.5 7.0 6.5 6.0 5.5 5.0 4.5 4.0 3.5 3.0 2.5 2.0 1.5 1.0 0.5 ppm
1.00 1.07 1.91 2.04 1.12 1.05 1.03 0.50 0.76 0.90 2.56 1.02 1.18 0.58 0.70 1.15 0.86 0.74 0.40 2.10 1.97 1.02 3.94 1.38

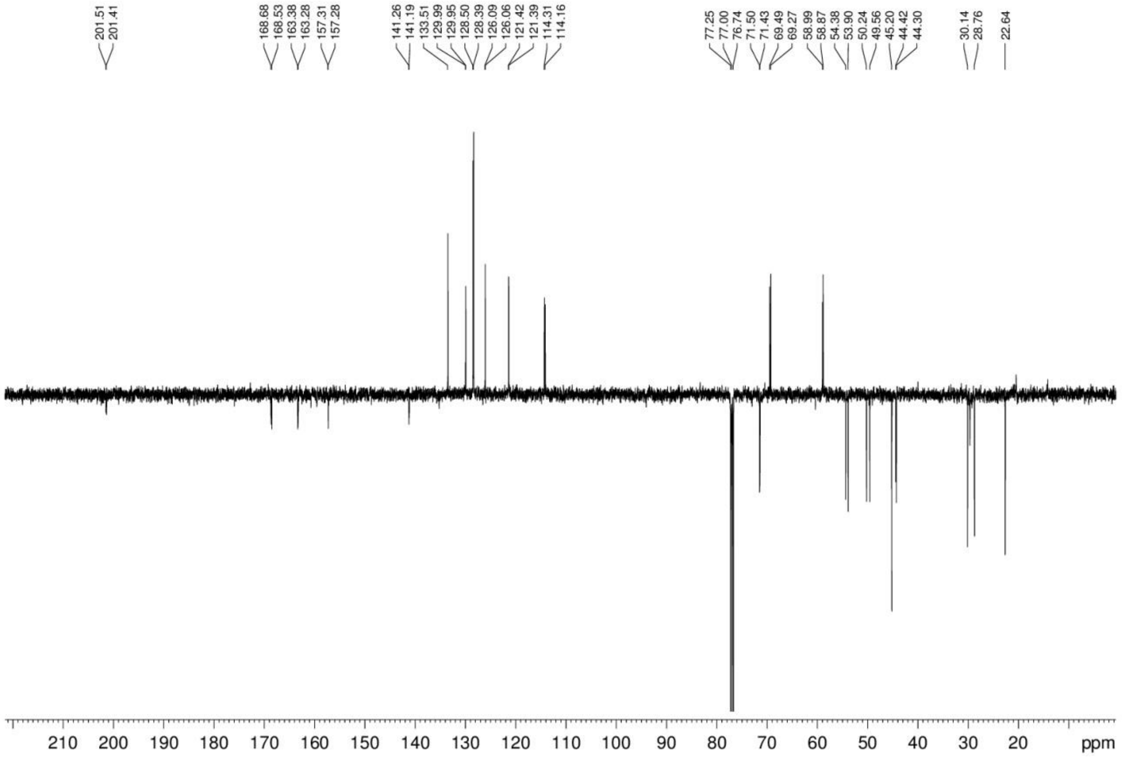
201.51 201.41 168.68 168.53 163.38 163.28 157.31 157.28 141.26 141.19 133.51 129.99 129.95 128.50 128.39 126.09 126.06 121.42 121.39 114.31 114.16 77.25 77.00 76.74 71.50 71.43 69.49 69.27 58.99 58.87 54.38 53.90 50.24 49.56 45.20 44.42 44.30 30.14 28.76 22.64
210 200 190 180 170 160 150 140 130 120 110 100 90 80 70 60 50 40 30 20 ppm

6b

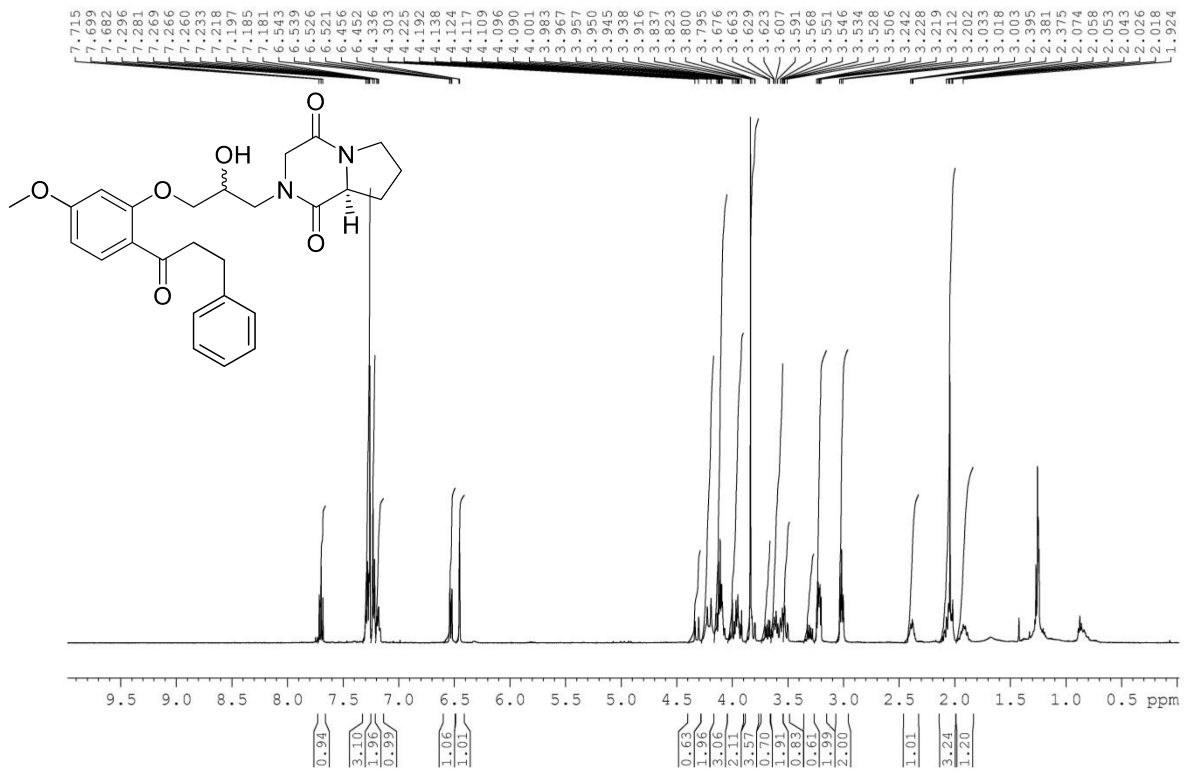
7.715 7.699 7.682 7.296 7.281 7.269 7.266 7.260 7.233 7.218 7.197 7.185 7.181 6.543 6.539 6.526 6.521 6.456 6.452 4.336 4.303 4.225 4.192 4.138 4.124 4.117 4.109 4.096 4.090 4.001 3.983 3.967 3.957 3.950 3.945 3.938 3.916 3.837 3.823 3.800 3.795 3.676 3.663 3.629 3.623 3.607 3.591 3.568 3.551 3.546 3.534 3.528 3.506 3.242 3.228 3.219 3.212 3.202 3.033 3.018 3.003 2.395 2.381 2.375 2.074 2.058 2.053 2.043 2.026 2.018 1.924
OH
O
N
H
9.5 9.0 8.5 8.0 7.5 7.0 6.5 6.0 5.5 5.0 4.5 4.0 3.5 3.0 2.5 2.0 1.5 1.0 0.5 ppm
0.94 3.10 1.96 0.99 1.06 1.01 0.63 1.96 3.06 2.11 3.57 0.70 1.91 0.83 0.61 1.99 2.00 1.01 3.24 1.20

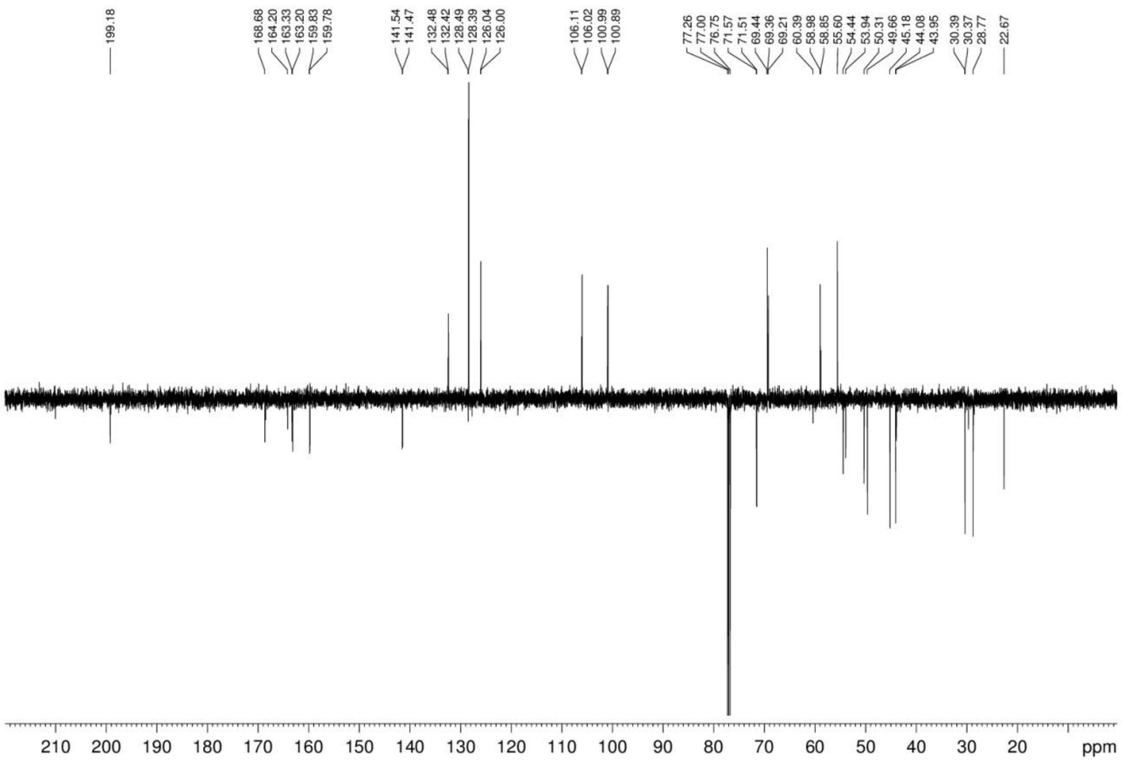
199.18
168.68 164.20 163.33 163.20 159.83 159.78
141.54 141.47 132.48 132.42 128.49 128.39 126.04 126.00
106.11 106.02 100.99 100.89
77.26 77.00 76.75 71.57 71.51 69.44 69.36 69.21 60.39 58.98 58.85 55.60 54.44 53.94 50.31 49.66 45.18 44.08 43.95 30.39 30.37 28.77 22.67
210 200 190 180 170 160 150 140 130 120 110 100 90 80 70 60 50 40 30 20 ppm

6c

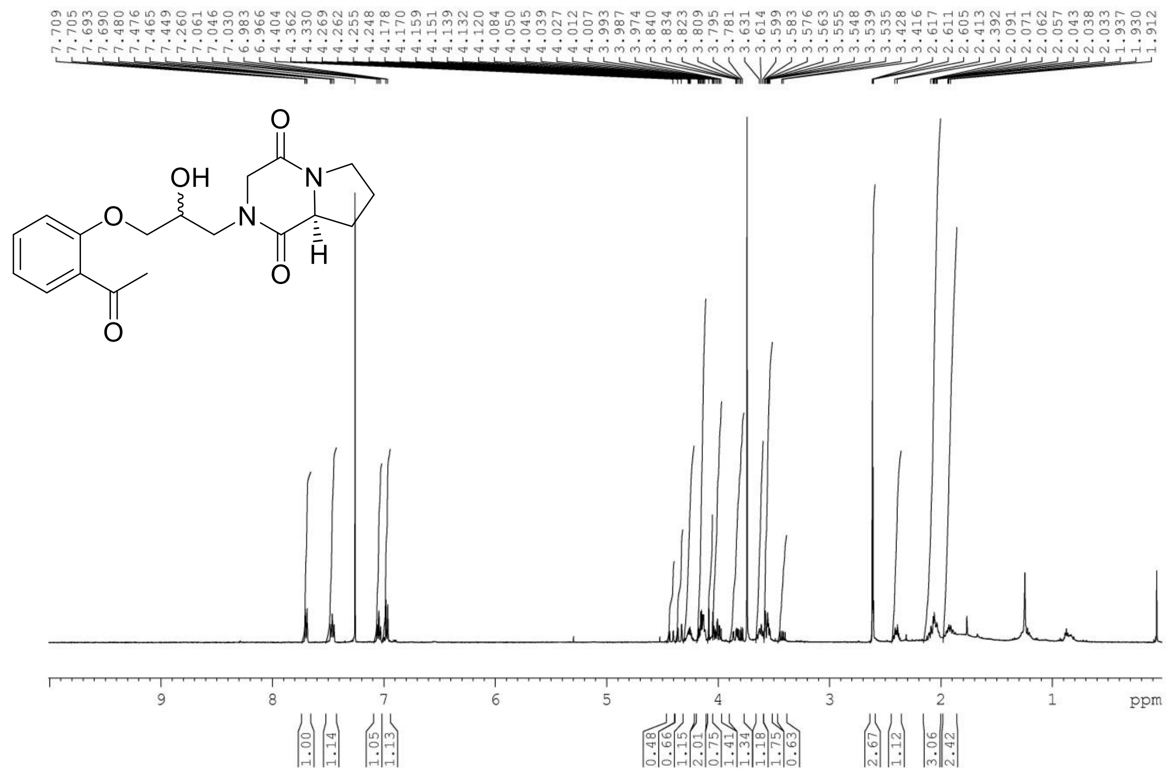
7.709
7.705
7.693
7.690
7.480
7.476
7.465
7.449
7.260
7.061
7.046
7.030
6.983
6.966
4.404
4.362
4.330
4.269
4.262
4.255
4.248
4.178
4.170
4.159
4.151
4.139
4.132
4.120
4.084
4.050
4.045
4.039
4.027
4.012
4.007
3.993
3.987
3.974
3.840
3.834
3.823
3.809
3.795
3.781
3.631
3.614
3.599
3.583
3.576
3.563
3.555
3.548
3.539
3.535
3.428
3.416
2.617
2.611
2.605
2.413
2.392
2.091
2.071
2.062
2.057
2.043
2.038
2.033
1.937
1.930
1.912
OH
O
N
N
O
O
H
O
9
8
7
6
5
4
3
2
1
ppm
1.00
1.14
1.05
1.13
0.48
0.66
1.15
2.01
0.75
1.41
1.34
1.18
1.75
0.63
2.67
1.12
3.06
2.42

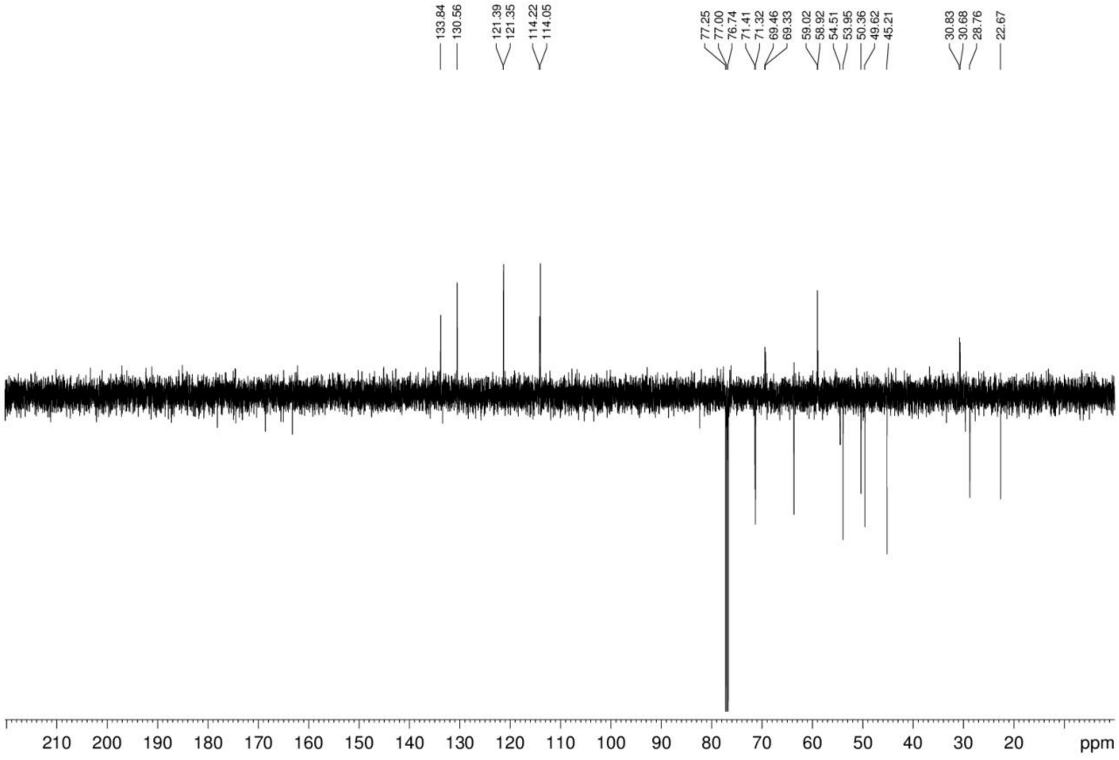
133.84
130.56
121.39
121.35
114.22
114.05
77.25
77.00
76.74
71.41
71.32
69.46
69.33
59.02
58.92
54.51
53.95
50.36
49.62
45.21
30.83
30.68
28.76
22.67
210
200
190
180
170
160
150
140
130
120
110
100
90
80
70
60
50
40
30
20
ppm

10a

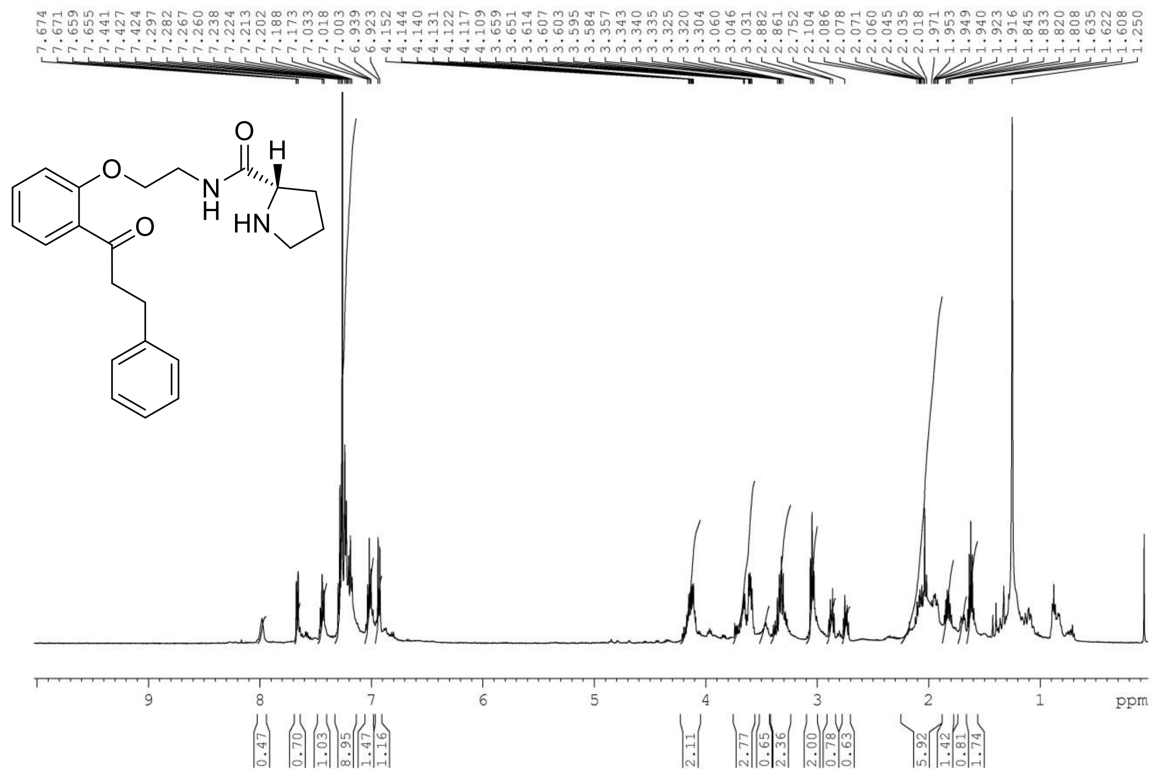
HN
ppm

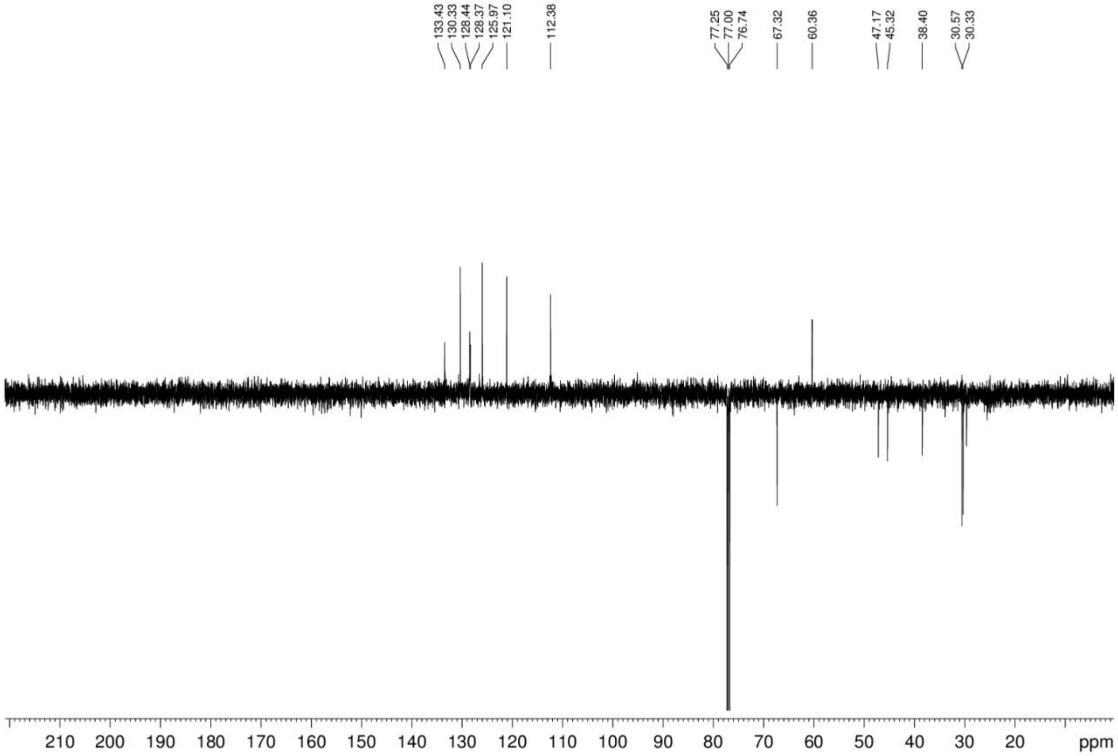
133.43
130.33
128.44
128.37
125.97
121.10
112.38
77.25
77.00
76.74
67.32
60.36
47.17
45.32
38.40
30.57
30.33
ppm

10b

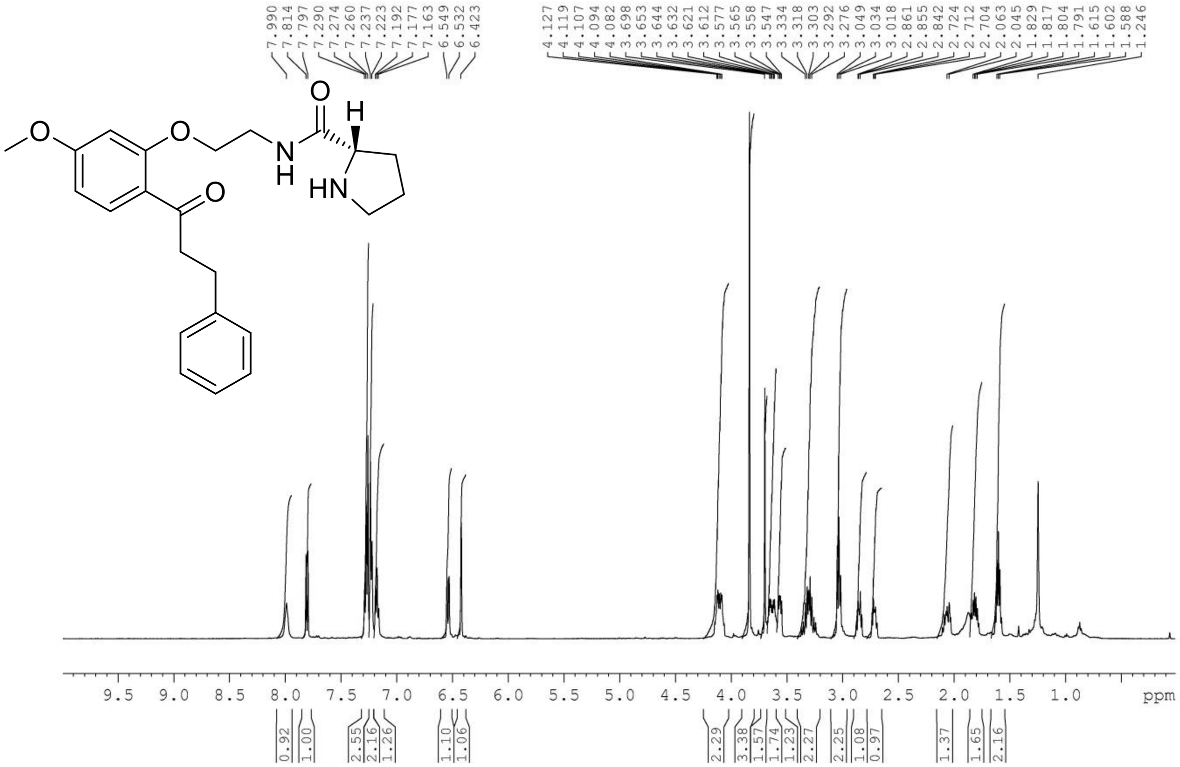
O
O
N
H
O
H
HN
O
7.990 7.814 7.797 7.290 7.274 7.260 7.237 7.223 7.192 7.177 7.163 6.549 6.532 6.423
4.127 4.119 4.107 4.094 4.082 3.698 3.653 3.644 3.632 3.621 3.612 3.577 3.565 3.558 3.547 3.334 3.318 3.303 3.292 3.276 3.049 3.034 3.018 2.861 2.855 2.842 2.724 2.712 2.704 2.063 2.045 1.829 1.817 1.804 1.791 1.615 1.602 1.588 1.246
9.5 9.0 8.5 8.0 7.5 7.0 6.5 6.0 5.5 5.0 4.5 4.0 3.5 3.0 2.5 2.0 1.5 1.0 ppm
0.92 1.00 2.55 2.16 1.26 1.10 1.06 2.29 3.38 1.57 1.74 1.23 2.27 2.25 1.08 0.97 1.37 1.65 2.16

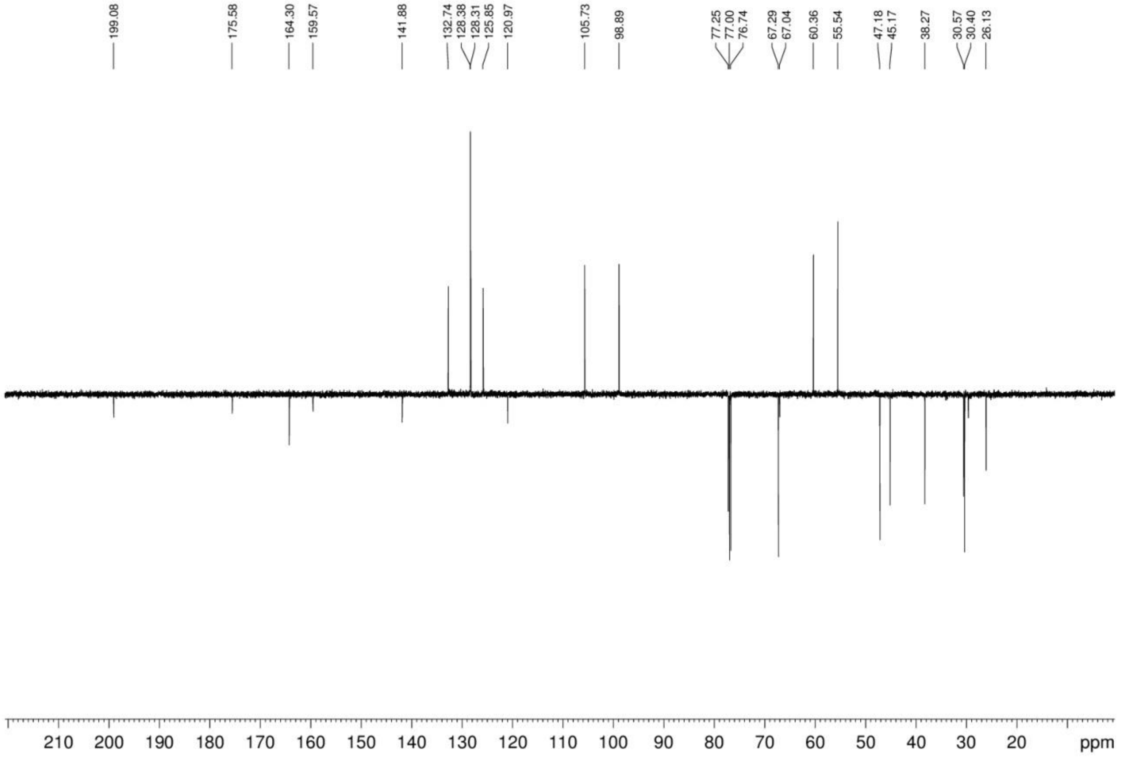
199.08 175.58 164.30 159.57 141.88 132.74 128.38 128.31 125.85 120.97 105.73 98.89 77.25 77.00 76.74 67.29 67.04 60.36 55.54 47.18 45.17 38.27 30.57 30.40 26.13
210 200 190 180 170 160 150 140 130 120 110 100 90 80 70 60 50 40 30 20 ppm

10c

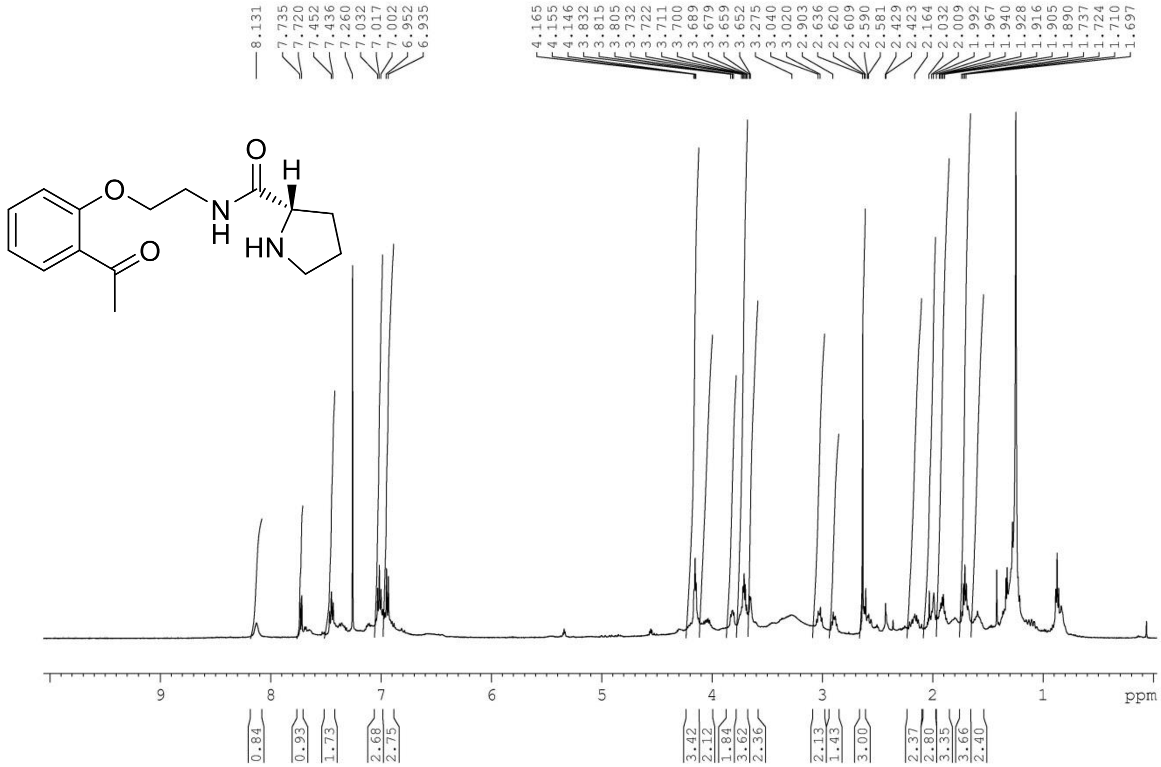
8.131
7.735
7.720
7.452
7.436
7.260
7.032
7.017
7.002
6.952
6.935
4.165
4.155
4.146
3.832
3.815
3.805
3.732
3.722
3.711
3.700
3.689
3.679
3.659
3.652
3.275
3.040
3.020
2.903
2.636
2.620
2.609
2.590
2.581
2.429
2.423
2.164
2.032
2.009
1.992
1.967
1.940
1.928
1.916
1.905
1.890
1.737
1.724
1.710
1.697
O
H
O
N
H
HN
O
9
8
7
6
5
4
3
2
1
ppm
0.84
0.93
1.73
2.68
2.75
3.42
2.12
1.84
3.62
2.36
2.13
1.43
3.00
2.37
2.80
3.35
3.66
2.40

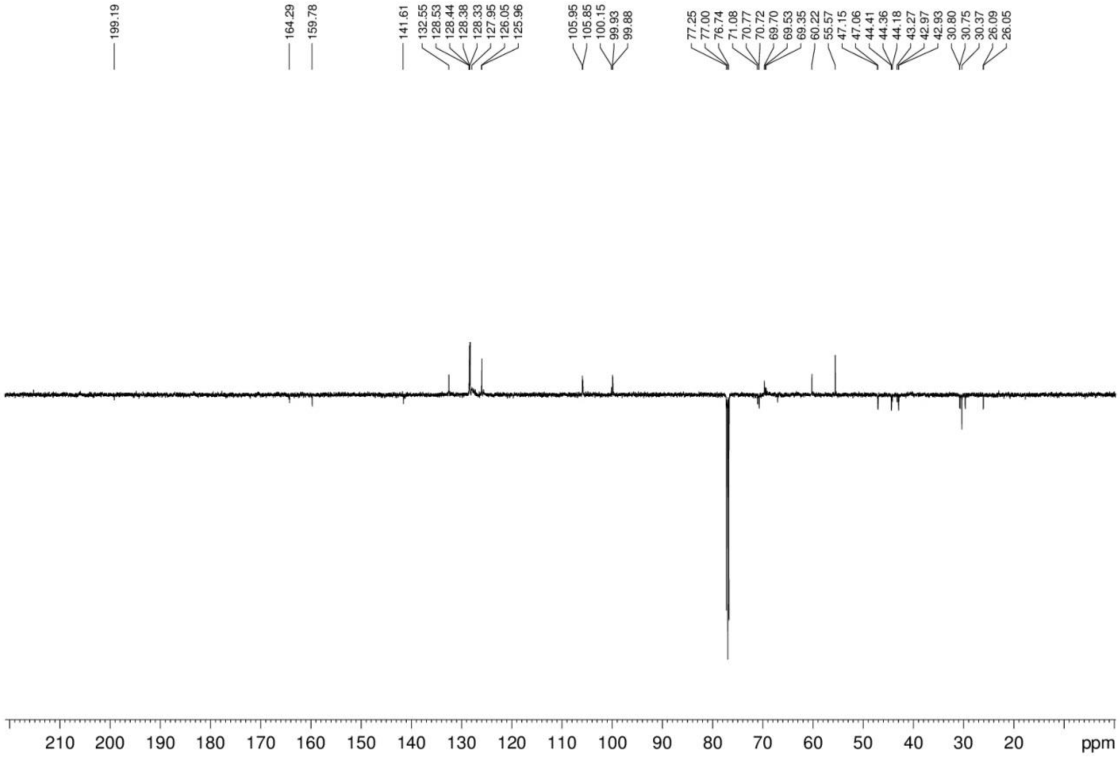
199.19
164.29
159.78
141.61
132.55
128.53
128.44
128.38
128.33
127.95
126.05
125.96
105.95
105.85
100.15
99.93
99.88
77.25
77.00
76.74
71.08
70.77
70.72
69.70
69.53
69.35
60.22
55.57
47.15
47.06
44.41
44.36
44.18
43.27
42.97
42.93
30.80
30.75
30.37
26.09
26.05
210
200
190
180
170
160
150
140
130
120
110
100
90
80
70
60
50
40
30
20
ppm

11a

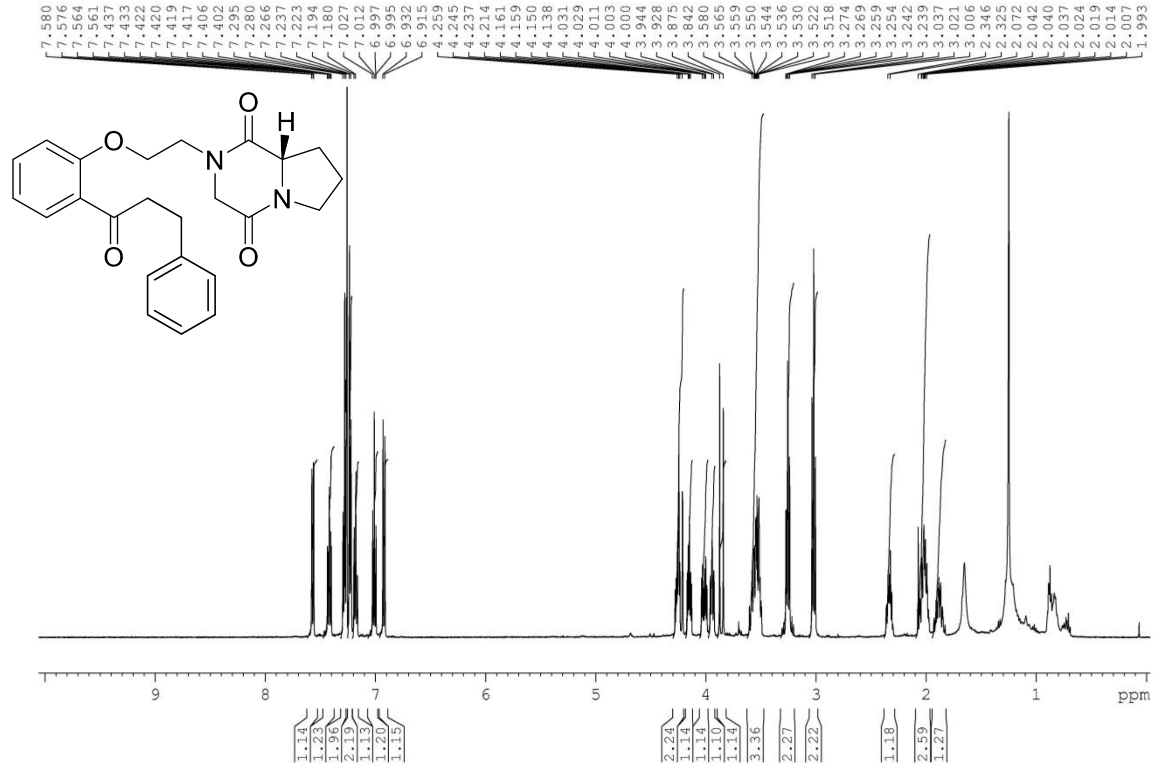
7.580 7.576 7.564 7.561 7.437 7.433 7.422 7.420 7.419 7.417 7.406 7.402 7.295 7.280 7.266 7.237 7.223 7.194 7.180 7.027 7.012 6.997 6.995 6.932 6.915 4.259 4.245 4.237 4.214 4.161 4.159 4.150 4.138 4.031 4.029 4.011 4.003 4.000 3.944 3.928 3.875 3.842 3.580 3.565 3.559 3.550 3.544 3.536 3.530 3.522 3.518 3.274 3.269 3.259 3.254 3.242 3.239 3.037 3.021 3.006 2.346 2.325 2.072 2.042 2.040 2.037 2.024 2.019 2.014 2.007 1.993
O
H
N
N
9
8
7
6
5
4
3
2
1
ppm
1.14 1.23 1.96 2.19 1.13 1.20 1.15
2.24 1.14 1.14 1.10 1.14 3.36 2.27 2.22
1.18 2.59 1.27

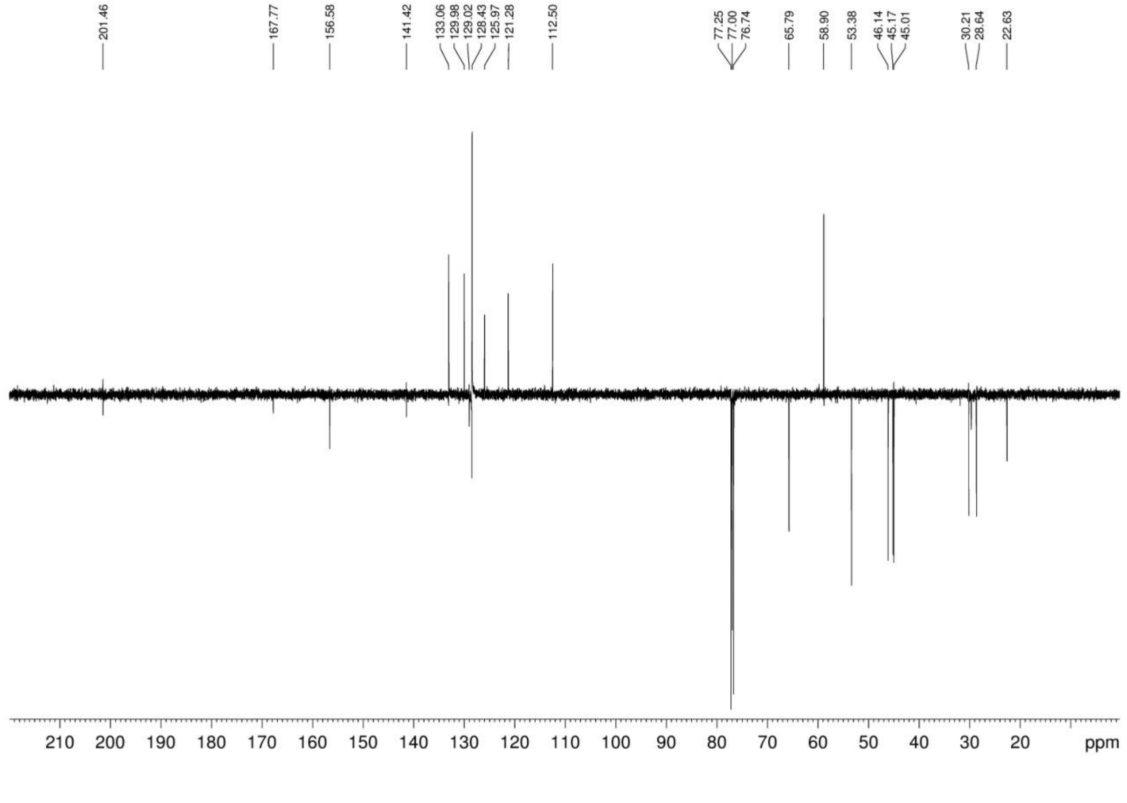
201.46
167.77
156.58
141.42
133.06 129.98 129.02 128.43 125.97 121.28
112.50
77.25 77.00 76.74
65.79
58.90
53.38
46.14 45.17 45.01
30.21 28.64
22.63
210 200 190 180 170 160 150 140 130 120 110 100 90 80 70 60 50 40 30 20 ppm

11b

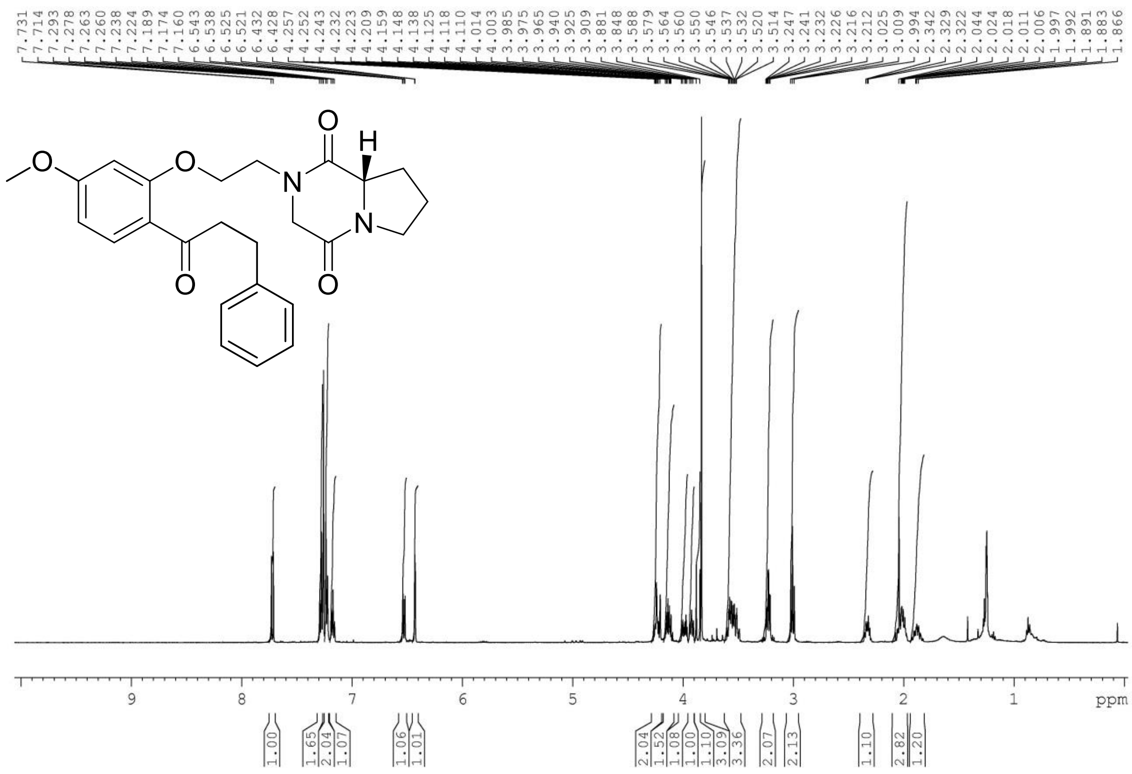

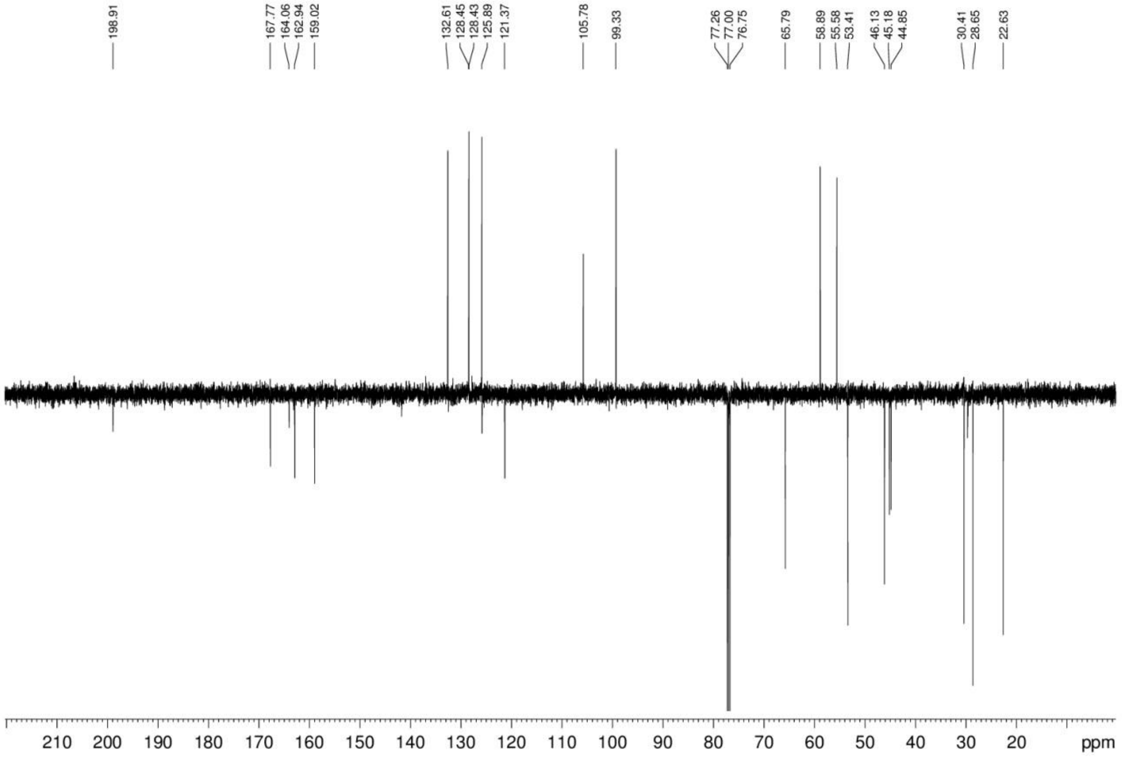

11c

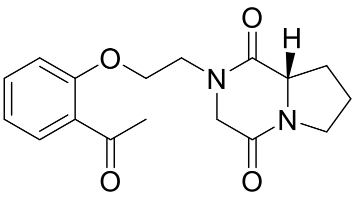

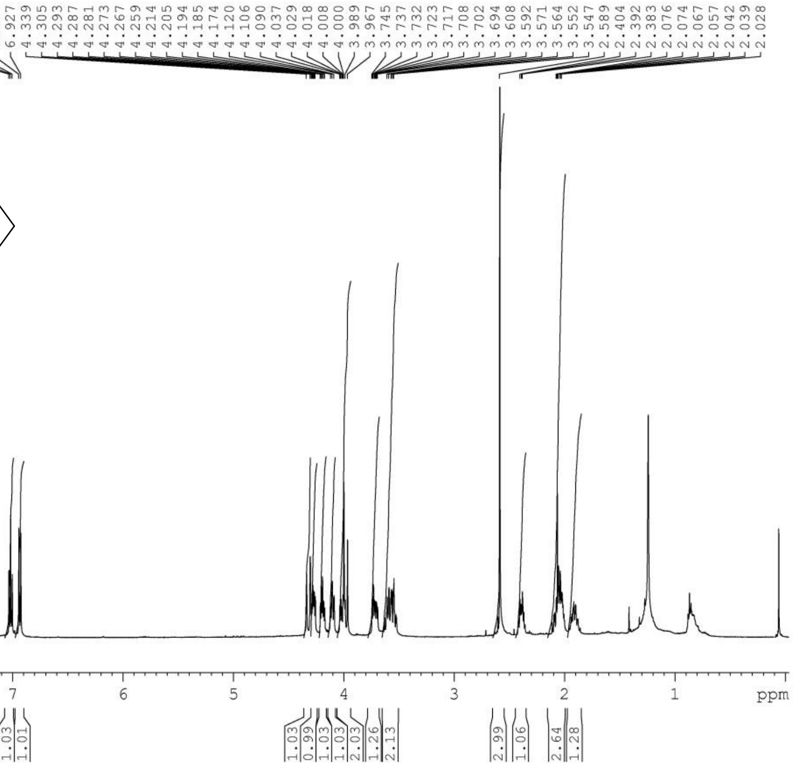
7.672 7.668 7.656 7.653 7.454 7.450 7.436 7.422 7.419 7.262 7.260 7.034 7.019 7.005 6.944 6.927 4.339 4.305 4.293 4.287 4.281 4.273 4.267 4.259 4.214 4.205 4.194 4.185 4.174 4.120 4.106 4.090 4.037 4.029 4.018 4.008 4.000 3.989 3.967 3.745 3.737 3.732 3.723 3.717 3.708 3.702 3.694 3.608 3.592 3.571 3.564 3.552 3.547 2.589 2.404 2.392 2.383 2.076 2.074 2.067 2.057 2.042 2.039 2.028
9 8 7 6 5 4 3 2 1 ppm
1.00 1.04 1.03 1.01 1.03 0.99 1.03 1.03 2.03 2.03 1.26 2.13 2.99 1.06 2.64 1.28

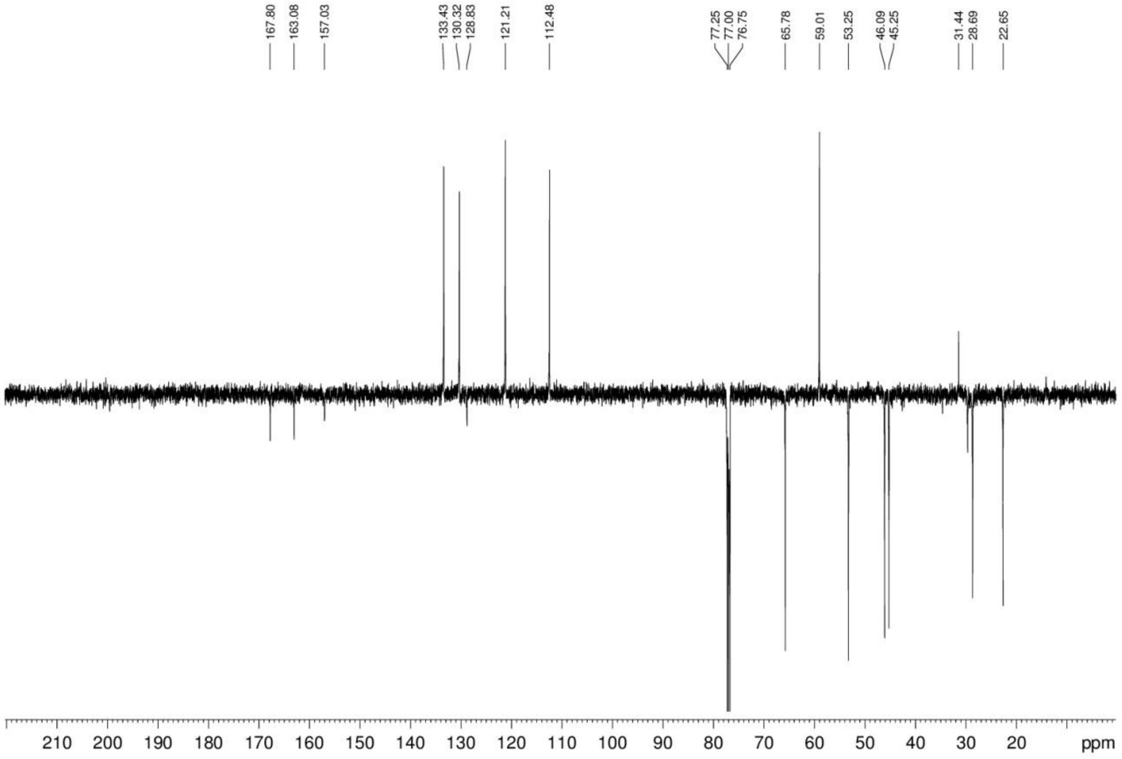
167.80 163.08 157.03 133.43 130.32 128.83 121.21 112.48 77.25 77.00 76.75 65.78 59.01 53.25 46.09 45.25 31.44 28.69 22.65
210 200 190 180 170 160 150 140 130 120 110 100 90 80 70 60 50 40 30 20 ppm

16a

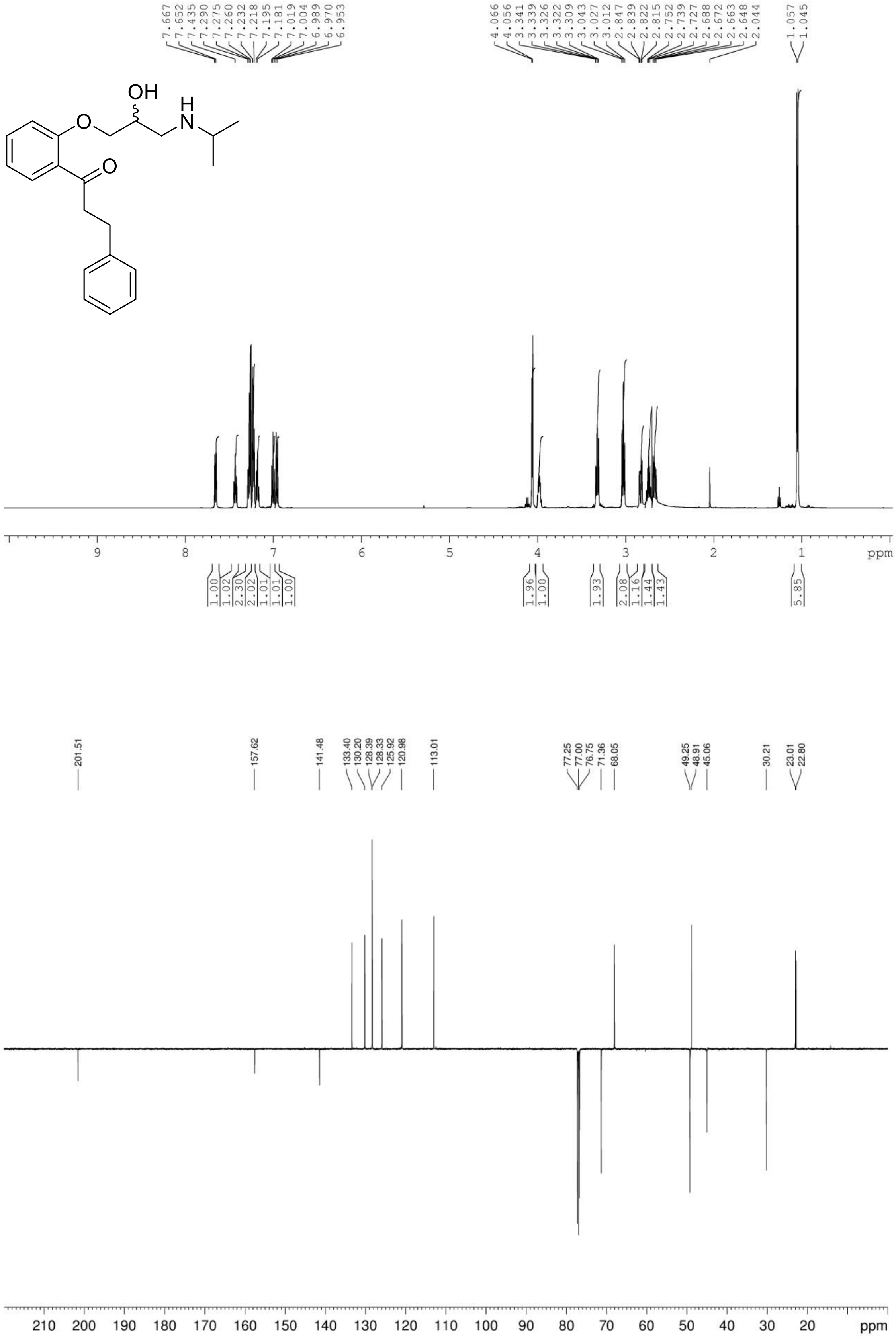
OH
H
N
O
O
7.667
7.652
7.435
7.290
7.275
7.260
7.232
7.218
7.195
7.181
7.019
7.004
6.989
6.970
6.953
4.066
4.056
3.341
3.339
3.326
3.322
3.309
3.043
3.027
3.012
2.847
2.839
2.822
2.815
2.752
2.739
2.727
2.688
2.672
2.663
2.648
2.044
1.057
1.045
9 8 7 6 5 4 3 2 1 ppm
1.00
1.02
2.30
2.02
1.01
1.01
1.00
1.96
1.00
1.93
2.08
1.16
1.44
1.43
5.85
201.51
157.62
141.48
133.40
130.20
128.39
128.33
125.92
120.98
113.01
77.25
77.00
76.75
71.36
68.05
49.25
48.91
45.06
30.21
23.01
22.80
210 200 190 180 170 160 150 140 130 120 110 100 90 80 70 60 50 40 30 20 ppm

16b

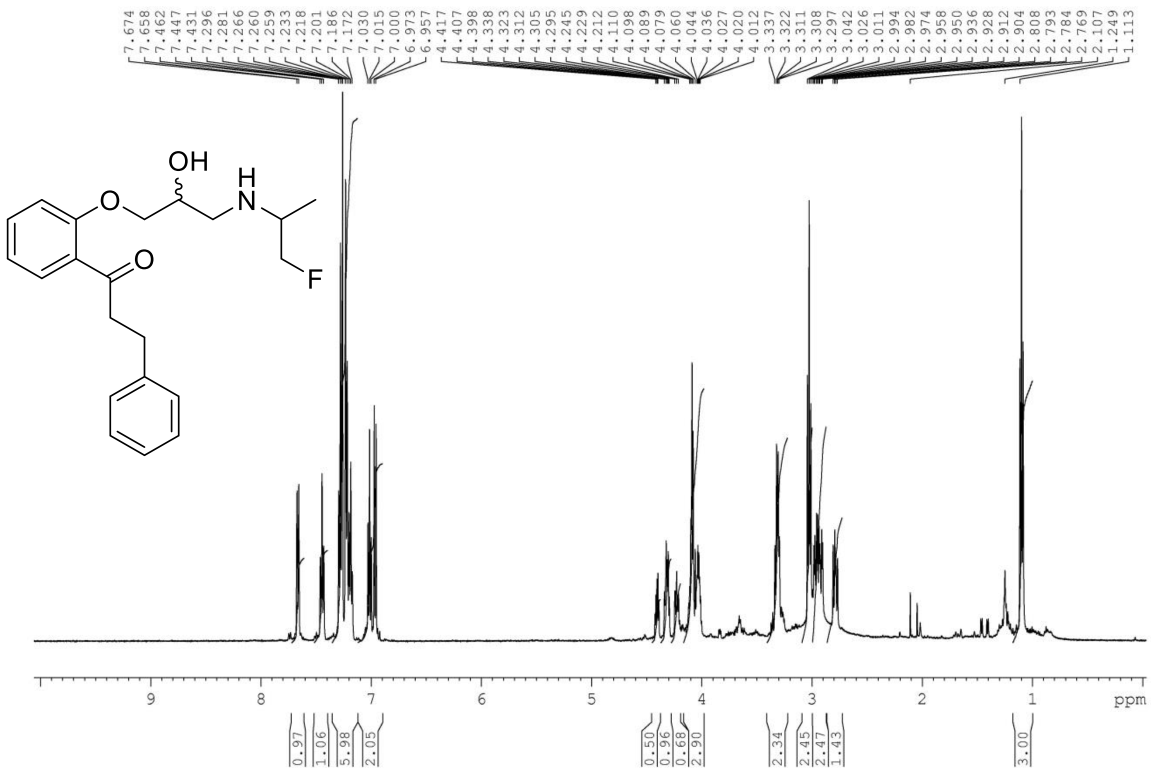
OH
H
N
O
O
F
7.674
7.658
7.462
7.447
7.431
7.296
7.281
7.266
7.260
7.259
7.233
7.218
7.201
7.186
7.172
7.030
7.015
7.000
6.973
6.957
4.417
4.407
4.398
4.338
4.323
4.312
4.305
4.295
4.245
4.229
4.212
4.110
4.098
4.089
4.079
4.060
4.044
4.036
4.027
4.020
4.012
3.337
3.322
3.311
3.308
3.297
3.042
3.026
3.011
2.994
2.982
2.974
2.958
2.950
2.936
2.928
2.912
2.904
2.808
2.793
2.784
2.769
2.107
1.249
1.113
0.97
1.06
5.98
2.05
0.50
0.96
0.68
2.90
2.34
2.45
2.47
1.43
3.00
9
8
7
6
5
4
3
2
1
ppm

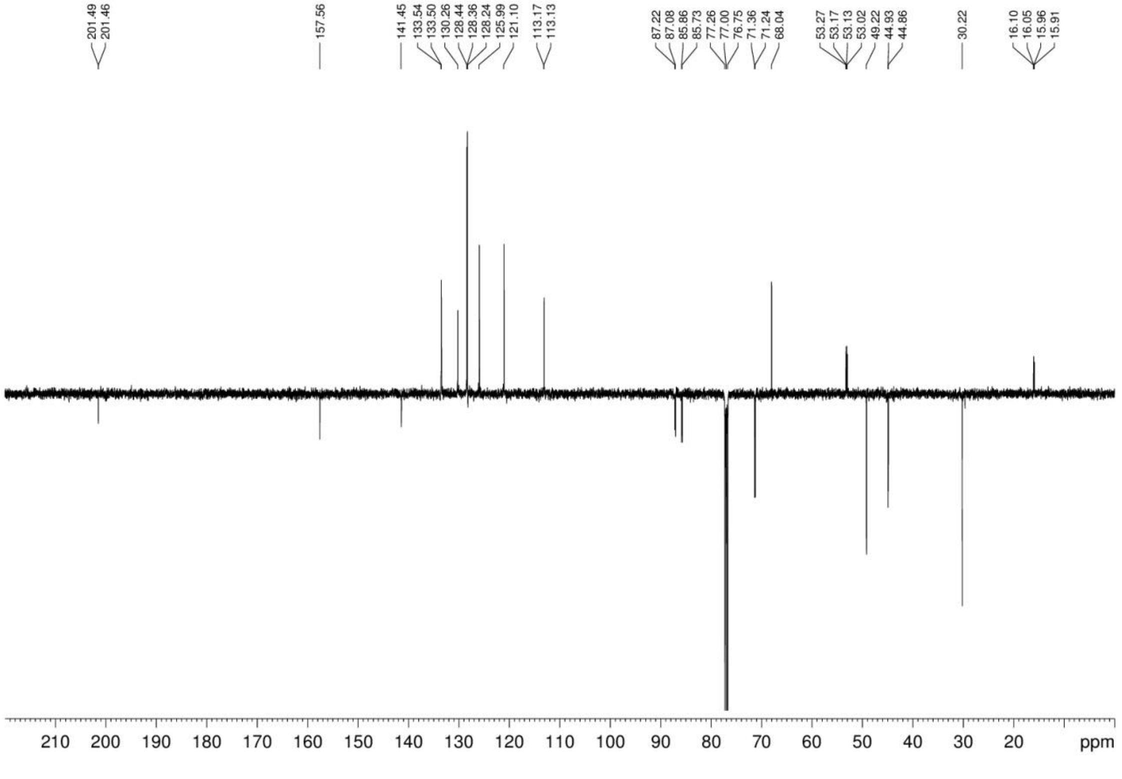
201.49
201.46
157.56
141.45
133.54
133.50
130.26
128.44
128.36
128.24
125.99
121.10
113.17
113.13
87.22
87.08
85.86
85.73
77.26
77.00
76.75
71.36
71.24
68.04
53.27
53.17
53.13
53.02
49.22
44.93
44.86
30.22
16.10
16.05
15.96
15.91
210
200
190
180
170
160
150
140
130
120
110
100
90
80
70
60
50
40
30
20
ppm

16c

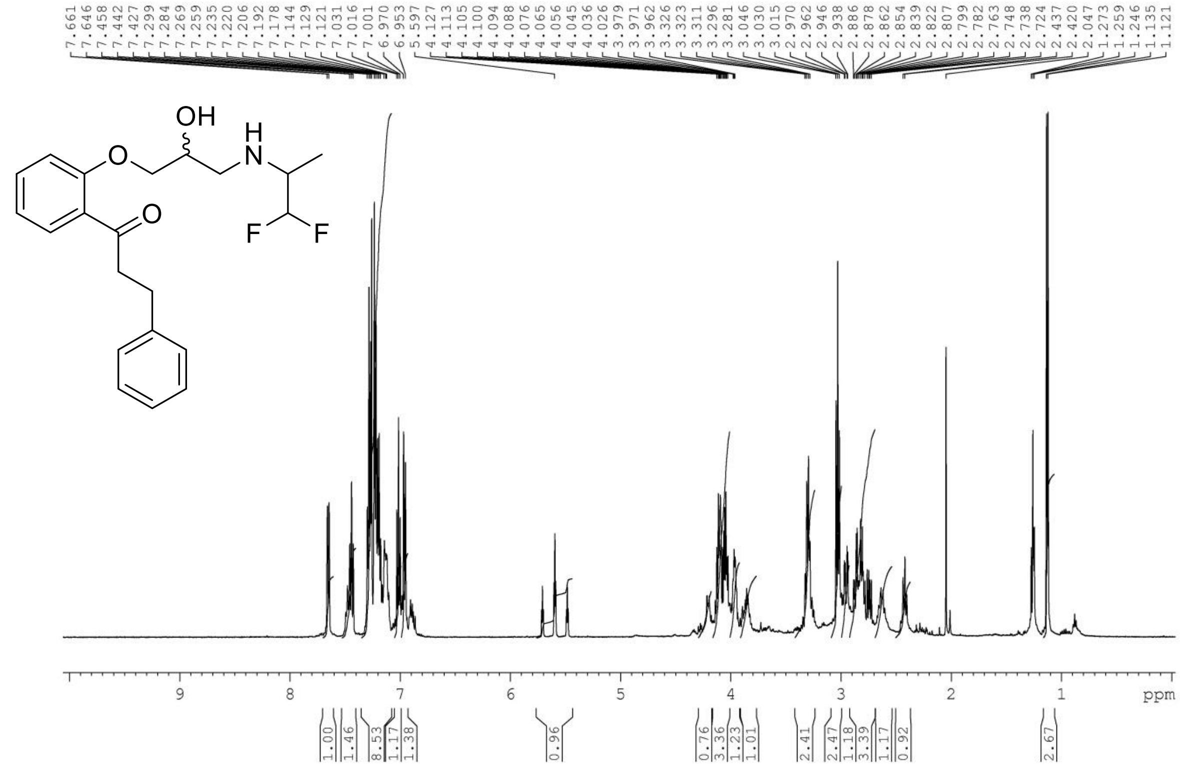
7.661 7.646 7.458 7.442 7.427 7.299 7.284 7.269 7.259 7.235 7.220 7.206 7.192 7.178 7.144 7.129 7.121 7.031 7.016 7.001 6.970 6.953 5.597 4.127 4.113 4.105 4.100 4.094 4.088 4.076 4.065 4.056 4.045 4.036 4.026 3.979 3.971 3.962 3.326 3.323 3.311 3.296 3.281 3.046 3.030 3.015 2.970 2.962 2.946 2.938 2.886 2.878 2.862 2.854 2.839 2.822 2.807 2.799 2.782 2.763 2.748 2.738 2.724 2.437 2.420 2.047 1.273 1.259 1.246 1.135 1.121
OH
H
N
O
O
F
F
9 8 7 6 5 4 3 2 1 ppm
1.00 1.46 8.53 1.17 1.38 0.96 0.76 3.36 1.23 1.01 2.41 2.47 1.18 3.39 1.17 0.92 2.67

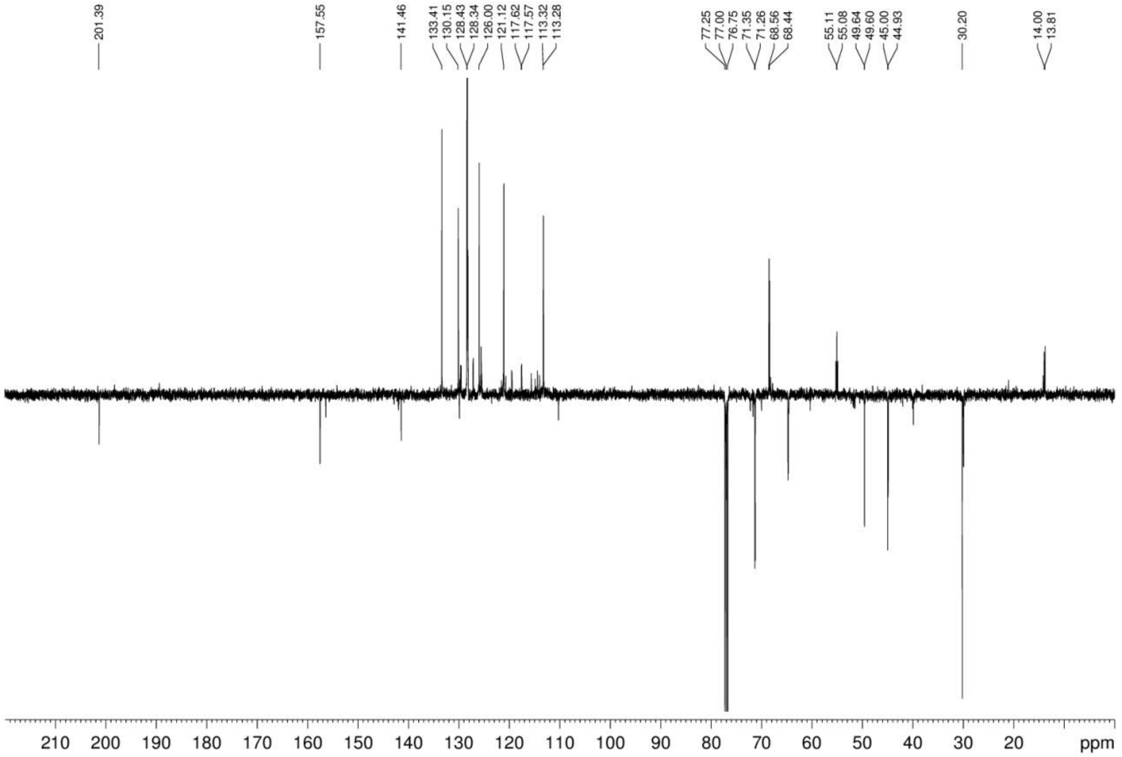
201.39
157.55
141.46
133.41 130.15 128.43 128.34 126.00 121.12 117.62 117.57 113.32 113.28
77.25 77.00 76.75 71.35 71.26 68.56 68.44
55.11 55.08 49.64 49.60 45.00 44.93
30.20
14.00 13.81
210 200 190 180 170 160 150 140 130 120 110 100 90 80 70 60 50 40 30 20 ppm
